# Synthesis, Physicochemical Properties and Molecular Docking of New Benzothiazole Derivatives as Antimicrobial Agents Targeting DHPS Enzyme

**DOI:** 10.3390/antibiotics11121799

**Published:** 2022-12-11

**Authors:** Rasha A. Azzam, Heba A. Elboshi, Galal H. Elgemeie

**Affiliations:** Chemistry Department, Faculty of Science, Helwan University, Cairo 11795, Egypt

**Keywords:** benzothiazole, sulfonamide, antimicrobial evaluation, docking study, physicochemical properties

## Abstract

The drug-resistance problem is widely spread and becoming more common in community-acquired and nosocomial strains of bacteria. Therefore, finding new antimicrobial agents remains an important drug target. From this perspective, new derivatives of benzothiazole were synthesized and evaluated for their antimicrobial activity and ability to inhibit the DHPS enzyme. The synthesis was carried out by the reaction of benzothiazole *N*-arylsulphonylhydrazone with *N*-aryl-2-cyano-3-(dimethylamino)acrylamide, *N*-aryl-3-(dimethylamino)prop-2-en-1-one, arylaldehydes or diazonium salt of arylamine derivatives, which led to the formation of *N*-arylsulfonylpyridones **6a–d** (yield 60–70%) and **12a**–**c** (yield 50–60%)**,** *N*-(2-(benzo[*d*]thiazole-2-yl)-3-arylacryloyl-4-methylsulfonohydrazide **14a**–**c** (yield 60–65%)**,** 4-(benzo[*d*]thiazole-2-yl)-5-aryl-1*H*-pyrazol-3(2*H*)-one **16a**–**c** (yield 65–75%), and *N′*-(2-(benzo[*d*]thiazol-2-yl)-2-(2-arylhydrazono)acetyl)-4-arylsulfonohydrazide **19a**–**e** (yield 85–70%). The antimicrobial evaluations resulted into a variety of microbial activities against the tested strains. Most compounds showed antimicrobial activity against *S. aureus* with an MIC range of 0.025 to 2.609 mM. The most active compound, **16c**, exhibited superior activity against the *S. aureus* strain with an of MIC 0.025 mM among all tested compounds, outperforming both standard drugs ampicillin and sulfadiazine. The physicochemical–pharmacokinetic properties of the synthesized compounds were studied, and it was discovered that some compounds do not violate rule of five and have good bioavailability and drug-likeness scores. The five antimicrobial potent compounds with good physicochemical–pharmacokinetic properties were then examined for their inhibition of DHPS enzyme. According to the finding, three compounds, **16a**–**c**, had IC_50_ values comparable to the standard drug and revealed that compound **16b** was the most active compound with an IC_50_ value of 7.85 μg/mL, which is comparable to that of sulfadiazine (standard drug) with an IC_50_ value of 7.13 μg/mL. A docking study was performed to better understand the interaction of potent compounds with the binding sites of the DHPS enzyme, which revealed that compounds **16a**–**c** are linked by two arene-H interactions with Lys220 within the PABA pocket.

## 1. Introduction

Recently, designing and developing new therapeutic agents to treat microbial infections has emerged as one of the most important goals in the field of medicinal chemistry [1]. This is due to the fact that microbial infections kill over one million people each year. As micro-organisms develop resistance to antimicrobial drugs, the number of deaths rises, affecting people’s health worldwide [2]. Several drugs with heterocyclic rings, such as benzothiazole, have been discovered to have a wide range of pharmaceutical applications and biological activities [3,4,5], due to the extended π-delocalized systems which are capable of binding to DNA molecules via π–π interactions, including anti-inflammatory [6,7], antitumor [8,9,10,11,12], and antiviral [13,14]. In recent years, a considerable number of patents and scientific studies have demonstrated that benzothiazole scaffolds have remarkable antibacterial properties [15,16,17]. For example, pyridine substituted benzothiazoles have been shown to be particularly effective against a variety of bacterial species, including *C. pneumonia*, *E. faecalis*, and *S. aureus* [18]. In addition, benzamide-linked benzothiazole conjugates linked via an ether bond were found to have significant antibacterial activity and low MIC values [18]. Compounds containing an *N*-sulfonamide 2-pyridone derivative substituted with a benzothiazole moiety have shown considerable antibacterial activity against a wide range of bacterial species [19,20]. Furthermore, sulfonamide-containing compounds demonstrated a wide range of biological activities [21,22], including antiviral [22,23,24,25], anticancer [26], and anti-inflammatory activity [27]. Sulfonamide drugs were discovered to have broad antibacterial activity [19] against bacterial infections by inhibiting the dihydropteroate synthase (DHPS) enzyme [20] through competition with 4-aminobenzoic acid (PABA) [28]. Unfortunately, sulfonamide resistance has spread and is becoming more common in community-acquired and nosocomial strains of bacteria, including *S. aureus*, *S. mutans*, and *P. aeruginosa*. This resistance can occur by developing point mutations in the *folP* gene of an organism, which encodes a structural change in DHPS that results in an enzyme with a lower affinity for sulfonamide [29]. As a result, DHPS continues to be a significant drug target, motivating us to develop and synthesize new enzyme inhibitors. In pursuit of that goal, we synthesized new compounds containing benzothiazole and sulfonamide moiety and investigated their antimicrobial activities, DHPS enzyme inhibition, physicochemical properties, and protein interaction using molecular docking. 

## 2. Results and Discussion

### 2.1. Chemistry

**Scheme 1:**
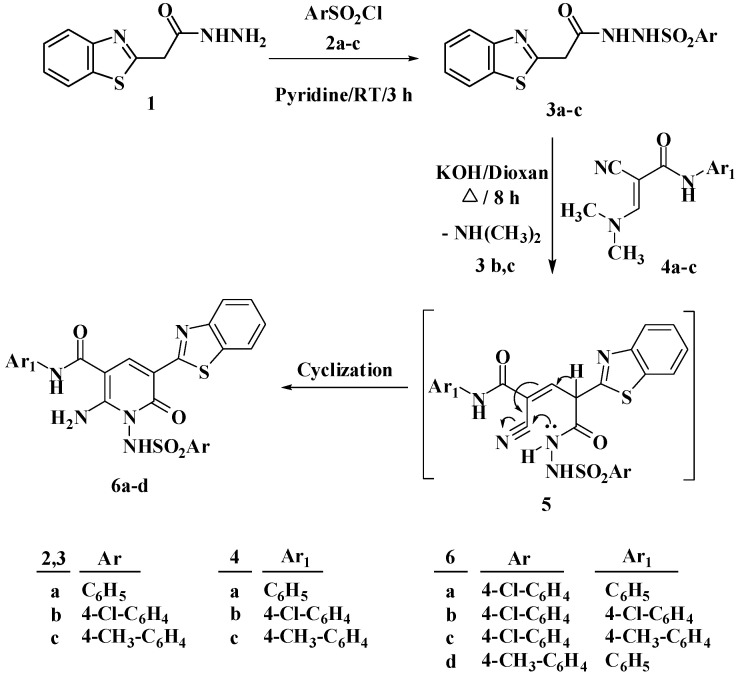
Reaction of *N*-arylsulphonylhydrazones **3a–c** with *N*-aryl-2-cyano-3-(dimethylamino)acrylamide **4a–c** in basic medium.

Derivatives of *N*-arylsulfonylpyridones substituted with benzothiazole moiety **6a**–**d** were synthesized starting from the reaction of *N*-arylsulphonylhydrazones **3a**–**c**, which prepared from the reaction of benzothiazolehydrazide with arylsulfonyl chloride [30], with *N*-aryl-2-cyano-3-(dimethylamino)acrylamide **4a**–**c** [31] in a dry dioxane containing potassium hydroxide, Scheme 1. The reaction may have proceeded via Michael addition and elimination of NH(CH_3_)_2_, intermediate **5**, which followed by the intramolecular cyclization caused by the addition of NH proton to the cyano group to furnish with the substituted *N*-arylsulfonylpyridones **6a**–**d**. On the basis of elemental analysis and spectral data (IR, ^1^H, and _13_C NMR), the structure of compounds **6a**–**d** were determined. According to the IR spectral analysis of compounds **6a**–**d**, the appearance of a broad absorption band at a range of 3426–3437 cm^−1^ confirmed the presence of NH group. In addition, the IR spectra showed a sharp band at a range of 1624–1629 cm^−1^ which corresponding to C=O group. As an illustration, the ^1^H NMR of compound **6a** displayed two singlet signals that were assigned to the protons of the NH group and the CH group of pyidone ring at δ 8.94 and 10.21 ppm, respectively. Additionally, the ^1^H NMR of compound **6a** showed two doublet of doublet but appear triplet signals at δ 7.08 and 7.26 as well as two doublets at δ 7.86 and 7.97 ppm. These four characteristic signals correspond to the four protons of the benzothiazole ring. The two C=O carbons at δ 164.5 and 166.5 ppm were detected in **6b** ^13^C NMR spectrum.

**Scheme 2:**
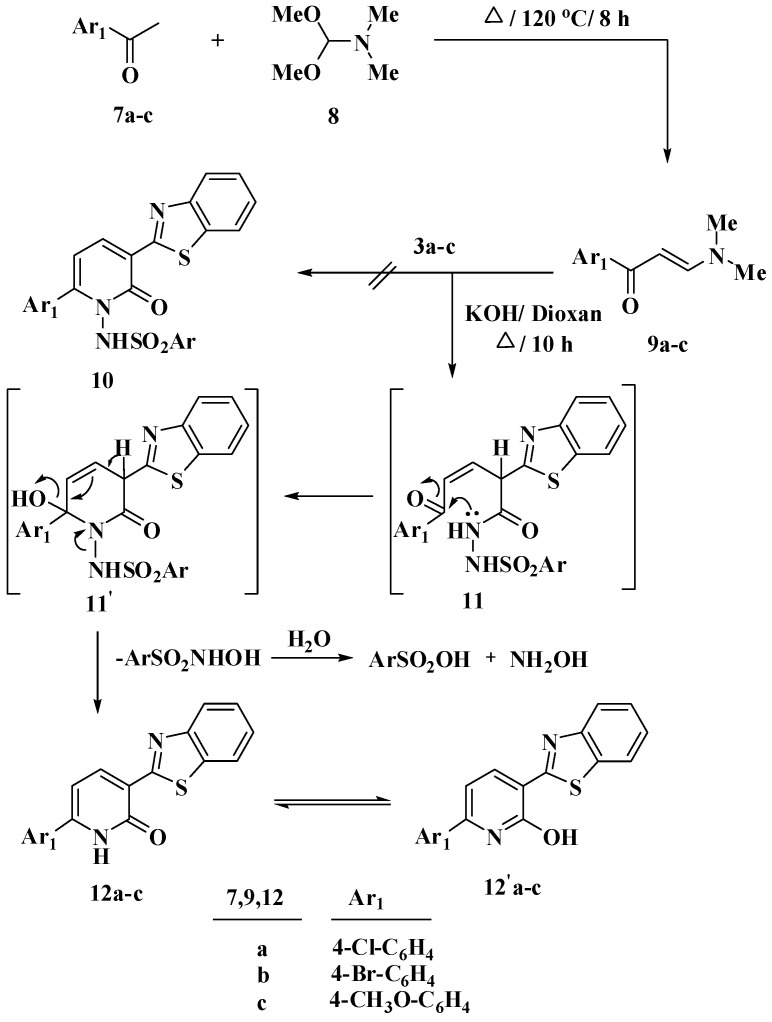
Reaction of *N*-arylsulphonylhydrazones **3a–c** with *N*-aryl-3-(dimethylamino)prop-2-en-1-one **9a–c** in basic medium.

We attempted to synthesize derivatives of benzothiazole substituted with *N*-arylsulfonylpyridones through the reaction of *N*-arylsulphonylhydrazones **3a**–**c** with *N*-aryl-3-(dimethylamino)prop-2-en-1-one **9a**–**c** [32]. The reaction produced the 2-pyridone derivatives **12a**–**c** through the removal of the NH-arylsulphonyl group, intermediate **11** and **11′**, Scheme 2. The produced compounds **12a**–**c** have a tautomerism structure **12′a**–**c** as illustrated in Scheme 2. Both of spectral data (IR, ^1^H NMR and ^13^C NMR) and elemental analysis confirmed the structure of compounds **12a**–**c**, Scheme 2. The IR spectrum of compounds **12a**–**c** confirmed the presence of NH at a range of 3427–3433 cm^−1^ and C=O groups at a range of 1626–1637 cm^−1^. The ^1^H NMR revealed the absence of the signals corresponding to the arylsulfonyl group in all derivatives **12a**–**c**. The ^1^H NMR of compound **12a**, as an example, showed two doublet signals, which assigned for two pyridone-H at δ 6.81 and 8.41 ppm, respectively.

**Scheme 3:**
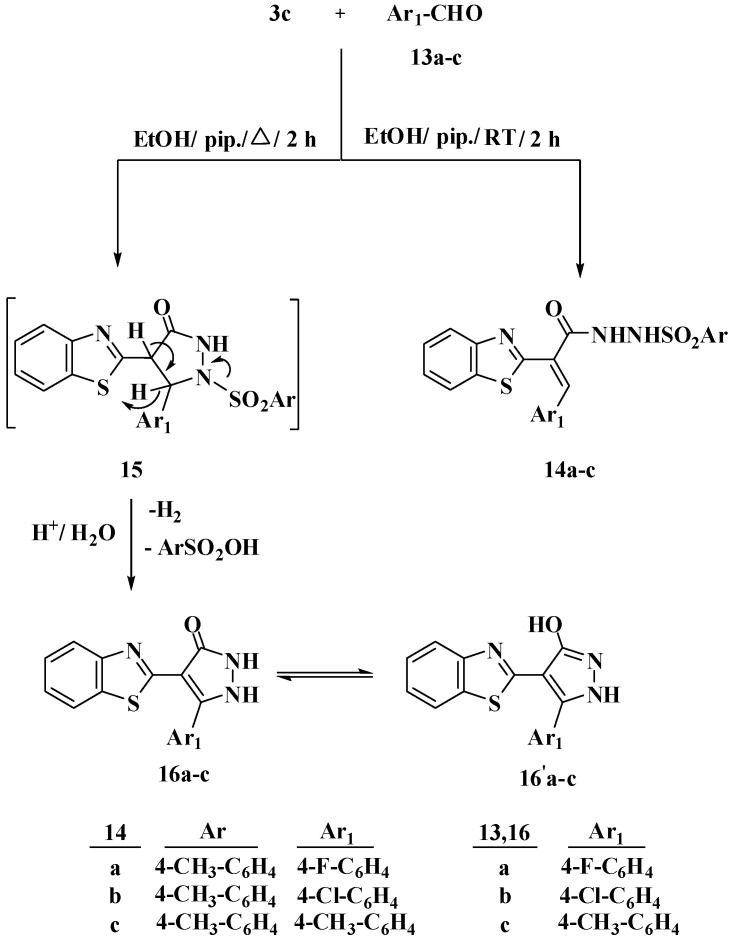
Reaction of *N*-tosylhydrazone derivative **3c** with 4-substituted benzaldehydes **13a**–**c** using a catalytic amount of piperidine at room temperature and under reflux.

Furthermore, the expected compounds *N*-(2-(benzo[*d*]thiazole-2-yl)-3-arylacryloyl-4-methylsulfonohydrazide **14a**–**c** were produced when the *N*-tosylhydrazone derivative **3c** was reacted with 4-substituted benzaldehydes **13a**–**c** in ethanol containing a catalytic amount of piperidine at room temperature. Performing the same reaction under reflux resulted in the formation of pyrazolone derivatives **16a**–**c** via cyclization by addition of NH group to the benzylidine group, intermediate **15**, and removal of the tosyl group after hydrolysis, Scheme 3. When the ^1^H NMR spectra of **14a** and **16b** were compared, a singlet signal at δ 7.61 ppm was observed due to the presence of a proton of CH benzylidine in compound **14a**, whereas this signal was absent in the ^1^H NMR spectrum of compound **16b**. Furthermore, the ^1^H NMR spectra of compound **14a** showed two doublet signals at δ 7.21 and 7.68 ppm, both of which represents two protons that corresponded to tosyl protons, whereas these signals were absent in the ^1^H NMR spectra of compound **16b**. This observation indicates that the tosyl group has been removed from **14a**–**c** after reflux. The produced compounds **16a**–**c** have a tautomerism structure **16′a**–**c**, as illustrated in Scheme 3. The structure of compound **16b** was finally confirmed using LC-MS. The LC-MS of compound **16b** showed a molecular ion peak M+1 at *m*/*z* = 328 and M+3 at *m*/*z* = 330 due to the presence of Cl atom, in the positive mode, as well as M-H at *m*/*z* = 326, in the negative mode. The IR spectra of compounds **14a**–**c** showed the presence of NH and C=O groups at a range of 3434–344 cm^−1^ and 1629–1654 cm^−1,^ respectively. ^13^C NMR spectrum of **14a** confirmed the presence of the C=O carbons at δ 166.0 ppm.

**Scheme 4:**
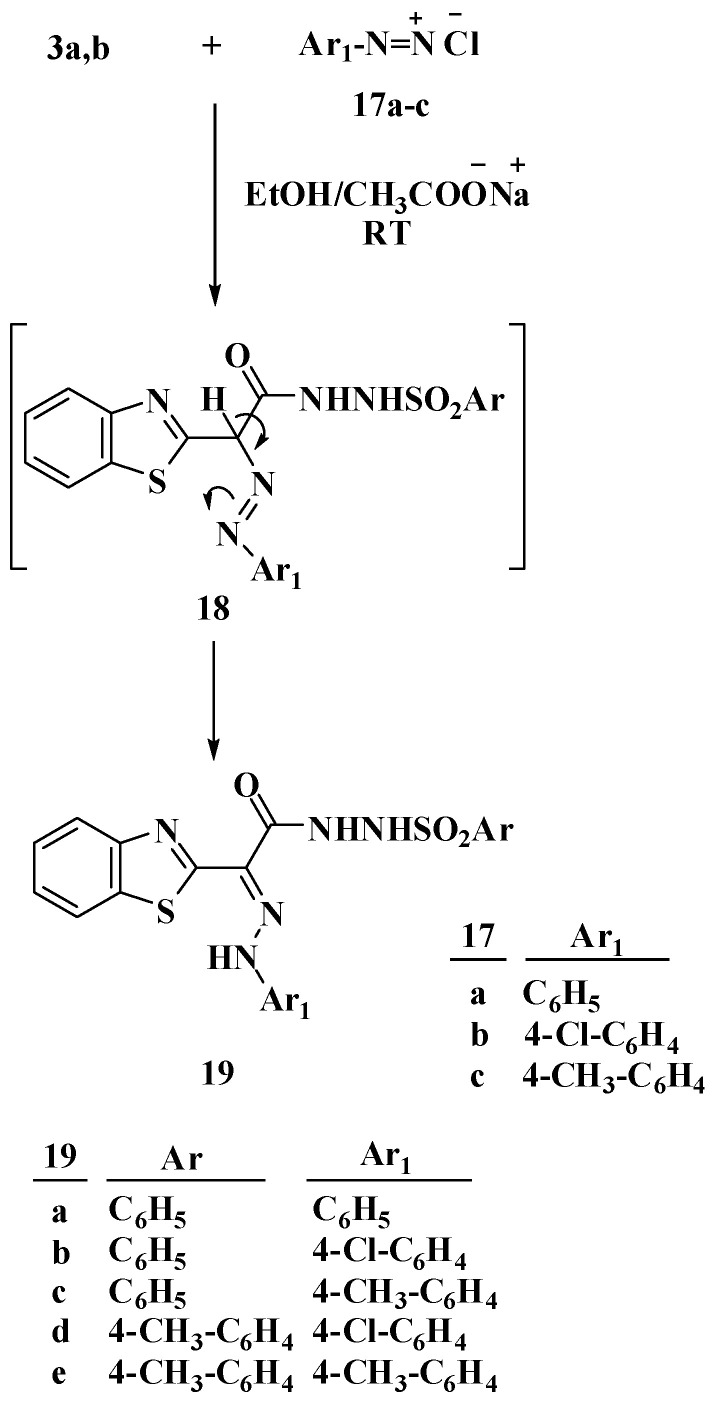
Reaction of *N*-arylsulphonylhydrazones **3a,c** with the diazonium salt of arylamine derivatives **17a**–**c**.

Azodyes were synthesized to further study the reactivity of the methylene group by coupling *N*-arylsulphonylhydrazones **3a,b** with the diazonium salt of arylamine derivatives **17a**–**c**, Scheme 4. In ethanolic sodium acetate, a coupling reaction occurred between active methylene, which acts as a nucleophilic center, and the diazonium salt, which acts as an electrophilic center, yielding compounds **19a**–**e**. To confirm the structure of **19a**–**e**, the compound **19a** was used as an example. The ^1^H NMR spectrum of this compound revealed two doublet peaks at δ 7.81 and 7.89 ppm, both of which represents two protons, confirming the presence of four aromatic amine’s protons. Furthermore, three broad signals appeared at δ 10.00, 10.50 and 14.86 ppm, confirming the presence of three NH group protons. The presence of C=O carbon was confirmed by the appearance of a signal at δ 163.6 ppm in the ^13^C NMR spectrum of **19a**.

### 2.2. Antimicrobial Evaluation

The new synthetic compounds, **6a**–**c**, **12a**–**c**, **14a**–**c**, **16a**–**c** and **19a**–**e**, were tested in vitro to evaluate antibacterial activities against Gram-negative and Gram-positive bacteria, such as *E. coli* (ATCC: 3008), *K. pneumonia* (ATCC: 4415) and *P. aeruginosa* (ATCC: 27853), as an example for Gram-negative bacteria, as well as *S. aureus* (ATCC: 6538) and *S. mutans* (ATCC: 25175) for Gram-positive bacteria. The antifungal activities were evaluated also for the novel compounds in vitro against *C. albicans* (ATCC: 10231). The antibacterial and antifungal activities were determined by the agar-diffusion method using gentamicin, ampicillin and sulfadiazine, example of sulfa drug, as standard drugs against Gram-negative and Gram-positive, while nystatin was used as standard drug against the fungal strain. Table 1 summarizes the results for all tested compounds as the average diameter of inhibition zones of microbial growth around the discs in mm ± SD. The minimum inhibitory concentration (MIC) for the most active compounds was also determined using a twofold serial dilution method, and the results are summarized in Table 2.

Except for two compounds, **19a** and **19e**, all of the tested compounds showed antibacterial activity against the *S. aureus* strain with an MIC value lower than sulfadiazine. Surprisingly, none of the compounds tested were active against *E. coli* strain. It was also observed that only two compounds, **14b** and **16a**, exhibited antibacterial activities against four different bacterial strains, while six compounds showed activities against the fungal strain *C. albicans* strain. Compound **16c** demonstrated superior activity against the *S. aureus* strain with the highest inhibition zone, 40.3 ± 0.6 mm, and the lowest MIC, 0.025 mM, among all tested compounds, outperforming both standard drugs ampicillin and sulfadiazine, which had inhibition zones of 22.0 ± 0.1 and 21.5 ± 0.6 mm and MIC values of 0.179 and 1.998 mM, respectively. Furthermore, the same compound, **16c**, revealed the highest inhibition zone, 31.3 ± 0.6 mm, with low MIC value of 0.203 mM against the *S. mutans* strain among all tested compounds, which is similar to the value of the inhibition zone and MIC of ampicillin and higher than sulfadiazine. Among all tested compounds, the same compound, **16c**, had the highest inhibition zone, 24.3 ± 0.6 mm, with a low MIC value of 0.813 mM against the *K. pneumonia* strain, which is similar to the value of the inhibition zone but with a higher MIC of ampicillin. Not only that, two additional compounds, **14b**, and **14c**, demonstrated inhibition zones against the *S. aureus* strain greater than ampicillin with lower MIC values, while two additional compounds, **16a** and **16b**, showed an inhibition zone lower than ampicillin against the *S. mutans* strain but with a close MIC value. Only one compound, **12a**, exhibited an inhibition zone of 19.6 ± 0.6 mm, similar to sulfadiazine against the *S. aureus* strain but with a lower MIC value of 0.369 mM. 

The structure–activity relationships revealed that compounds containing benzothiazole substituted with pyrazolone ring, **16a**–**c**, have the highest antimicrobial activities followed by compounds containing benzylidine moiety of benzothiazole sulfonylhydrazide, **14a**–**c**. Replacement of pyazolone moiety with pyridone or arylazo moieties led to less potent compounds, **6a**–**c**, **12a**–**c** and **19a**–**e**. 

Among the compounds with a pyrazolone ring, the presence of both 4-chloro and 4-methyphenyl groups bonded to the pyrazolone ring had a positive effect on the antimicrobial activities over the presence of 4-florophenyl group. The activity order can be presented as follows: **16c** > **16b** > **16a**. On the other hand, the presence of both 4-chloro and 4-methylphenyl groups increased the activities of benzylidine moiety of benzothiazole sulfonylhydrazide compounds over the 4-florophenyl group. Therefore, compound activity is determined not only by the type of rings or groups bonded to the benzothiazole ring, but also by the type of substituted phenyl group.

### 2.3. Drug Likeness, and Physicochemical–Pharmacokinetic/ADMET Properties

To investigate the potential of the synthesized compounds as drug candidates, the computed drug likeness, violation of various rules, and ADMET properties were calculated using Molsoft software and the SwissADME program. Poor oral bioavailability of a molecule is more likely in drug discovery when there are more than five hydrogen bond donors, ten hydrogen bond acceptors, a molecular weight greater than 500 g/mol, and a calculated Log *P* greater than 5.

According to the results in Table 3, some compounds, such as **12a**–**c** and **16a**–**c**, have no violations, whereas others have one or more violations. For the number of hydrogen bond donors, number of hydrogen bond acceptors, and Log *P*, none of the compounds exceeded the normal range. The results also revealed that all compounds had a drug-likeness score ranging from -0.45 to 0.71. On the other hand, the molecule weight and topological polar surface area (TPSA) of compounds **6a**–**d** have exceeded the normal range, with Mwt exceeding 500 g/mol and TPSA exceeding 140 Å^2^. Furthermore, compounds **19a**–**e** have more than 140 Å^2^ of TPSA.

The structure–activity relationships revealed that compounds having benzothiazole substituted with the pyrazolone ring, **16a**–**c**, and the pyridone ring, **12a**–**c**, are the most promising drug candidates since they have no violations, a low TPSA and Log Po, and a good drug-likeness score. Furthermore, compounds having the benzylidine moiety of benzothiazole sulfonylhydrazide, **14a**–**c**, could be designated as good drug candidates because they have one violation with a low TPSA, Log Po and a good drug-likeness score.

Moreover, both of blood–brain barrier (BBB) permeant, gastrointestinal (GI) absorption, and bioavailability of the synthesized compounds have also been studied using SwissADME program, Table 4. As shown in Table 3, all of the synthesized compounds have no blood–brain barrier, indicating that they are not BBB permeant. On the other hand, some of the tested compounds, such as **6a**–**d**, **14a**–**c**, and **19a**–**e**, have low GI absorption, indicating a good absorbance in the human intestine, whereas others, such as **12a**–**c** and **14a**–**c**, have a high GI absorption. Furthermore, all of the tested compounds have a bioavailability score of 0.55, except **6b**, indicating that they have good pharmacokinetic properties. 

### 2.4. Enzymatic Assay

Dihydropteroate synthase (DHPS) is an enzyme contributed in the *Bacillus anthracis* folate synthesis pathway via catalyzing PABA to convert into dihydropteroate [33]. Sulfonamides, a class of synthetic molecules that act as competitive inhibitors, are the most commonly used DHPS inhibitors [33]. Other DHPS inhibitors capable of inhibiting DHPS’s pterin binding site can also be utilized [34].

**Figure 1:**
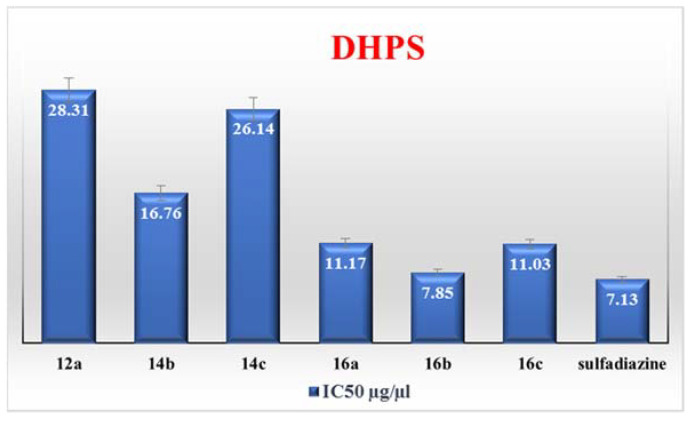
IC_50_ values for the synthesized compounds **12a**, **14b**, **14c, 16a**, **16b** and **16c** against dihydropteroate synthase enzyme.

Based on the antimicrobial evaluation and predicted physicochemical properties of the tested compounds, we chose the most potent compounds, **12a**, **14b**, **14c**, **16a**, **16b**, and **16c**, to be tested for their ability to inhibit DHPS enzyme. Table 5 shows the inhibitory percent against the DHPS enzyme, and Figure 1 shows the IC_50_ of the tested compounds. Sulfadiazine was used as a positive control in this study. The data showed that compound **16b** with 4-chlorophenyl group at C5 position of pyrazolone ring has the lowest IC_50_ value of 7.85 μg/mL, while compound **12a** with 4-chlorophenyl group at C6 position of 3-pyridone ring has the highest IC_50_ value of 28.31 μg/mL. Surprisingly, the IC_50_ value of the most potent compound, **16b**, is comparable to that of sulfadiazine with IC_50_ value 7.13 μg/mL. Compounds with benzylidine moiety, **14b** and **14c**, showed IC_50_ values of 16.76 μg/mL and 26.14 μg/mL, respectively. Additionally, compounds with pyrazolone ring, **16a**–**c**, showed IC_50_ values of 11.17, 7.85 and 11.03 μg/mL, respectively. According to this finding, the presence of a pyrzolone ring bonded to benzothiazole inhibited the DHPS enzyme more than the presence of a pyridone ring or benzylidine moiety bonded to benzothiazole. Furthermore, the reactivity depended on the type of substituent in the para position of the benzene ring. The presence of chlorine atom at the para position of the benzene ring increased the potency of the compounds more than the methyl group. 

Based on the enzyme assay study, we can conclude that three compounds, **16a**–**c**, are potentially effective DHPS inhibitors.

### 2.5. Docking Study

In vitro evaluations of the most potent synthesized compounds revealed that benzothiazole compounds bearing pyrazolone ring, **16a**–**c**, have the potential to inhibit the DHPS enzyme. Therefore, docking studies were conducted using a crystal structure of DHPS (PDB ID: 3TYE) obtained from the Protein Data Bank server to better understand the interaction of these compounds with the binding sites of DHPS enzyme. The DHPS enzyme has two binding pockets, one binds dihydropterin pyrophosphate (DHPP) and the other binds *p*-aminobenzoic acid (PABA). The key amino acid residues that recognized the pterin substrate via hydrogen bonding are Asn120, Asp184, Lys220, and a water molecule, while the amino acid residues that recognized the PABA binding site via arene-H interactions are Lys220 and Phe189 and Ser221 through amino group hydrogen bonds [35]. 

**Figure 2:**
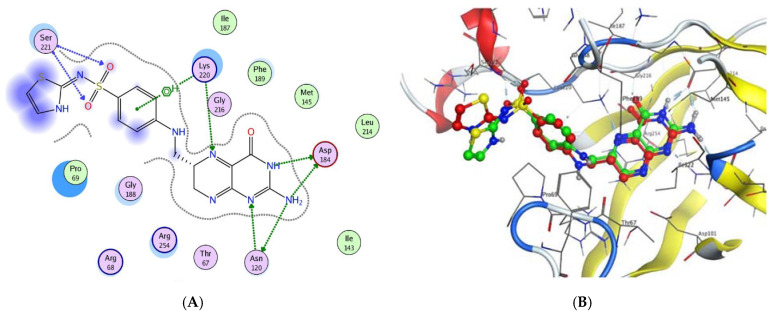
Docking poses of TXZ ligand inside DHPS. (**A**) 2D interaction of TXZ ligand with DHPS pockets. (**B**) 3D Docking of TXZ ligand for validation.

Docking cocrystallized ligand (XTZ) inside the active site after extraction from the respective receptor was used to validate the docking study, as shown in Figure 2. The cocrystallized ligand XTZ docking resulted in a root mean square deviation value of −6.9784 kcal/mol, Table 6. The docking results revealed that XTZ formed four H-bond acceptors with Ser221, Asp184, and Lys220, three H-bond donors with Asp184 and Asn120, and one arene-H interaction with Lys220. On the other hand, Figure 2 depicts the various types of interactions between the ligand XTZ and DHPS pockets. 

**Figure 3:**
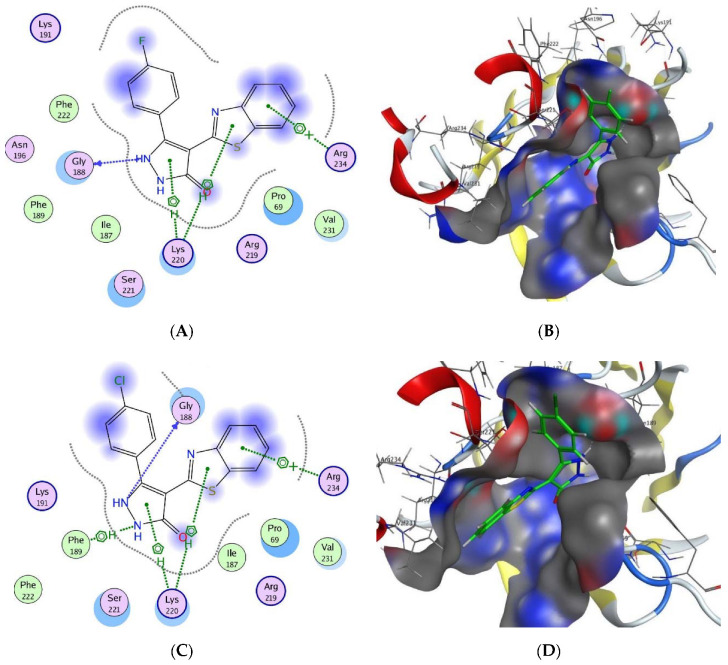

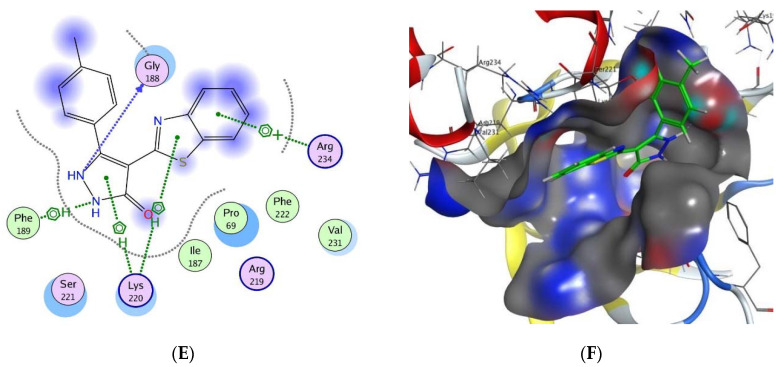
Docking poses of compounds **16a-c** inside DHPS (**A**) 2D interaction of **16a** with DHPS pockets (**B**) 3D interaction of **16a** with DHPS pockets (**C**) 2D interaction of **16b** with DHPS pockets (**D**) 3D interaction of **16b** with DHPS pockets (**E**) 2D interaction of **16c** with DHPS pockets (**F**) 3D interaction of **16c** with DHPS pockets.

The top ranked poses of the most active compounds **16a**–**c** within the active site of DHPS were summarized in Figure 3A–F. Surprisingly, the docking analysis of the evaluated compounds revealed that all three compounds fit inside the PABA pocket. Compound **16a** exhibited three arene-H interactions, two between the pyrazolone and the benzene ring of benzothiazole with Lys220 and one between the thiazole ring with Arg234 in addition to one H-bond donor with bond length 2.22 Å between Gly188 and NH group in the pyrazolone ring, as in Figure 3A,B. Figure 3C–F showed similar interactions in compounds **16b** and **16c** in the active site of DHPS, as well as one extra arene-H interaction between the NH group in the pyrazolone ring and Phe189. Based on these findings, all three compounds are linked not only by one but two arene-H interactions with Lys220 within the PABA pocket. This suggests that these new compounds could be used as alternatives to sulfonamide drugs to combat bacterial resistance.

## 3. Experimental

### 3.1. Chemistry

Melting Points were measured on an SMP3 melting point apparatus. The ^1^H and ^13^C NMR Spectra were recorded on a Bruker advance (III)-400 Spectrometer (400 and 100 MHz, respectively) in DMSO-*d*_6_ using Si(CH_3_)_4_ as an internal standard (See Supplementary Materials) at the Ain Shams University, Cairo, Egypt. Chemical shifts are expressed in δ values (ppm). All coupling constants (*J)* values are given in Hertz. The abbreviations used are as follows: s, singlet; d, doublet and m, multiplet. Reaction courses and product mixtures were routinely monitored by thin layer chromatography (TLC) on silica gel pre-coated F_254_ plates Merck, and using UV lamp.

General procedure for the synthesis of 2-amino-*N*-aryl-5-(benzo[*d*]thiazole-2-yl)-1-(4-chlorophenylsulfonamido)-6-oxo-1,6-dihydropyridine-3-carboxamide (**6a–d**):

*N*-(2-(benzo[*d*]thiazole-2-yl)acetyl)arylsulfonohydrazide (**3b,c**) (10 mmol) was added to a stirred solution of the *N*-aryl-2-cyano-3-(dimethyl amino) acrylamide (5 mmol) in a dry dioxane (30 mL) containing potassium hydroxide (10 mmol). The reaction mixture was refluxed for 8 h and the precipitate was collected by filtration while hot, washed with hot ethanol, dried and crystallized from ethanol.

2-Amino-5-(benzo[*d*]thiazole-2-yl)-1-(4-chlorophenylsulfonamido)-6-oxo-*N*-phenyl-1,6-dihydropyridine-3–carboxamide (**6a**)

Brown solid (yield 65%), m.p. over 350 °C; IR (KBr, cm^−1^): *υ* 3428 (NH-NH_2_), 2925 (Ar-CH), 1624 (C=O); ^1^H NMR (400 MHz, DMSO-*d_6_*): δ 7.08 (t, *J* = 7.6 Hz, 1H, benzothiazole-H), 7.26 (t, *J* = 8.0 Hz, ^1^H, benzothiazole-H), 7.32–7.44 (m, 5H, Ar-H), 7.69 (d, *J* = 8.8 Hz, 2H, Ar-H), 7.78 (d, *J* = 9.2 Hz, 2H, Ar-H), 7.86 (d, *J* = 8.0 Hz, 1H, benzothiazole H), 7.97 (d, *J* = 8.4 Hz, ^1^H, benzothiazole H), 8.94 (s, 1H, CH-pyridine), 10.21 (br, ^1^H, NH); Anal.Calcd.for C_25_H_18_ClN_5_O_4_S_2_ (552.02): calc. C% 54.39, H% 3.29, N% 12.69, found C% 54.41, H% 3.27, N% 12.71.

2-Amino-5-(benzo[*d*]thiazole-2-yl)-*N*-(4-chlorophenyl)-1-(4-chlorophenylsulfonamido)-6-oxo-1,6-dihydropyridine-3–carboxamide (**6b**)

Buff solid (yield 70%), m.p. 334–336 °C; IR (KBr, cm^−1^): *υ* 3426 (NH-NH_2_), 2926 (Ar-CH), 1624 (C=O); ^1^H NMR (400 MHz, DMSO-*d_6_*): δ 7.26 (t, *J* = 7.4 Hz, 1H, benzothiazole-H), 7.39–7.44 (m, ^5^H, 4Ar-H, 1benzothiazole-H), 7.72–7.78 (m, 4H, Ar-H), 7.87 (d, *J* = 10.0 Hz, 1H, benzothiazole-H), 7.97 (d, *J* = 8.4 Hz, 1H, benzothiazole-H), 8.94 (s, H, CH-pyridine), 10.31 (br, 1H, NH); ^13^C NMR (100 MHz, DMSO- *d_6_*): δ 93.4, 105.6, 121.0, 122.0, 122.9, 123.7, 126.1, 127.4, 127.9, 128.5, 128.8, 134.2, 135.1, 135.5, 138.5, 147.2, 152.2, 157.6, 160.1 (Ar-C), 164.5, 166.5 (2C=O); Anal.Calcd.for C_25_H_17_Cl_2_N_5_O_4_S_2_ (586.47): calc. C% 51.20, H% 2.92, N% 11.94, found C% 51.28, H% 2.85, N% 11.90.

2-Amino-5-(benzo[*d*]thiazole-2-yl)-1-(4-chlorophenylsulfonamido)-6-oxo-*N*-(*p*-tolyl)-1,6-dihydropyridine-3–carboxamide (**6c**)

Buff solid (yield 75%), m.p. 302–305 °C; IR (KBr, cm^−1^): *υ* 3427 (NH-NH_2_), 2925 (Ar-CH), 1628 (C=O); ^1^H NMR (400 MHz, DMSO-*d_6_*): δ 2.29 (s, 3H, CH_3_), 7.15 (d, *J* =9.6 Hz, 2H, Ar-H), 7.25 (t, *J* = 7.8 Hz, 1H, benzothiazole-H), 7.38–7.44 (m, 3H, 2Ar-H, 1benzothiazole-H), 7.56 (d, *J* = 11.2 Hz, 2H, Ar-H), 7.77 (d, *J* = 11.6 Hz, 2H, Ar-H), 7.86 (d, *J* = 10.4 Hz, 1H, benzothiazole-H), 7.97 (d, *J* =12.8 Hz, 1H, benzothiazole-H), 8.92 (s, 1H, CH-pyridine), 10.13 (br, 1H, NH); ^13^C NMR (100 MHz, DMSO- *d_6_*): δ 21.9 (CH_3_), 114.5, 121.0, 121.3, 122.0, 123,4, 125.9, 126.8, 127.9, 128.5, 129.3, 129.7, 132.5, 134.0, 135.4, 135.5, 137.2, 147.8, 152.5, 157.6 (Ar-C), 164.7, 166.5 (2C=O); Anal.Calcd.for C_26_H_20_ClN_5_O_4_S_2_ (566.05): calc. C% 55.17, H% 3.56, N% 12.37, found C% 55.19, H% 3.58, N% 12.39.

2-Amino-5-(benzo[*d*]thiazole-2-yl)-1-(4-methylphenylsulfonamido)-6-oxo-*N*-phenyl-1,6-dihydropyridine-3–carboxamide (**6d**)

Brown solid (yield 60%), m.p. over 350 °C; IR (KBr, cm^−1^): *υ* 3414 (NH-NH_2_), 2921 (Ar-CH), 1618 (C=O); ^1^H NMR (400 MHz, DMSO-*d_6_*): δ 2.36 (s, 3H, CH_3_), 7.13 (d, *J* = 7.6 Hz, 2H, Ar-H), 7.27 (t, *J* = 8.4 Hz, 1H, benzothiazole-H), 7.32–7.36 (m, 3H, Ar-H), 7.42 (t, *J* = 7.4 Hz, 1H, benzothiazole-H), 7.67–7.71 (m, 4H, Ar-H), 7.87 (d, *J* = 10.4 Hz, 1H, benzothiazole-H), 7.97 (d, *J* = 8.8 Hz, 1H, benzothiazole-H), 8.94 (s, 1H, CH-pyridine), 10.20 (br, 1H, NH); ^13^C NMR (100 MHz, DMSO- *d_6_*): δ 21.4 (CH_3_), 121.0, 121.3, 121.9, 123.6, 126.0, 126.6, 127.6, 128.5, 128.6, 128.9, 135.4, 135.6, 139.1, 139.9, 145.9, 152.5, 157.8, 160.3 (Ar-C), 164.5, 166.5 (2C=O); Anal.Calcd.for C_26_H_21_N_5_O_4_S_2_ (531.61): calc. C% 58.74, H% 3.98, N% 13.17, found C% 58.77, H% 3.99, N% 13.19.

General procedure for the synthesis of 3-(benzo[*d*]thiazol-2-yl)-6-arylpyridin-2(1*H*)-one (**12a**–**c**):

To a stirred solution of the *N*-aryl-3-(dimethylamino)prop-2-en-1-one (**9a**–**c**) (5 mmol**)** in a dry dioxane (30 mL) containing potassium hydroxide (10 mmol), *N*-(2-(benzo[*d*]thiazole-2-yl)arylsulfonohydrazide **(3a**–**c**) (10 mmol) was added and the reaction mixture was refluxed for 10 h. The precipitate was collected by filtration while hot, washed with hot ethanol, dried and crystallized from ethanol.

3-(Benzo[*d*]thiazol-2-yl)-6-(4-chlorophenyl)pyridin-2(1*H*)-one (**12a**)

Yellow solid (yield 60%), m.p. over 350 °C; IR (KBr, cm^−1^): *υ* 3427 (NH), 2924 (Ar-CH), 1637 (C=O); ^1^H NMR (400 MHz, DMSO-*d_6_*): δ 6.81 (d, *J* = 8.0, 1H, CH-pyridine), 7.26 (t, *J* = 9.4 Hz, 1H, benzothiazole-H), 7.40 (t, *J* = 7.4 Hz, 1H, benzothiazole-H), 7.47 (d, *J* = 10.0 Hz, 2H, Ar-H), 7.85 (d, *J* = 8.0 Hz, 1H, benzothiazole-H), 7.97 (d, *J* = 5.6 Hz, 1H, benzothiazole-H), 8.04 (d, *J* =6.4 Hz, 2H, Ar-H), 8.41 (d, *J* = 8.4 Hz, 1H, CH-pyridine); Anal.Calcd.for C_18_H_11_ClN_2_OS (338.81): calc. C% 63.81, H% 3.27, N% 8.27, found C% 63.83, H% 3.26, N% 8.28

3-(Benzo[*d*]thiazol-2-yl)-6-(4-bromophenyl)pyridin-2(1*H*)-one (**12b**)

Yellow solid (yield 50%), m.p. 345–346 °C; IR (KBr, cm^−1^): *υ* 3429 (NH), 2926 (Ar-CH), 1631 (C=O); ^1^H NMR (400 MHz, DMSO-*d_6_*): δ 6.78 (d, *J* = 10.4 Hz, 1H, CH-pyridine), 7.39–7.46 (m, 2H, benzothiazole-H), 7.60 (d, *J* = 8.8 Hz, ^2^H, Ar-H), 7.82 (d, *J* = 5.6 Hz, 1H, benzothiazole-H), 7.88 (d, *J* = 9.6 Hz, 1H, benzothiazole-H), 8.10 (d, *J* = 6.4 Hz, 2H, Ar-H), 8.36 (d, *J* = 7.2 Hz, 1H, CH-pyridine); Anal.Calcd.for C_18_H_11_BrN_2_OS (383.26): calc. C% 56.41, H% 2.89, N% 7.31, found C% 56.42, H% 2.88, N% 7.34.

3-(Benzo[*d*]thiazol-2-yl)-6-(4-methoxyphenyl)pyridin-2(1*H*)-one (**12c**)

Yellow solid (yield 60%), m.p. over 350 °C; IR (KBr, cm^−1^): *υ* 3428 (NH), 2926 (Ar-CH), 1626 (C=O); ^1^H NMR (400 MHz, DMSO-*d_6_*): δ 2.50 (s, 3H, OCH_3_), 7.14–7.98 (m, 10H, 4Ar-H, 4benzothiazole-H, 2CH-pyridine); Anal.Calcd.for C_19_H_14_N_2_O_2_S (334.39): calc. C% 68.24, H% 4.22, N% 8.38, found C% 68.27, H% 4.23, N% 8.39.

General procedure for the synthesis of *N*-(2-(benzo[*d*]thiazole-2-yl)-3-arylacryloyl-4-methylbenzenesulfonohydrazide (**14a**–**c**):

To a stirred solution of the para derivatives of benzaldehyde (**13a**–**c**) (10 mmol) in ethanol (30 mL) containing piperidine (1 ml), *N-*(2-(benzo[*d*]thiazole-2-yl)acetyl) arylsulfonohydrazide (**3c**) (10 mmol) was added and the reaction mixture was stirred at room temperature for 4 h. The solid precipitate was filtered off and then recrystallized from ethanol.

*N*-(2-(Benzo[*d*]thiazole-2-yl)-3-(4-florophenylacryloyl)-4-methylbenzene-sulfonohydrazide (**14a**)

White solid (yield 65%), m.p. 201–202 °C; IR (KBr, cm^−1^): ʋ 3440 (NH), 2925 (Ar-CH), 1629 (C=O); ^1^H NMR (400 MHz, DMSO-*d_6_*): δ 2.40 (s, 3H, CH_3_), 7.21–7.26 (m, 2H, Ar-H), 7.36 (d, *J* = 8.4 Hz, 2H, Ar-H), 7.47 (t, *J* = 7.4 Hz, 1H, benzothiazole-H), 7.56 (t, *J* = 8.4 Hz, 1H, benzothiazole-H), 7.61 (s, 1H, CH), 7.68–7.72 (m, 2H, Ar-H), 7.78 (d, *J* = 8.0 Hz, 2H, Ar-H), 8.00 (d, *J* = 7.6 Hz, 1H, benzothiazole-H), 8.12 (d, *J* = 7.6 Hz, 1H, benzothiazole-H), 10.20 (br, 1H, NH), 10.71 (br, 1H, NH); ^13^C NMR (100 MHz, DMSO-*d_6_*): δ 21.4 (CH_3_), 116.1 (d, *J* = 28 Hz, C-F), 116.3, 122.6, 123.1, 126.2, 127.2, 128.2, 129.7, 130.1 (d, *J* = 10 Hz, C-F), 130.5, 132.5, 132.6, 134.6, 137.1, 143.7, 153.4 (Ar-C), 165.7 (d, *J* = 230 Hz, C-F), 166.0 (C=O); Anal.Calcd.for C_23_H_18_FN_3_O_3_S_2_ (467.54): calc. C% 50.09, H% 3.88, N% 8.99, found C% 50.11, H% 3.89, N% 8.92.

*N*-(2-(Benzo[*d*]thiazole-2-yl)-3-(4-chlorophenylacryloyl)-4-methylbenzene-sulfonohydrazide (**14b**)

White solid (yield 60%), m.p. 188–189 °C; IR (KBr, cm^−1^): ʋ 3444 (NH), 2926 (Ar-CH), 1630 (C=O); ^1^H NMR (400 MHz, DMSO-*d_6_*): δ 2.37 (s, 3H, CH_3_), 7.36–7.77 (m, 13H, 8Ar-H, 4benzothiazole-H, 1CH), 10.23 (br, 1H, NH), 10.71 (br, 1H, NH); ^13^C NMR (100 MHz, DMSO-*d_6_*): δ 21.5 (CH_3_), 1227, 123.2, 126.3, 127.9, 128.1, 128.5, 129.2, 129.7, 131.0, 131.8, 132.8, 134.5, 134.7, 137.1, 143.7, 153.5, 165.6 (Ar-C), 165.8 (C=O); Anal.Calcd.for C_23_H_18_ClN_3_O_3_S_2_ (483.99): calc. C, 57.08, H, 3.75, N, 8.68, found C% 57.09, H% 3.76, N% 8.69.

*N*-(2-(Benzo[*d*]thiazole-2-yl)-3-(*p*-tolyl)acryloyl)-4-methylbenzene-sulfonohydrazide (**14c**)

White solid (yield 60%), m.p. 195–196.5 °C; IR (KBr, cm^−1^): ʋ 3439 (NH), 2926 (Ar-CH), 1654 (C=O); ^1^H NMR (400 MHz, DMSO-*d_6_*): δ 2.37 (s, 3H, CH_3_), 2.38 (s, 3H, CH_3_), 7.22 (d, *J* = 8.8 Hz, 2H, Ar-H), 7.35–7.42 (m, 3H, 2Ar-H, benzothiazole-H), 7.46 (t, *J* = 8.2 Hz, 1H, benzothiazole-H), 7.53–7.57 (m, 3H, 2Ar-H, 1CH), 7.80 (d, *J* = 9 Hz, 2H, Ar-H), 8.01 (d, *J* = 8.0 Hz, 1H, benzothiazole-H), 8.10 (d, *J* = 8.0 Hz, 1H, benzothiazole-H), 10.15 (br, 1H, NH), 10.70 (br, 1H, NH); Anal.Calcd.for C_24_H_21_N_3_O_3_S_2_ (463.57): calc. C% 62.18, H% 4.57, N% 9.06, found C% 62.19, H% 4.56, N% 9.09.

General procedure for the synthesis of 5-aryl-4-(benzo[*d*]thiazole-2-yl)-1*H*-pyrazol-3(2*H*)-one (**16a**–**c**):

To a stirred solution of the para derivatives of benzaldehyde **(13a**–**c**) (10 mmol) in ethanol (30 mL) containing piperidine (1 mL), *N-*(2-(benzo[*d*]thiazole-2-yl)acetyl)-4-methylsulfonohydrazide **(3c)** (10 mmol) was added and the reaction mixture was refluxed for 4 h. After cooling, the reaction mixture poured in water and neutralized by HCl. The precipitate formed was collected by filtration, washed with hot ethanol, dried and crystallized from ethanol.

4-(Benzo[d]thiazole-2-yl)-5-(4-florophenyl)-1H-pyrazol-3(2H)-one (16a)

Buff solid (yield 65%), m.p. 238–240 °C; IR (KBr, cm^−1^): *υ* 3437 (NH), 2929 (Ar-CH), 1631 (C=O); ^1^H NMR (400 MHz, DMSO-*d_6_*): δ 7.31–7.37 (m, 3H, 2Ar-H, 1benzothiazole-H), 7.42 (t, *J* = 7.4 Hz, 1H, benzothiazole-H), 7.79–7.85 (m, 3H, 2Ar-H, 1benzothiazole-H), 8.01 (d, *J* = 7.6 Hz, 1H, benzothiazole-H), 12.53 (br, 1H, NH); ^13^C NMR (100 MHz, DMSO-*d_6_*): δ 115.6 (d, *J* = 28 Hz, C-F), 121.7, 122.0, 124.5, 126.4, 126.7, 131.7 (d, *J* = 8 Hz, C-F), 133.8, 152.4, 160.9 (Ar-C), 161.5 (d, *J* = 245 Hz, C-F), 164.2 (C=O); Anal.Calcd.for C_16_H_10_FN_3_OS (311.33): calc. C% 61.73, H% 3.24, N% 13.50, found C% 61.75, H% 3.25, N% 13.52.

4-(Benzo[*d*]thiazole-2-yl)-5-(4-chlorophenyl)-1*H*-pyrazol-3(2*H*)-one (**16b**)

Buff solid (yield 75%), m.p. 236–237.5 °C; IR (KBr, cm^−1^): *υ* 3439 (NH), 2924 (Ar-CH), 1631 (C=O); ^1^H NMR (400 MHz, DMSO-*d_6_*): δ 7.32 (t, *J* = 8.8 Hz, 1H, benzothiazole-H), 7.43 (t, *J* = 8.4 Hz, 1H, benzothiazole-H), 7.57 (d, *J* = 6.8 Hz, 2H, Ar-H), 7.80- 7.82 (m, 3H, 2Ar-H, 1benzothiazole-H), 8.02 (d, *J* = 8.0 Hz, 1H, benzothiazole-H), 10.69 (br, 1H, NH); LC-MS: *m*/*z* 328; Anal.Calcd.for C_16_H_10_ClN_3_OS (327.79): calc. C% 58.63, H% 3.07, N% 12.82, found C% 58.65, H% 3.08, N% 12.84.

4-(Benzo[*d*]thiazole-2-yl)-5-(*p*-tolyl)-1*H*-pyrazol-3(2*H*)-one (**16c**)

Buff solid (yield 70%), m.p. 239–241 °C; IR (KBr, cm^−1^): ʋ 3432 (NH), 2929 (Ar-CH), 1629 (C=O); ^1^H NMR (400 MHz, DMSO- *d_6_*): δ 2.35 (s, 3H, CH_3_), 7.05 (t, *J* = 7.2 Hz, 1H, benzothiazole-H), 7.15 (d, *J* = 8.0 Hz, 2H, Ar-H), 7.21 (t, *J* = 7.0 Hz, 1H, benzothiazole-H), 7.46 (d, *J* = 7.6 Hz, 1H, benzothiazole-H), 7.77 (d, *J* = 6.4 Hz, 1H, benzothiazole-H), 7.94 (d, *J* = 7.2 Hz, 2H, Ar-H); ^13^C NMR (100 MHz, DMSO-*d_6_*): δ 21.4 (CH_3_), 119.6, 119.9, 121.1, 121.6, 125.0, 128.1, 129.1, 133.5, 136.1, 153.4, 163.6 (Ar-C), 165.8 (C=O); Anal.Calcd.for C_17_H_13_N_3_OS (307.37): calc. C% 66.43, H% 4.26, N% 13.67, found C% 66.46, H% 4.27, N% 13.68.

General procedure for the synthesis of *N′*-(2-(benzo[*d*]thiazol-2-yl)-2-(2-arylhydrazono)acetyl)-4-arylsulfonohydrazide (**19a**–**e**):

Aniline derivatives (10 mmol) were dissolved in a concentrated hydrochloric acid (2 mL) in an ice bath. Sodium nitrite (10 mmol) was dissolved in cold water (0 °C) and added dropwise to the reaction mixture under vigorous mechanical stirring. The diazonium salts (**17a**–**c**) were obtained and used for the next coupling reaction. *N-*(2-(Benzo[*d*]thiazole-2-yl)acetyl)arylsulfonohydrazide (**3a,c**) (5 mmol) was dissolved in ethanol. Freshly prepared diazonium salt was added dropwise to the reaction mixture under vigorous mechanical stirring (0 °C). After addition, the mixture was neutralized with sodium acetate anhydrous to pH 7, and the precipitate was filtered, washed with ethanol and dried in a vacuum. The product was recrystallized from ethanol.

*N′*-(2-(Benzo[*d*]thiazol-2-yl)-2-(2-phenylhydrazono)acetyl)benzenesulfono-hydrazide (**19a**)

Yellow solid (yield 85%) m.p. 246–248.6 °C; IR (KBr, cm^−1^): ʋ 3438 (NH), 2925 (Ar-CH), 1633 (C=O); ^1^H NMR (400 MHz, DMSO-*d_6_*): δ 7.17 (t, *J* = 7.4 Hz, 1H, benzothiazole-H), 7.44–7.52 (m, 3H, Ar-H), 7.67 (t, *J* = 7.0 Hz, 1H, benzothiazole-H), 7.81 (d, *J* = 7.6 Hz, 2H, Ar-H), 7.89 (d, *J* = 6.4 Hz, 2H, Ar-H), 8.13 (d, *J* = 8.4 Hz, 1H, benzothiazole-H), 8.28 (d, *J* = 7.6 Hz, 1H, benzothiazole-H), 10.02 (br, 1H, NH), 10.58 (br, 1H, NH), 14.84 (br, 1H, NH); ^13^ C NMR (100 MHz, DMSO-*d_6_*): δ 116.5, 121.8, 122.3, 123.1, 124.7, 126.6, 127.0, 128.1, 129.3, 129.8, 133.4, 134.2, 139.8, 142.4, 150.6, 158.4 (Ar-C), 163.6 (C=O); Anal.Calcd.For C_21_H_17_N_5_O_3_S_2_ (451.52): calc. C% 55.86, H% 3.79, N% 15.51, found C% 55.89, H% 3.78, N% 15.53.

*N′*-(2-(Benzo[*d*]thiazol-2-yl)-2-(2-(4-chlorophenyl)hydrazono)acetyl)benzene-sulfonohydrazide (**19b**)

Yellow solid (yield 85%,) m.p. 265–267 °C; IR (KBr, cm^−1^): ʋ 3445 (NH), 2925 (Ar-CH), 1632 (C=O); ^1^H NMR (400 MHz, DMSO-*d_6_*): δ 7.27–7.67 (m, 7H, 5Ar-H, 2benzothiazole-H), 7.86–7.88 (m, 4H, Ar-H), 8.13 (d, *J* = 8.4 Hz, 1H, benzothiazole-H), 8.28 (d, 1H, *J* = 7.6 Hz, benzothiazole-H), 10.02 (br, 1H, NH), 10.58 (br, 1H, NH), 14.84 (br, 1H, NH); Anal.Calcd.For C_21_H_16_ClN_5_O_3_S_2_ (485.97): calc. C% 51.90, H% 3.32, N% 14.41, found C% 51.93, H% 3.33, N% 14.43.

*N′*-(2-(Benzo[*d*]thiazol-2-yl)-2-(2-(4-methylphenyl)hydrazono)acetyl)benzene-sulfonohydrazide (**19c**)

Yellow solid (yield 85%,) m.p. 262–263 °C; IR (KBr, cm^−1^): ʋ 3440 (NH), 2925 (Ar-CH), 1648 (C=O); ^1^H NMR (400 MHz, DMSO-*d_6_*): δ 2.29 (s, 3H, CH_3_), 7.26 (d, *J* = 6.8 Hz, 2H, Ar-H), 7.48 (t, *J* = 7.6 Hz, 1H, benzothiazole-H), 7.57–7.59 (m, 3H, 2Ar-H, 1benzothiazole-H), 7.65–7.70 (m, 3H, 2Ar-H, 1benzothiazole-H), 7.88 (d, *J* = 9.6 Hz, 2H, Ar-H), 8.12 (d, *J* = 8.0 Hz, 1H, benzothiazole-H), 8.25 (d, *J* = 10.4 Hz, 1H, benzothiazole-H), 9.98 (br, 1H, NH), 10.47 (br, 1H, NH), 14.87 (br, 1H, NH); ^13^ C NMR (100 MHz, DMSO-*d_6_*): δ 21.0 (CH_3_), 116.5, 121.2, 122.3, 123.0, 126.5, 126.9, 128.1, 129.3, 130.3, 133.4, 134.0, 134.2, 139.9, 140.1, 150.6, 158.5 (Ar-C), 163.7 (C=O); Anal.Calcd.For C_22_H_19_N_5_O_3_S_2_ (465.55): calc. C% 56.76, H% 4.11, N% 15.04, found C% 56.78, H% 4.12, N% 15.06.

***N′***-(2-(Benzo[*d*]thiazol-2-yl)-2-(2-(4-chlorophenyl)hydrazono)acetyl)-4-methylbenzenesulfono-hydrazide (**19d**)

Yellow solid (yield 70%,) m.p. 280–281 °C; IR (KBr, ʋ cm^−1^): 3445 (NH), 2925 (Ar-CH), 1632 (C=O); ^1^H NMR (400 MHz, DMSO-*d_6_*): δ 2.36 (s, 3H, CH_3_), 7.35–7.57 (m, 6H, 4 Ar-H, 2benzothiazole-H), 7.77 (d, *J* = 7.8 Hz, 2H, Ar-H), 7.84 (d, *J* = 7.2 Hz, 2H, Ar-H), 8.11 (d, *J* = 5.6 Hz, 1H, benzothiazole-H), 8.25 (d, *J* = 8.4 Hz, 1H, benzothiazole-H), 9.85 (br, 1H, NH), 10.52 (br, 1H, NH), 14.81 (br, 1H, NH); Anal.Calcd.For C_22_H_18_ClN_5_O_3_S_2_ (499.99): calc. C% 52.85, H% 3.63, N% 14.01, found C% 52.88, H% 3.64, N% 14.0.

*N′*-(2-(Benzo[*d*]thiazol-2-yl)-2-(2-(4-methylphenyl)hydrazono)acetyl)-4-methylbenzenesulfono-hydrazide (**19e**)

Yellow solid (yield 85%) m.p. 285–286 °C; IR (KBr, cm^−1^): ʋ 3442 (NH), 2924 (Ar-CH), 1636 (C=O); ^1^H NMR (400 MHz, DMSO-*d_6_*): δ 2.88 (s, 3H, CH_3_), 2.41 (s, 3H, CH_3_), 7.25 (d, *J* = 7.2 Hz, 2H, Ar-H), 7.36 (d, *J* = 8.0 Hz, 2H, Ar-H), 7.48 (t, *J* = 7.2 Hz, 1H, benzothiazole-H), 7.57 (t, *J* = 7.8 Hz, 1H, benzothiazole-H), 7.70 (d, *J* = 7.6 Hz, 2H, Ar-H), 7.76 (d, *J* = 8.4 Hz, 2H, Ar-H), 8.12 (d, *J* = 8.8 Hz, 1H, benzothiazole-H), 8.25 (d, *J* = 7.6 Hz, 1H, benzothiazole-H), 9.91 (br, 1H, NH), 10.41 (br, 1H, NH), 14.83 (br, 1H, NH); Anal.Calcd.For C_23_H_21_N_5_O_3_S_2_ (479.57): calc. C% 57.60, H% 4.41, N% 14.60, found C% 57.62, H% 4.42, N% 14.62.

### 3.2. Antimicrobial Activity

The antimicrobial activity of synthesized compounds was determined using agar well diffusion method [36]. All the compounds were tested in vitro for their antibacterial activity against *S. aureus* and *S. mutans* (Gram-positive bacteria), *E. coli*, *P. aeruginosa* and *K. pneumonia* (Gram-negative bacteria) using nutrient agar medium. Ampicillin and gentamicin were used as standard drugs for Gram-positive and Gram-negative, respectively. DMSO was used as solvent control. The compounds were tested at a concentration of 15 mg/mL against both bacterial and fungal strains. The sterilized media was poured onto the sterilized Petri dishes (20–25 mL, each petri dish) and allowed to solidify at room temperature. Microbial suspension was prepared in sterilized saline equivalent to McFarland 0.5 standard solution (1.5 × 10^5^ CFU mL^−1^) and its turbidity was adjusted to OD= 0.13 using spectrophotometer at 625 nm. Optimally, within 15 min after adjusting the turbidity of the inoculum suspension, a sterile cotton swab was dipped into the adjusted suspension and was flooded on the dried agar surface, then allowed to dry for 15 min with lid in place. Wells of 6 mm diameter was made in the solidified media with the help of sterile borer. A total of 100 μL of the solution of the tested compound was added to each well with the help of micropipette. The plates were incubated at 37 °C for 24 h in case of antibacterial activity. This experiment was carried out in triplicate and zones of inhibition were measured in mm scale.

### 3.3. Methodology of MIC

For each strain, three to five isolated colonies were selected from the fresh agar plate and were transferred into a tube containing 3–4 mL of sterile broth medium. The bacterial suspension was mixed well and incubated at 35–37 °C for 2–6 h. The turbidity of the bacterial suspension should be equal to or greater than the turbidity of a McFarland Standard 0.5. After that, 1 mg of the tested compound (antimicrobial agent) was dissolved in 1 mL DMSO and two-fold serial dilution was done using broth medium. A fixed volume of the prepared bacterial inoculum was added to each tube and incubated for at 37 °C 16–20 h. The MIC is defined as the lowest concentration of the antimicrobial agent that inhibits visible growth of the tested isolate as observed with the unaided eye [37].

### 3.4. Drug Likeness Predictions and Physicochemical–Pharmacokinetic/ADMET Properties

A qualitative concept used in drug design to predict a drug-like property is drug-likeness. Drug-like properties, such as solubility, permeability, transporter effects, and metabolic stability are critical for drug candidates’ success. They have an impact on oral bioavailability, toxicity, metabolism, clearance, and in vitro pharmacology. Five separate filters were used to evaluate the drug-likeness of the synthesized compounds, Lipinski [38], Veber [39], Muegge [40], Ghose [41] and Egan [42] rules in addition to bioavailability, and the drug-likeness scores using the Molsoft software and SwissADME program.

### 3.5. Enzyme Assay

DHPS catalytic assay DHPS was assayed for the most potent compounds. Assay tubes contained 5 mM MgCl_2_, 5 m MDTT, 10AM H_2_PtCH_2_0PP, 1 mm [3H] PABA (finalspact, 2Ci/mmol), and 40 mm Tris/HCI, pH8.3 in a total reaction volume of 100 mL. Various concentrations of inhibitors were added when applicable. Reactions were initiated by the addition of 0.5 mU of enzyme (1U= 1 nmol of product formed/min). After a 30-min incubation at 37 °C the reaction tubes were placed on ice to terminate the reactions and then spotted onto 3×30 cm strips of 3 mm chromatography paper (Whatman Laboratory Products Inc.). The strips were developed in a descending chromatography tank using an elution buffer of 0.1 M KH_2_PO_4_, pH 7.0. The origins containing the labeled products of the reaction were cut from the strips, placed in scintillation vials, and counted in a liquid scintillation counter (Packard Tri-Carb; Packard Instrument Co., Inc., Downers Grove, IL, USA) 24 h after the addition of 9.5 mL of counting cocktail (3A70b; Research Products International Corp., Mt.Pros-pect, IL, USA). A total of 100 uL of lysate (100 μg of protein) was combined with ^14^C-labeled PABA, diphosphoric acid, and mono[(2-amino1,4,7,8-tetrahydro-4-oxo-6-pteridinyl)-methyl]ester in Tris buffer, pH 8.3, containing dithiothreitol and MgCl_2_ in a 200-μL reaction volume, and the mixture was held at 37 °C for 15 min. One hundred microliters of each reaction volume was spotted onto 3 mm paper (Whatman International, Ltd., Maidstone, England), and ascending chromatography was performed in 0.01 M phosphate buffer. Under these conditions, free substrate (^14^C-labeled PABA) migrates with the solvent front and radiolabeled product (^14^C-labeled dihydropteroate) remains at the origin. The chromatogram was dried, the area (1 cm^2^) representing the origin was placed into scintillation fluid, and radioactivity was measured using an LS 6000IC Liquid Scintillation System (Beckman Instruments, Fullerton, Calif.). Results were expressed as the mean and standard deviation of triplicate samples in picomoles of product formed per milligram of total protein.

### 3.6. Molecular Docking Study

The Molecular Operating Environment (MOE 2014) was used for the molecular studies. The ligand molecules were drawn by builder molecule and their energy was minimized. All of the minimizations were done until an rmsd gradient of 0.01 kcal/mol was reached with the MMFF94X force field, and the partial charges were calculated automatically. Docking simulations were run using the Protein Data Bank’s crystal structure of the DHPS enzyme in complex with XTZ (PDB ID: 3TYE). Water molecules and ligands that were bound were removed. The MOE protonate 3D application was used to add the missing hydrogens and to correctly assign the ionization states. The MOE-Alpha site finder was used to generate the active site. The obtained alpha spheres were used to create dummy atoms. Ligands were then docked within the active sites using the MOE-Dock. The GBVI/WSA DG free-energy estimates were used to rank the optimized poses, and docking poses were visually examined. The interactions with binding pocket residues were finally investigated.

## 4. Conclusions

Eighteen new benzothiazole derivatives were synthesized and tested for their antimicrobial activity against six microbial strains. Most compounds showed antimicrobial activity against *S. aureus* with MIC range in 0.025 to 2.609 mM. The most active compound **16c** exhibited superior activity against the *S. aureus* strain with an MIC value of 0.025 mM among all tested compounds, outperforming both standard drugs, ampicillin and sulfadiazine, as well as having very good activity against *S. mutans* and *K. pneumonia* with MIC values of 0.203 and 0.813 mM, respectively. The predicted physicochemical properties, bioavailability and drug-likeness scores indicated that six compounds **12a**–**c** and **16a**–**c** have not violated any rule with TPSA range of 73.99–89.78, and three compounds violate only one rule with TPSA 124.78. The bioavailability score of all compounds was approximately 0.55. The enzyme assay of potent compounds revealed that benzothiazole derivative **16b**, having a pyrzolone ring and 4-chlorophenyl moiety, was the most active compound with an IC_50_ value of 7.85 μg/mL, which is comparable to that of sulfadiazine (standard drug) with IC_50_ value of 7.13 μg/mL. Finally, the docking study reveals that compounds **16a**–**c** are linked by two arene-H interactions with Lys220 within the PABA pocket.

## Figures and Tables

**Table 1:** Antibacterial inhibition zone in mm ± standard deviation of synthesized compounds.

Compd No.	Diameter of the Inhibition Zone (mm)					
	Gram (+ve) Bacteria		Gram (−ve) Bacteria			Fungi
	*S. aureus*	*S. mutans*	*K. pneumonia*	*P. aeruginosa*	*E. coli*	*C. albicans*
**6a**	19.6 ± 0.6	12.3 ± 0.6	^a^ -	-	-	-
**6b**	18.3 ± 0.6	15.3 ± 0.5	-	-	-	-
**6c**	10.6 ± 0.5	10.6 ± 0.5	-	-	-	-
**12a**	19.6 ± 0.6	-	-	-	-	-
**12b**	17.6 ± 0.6	-	-	-	-	-
**12c**	15.6 ± 0.5	11.3 ± 0.5	-	-	-	-
**14a**	19.3 ± 0.6	18.3 ± 0.6	-	-	-	20.6 ± 0.6
**14b**	31.6 ± 0.6	21.6 ± 0.6	15.6 ± 0.6	24.3 ± 0.6	-	-
**14c**	30.3 ± 0.6	20.3 ± 0.6	-	-	-	11.3 ± 0.5
**16a**	15.3 ± 0.5	25.0 ± 0.6	17.3 ± 0.6	16.6 ± 0.5	-	12.3 ± 0.5
**16b**	35.3 ± 0.6	24.6 ± 0.6	-	25.6 ± 0.6	-	10.6 ± 0.5
**16c**	40.3 ± 0.6	31.3 ± 0.6	24.3 ± 0.6	-	-	-
**19a**	-	-	-	-	-	10.3 ± 0.5
**19b**	11.6 ± 0.5	-	-	-	-	-
**19c**	11.0 ± 1.0	-	-	16.0 ± 1.0	-	11.6 ± 0.5
**19d**	-	-	-	21.6 ± 0.6	-	-
**19e**	-	-	-	-	-	-
**Gentamicin**	^b^ NT	NT	25.0 ± 0.5	30.0 ± 0.5	27.0 ± 0.5	NT
**Ampicillin**	22.0 ± 0.1	30.0 ± 0.5	NT	NT	NT	NT
**Nystatin**	NT	NT	NT	NT	NT	21.0 ± 0.5
**Sulfadiazine**	21.5 ± 0.6	22.2 ± 1.1	15.9 ± 0.9	20.6 ± 1.4	21.4 ± 0.4	NT

^a^ No activity; ^b^ Not tested.

**Table 2:** The minimum inhibitory concentration (MIC, mM) of the most active compounds.

Compd No.	The Minimum Inhibitory Concentration (MIC mM)			
	*S. aureus*	*S. mutans*	*K. pneumonia*	*P. aeruginosa*
**6a**	0.906	NT ^a^	NT	NT
**6b**	1.705	1.705	NT	NT
**12a**	0.369	NT	NT	NT
**12b**	2.609	NT	NT	NT
**14a**	1.069	2.139	NT	NT
**14b**	0.129	0.258	1.033	0.517
**14c**	0.135	0.539	NT	NT
**16a**	1.606	0.200	0.803	1.606
**16b**	0.191	0.191	NT	0.762
**16c**	0.025	0.203	0.813	NT
**19c**	NT	NT	NT	2.148
**19d**	NT	NT	NT	0.500
**Standard**	**Ampicillin**		**Gentamicin**	
	0.179	0.179	0.065	0.131
	**Sulfadiazine**			
	1.998	0.499	0.999	0.999

^a^ Not tested.

**Table 3:** Predicted drug likeness, number of violation of various rules and bioavailability of the synthesized compounds.

NO	Mwt ^a^	Number of HBD ^b^	Number of HBA ^c^	Log *P*o/w (iLOGP) ^d^	Lipinski, Ghose, Veber, Egan and Muegge Violations	TPSA ^e^	Drug-Likeness Model Score
**6a**	552.02	3	5	2.55	1, 2, 1, 1, 1	172.80	0.71
**6b**	586.47	3	5	2.76	2, 3, 1, 2, 2	172.80	0.68
**6c**	566.05	3	5	2.61	1, 2, 1, 1, 1	172.80	0.54
**6d**	531.61	3	5	3.24	1, 2, 1, 1, 1	172.80	0.07
**12a**	338.81	1	2	3.18	0	73.99	0.04
**12b**	383.26	1	2	3.27	0	73.99	−0.032
**12c**	334.39	1	3	3.24	0	83.22	−0.21
**14a**	467.54	2	6	2.70	0, 1, 0, 0, 1	124.78	0.017
**14b**	483.99	2	5	2.53	0, 2, 0, 0, 1	124.78	0.24
**14c**	463.57	2	5	3.28	0, 0, 0, 0, 1	124.78	−0.13
**16a**	311.33	2	3	2.15	0	89.78	−0.12
**16b**	327.79	2	2	2.35	0	89.78	0.00
**16c**	307.37	2	2	2.39	0	89.78	−0.44
**19a**	451.52	2	7	2.26	0, 0, 1, 1, 1	149.50	−0.45
**19b**	485.97	2	7	2.78	0, 1, 1, 1, 1	149.50	0.14
**19c**	465.55	2	7	2.37	0, 0, 1, 1, 1	149.50	−0.042
**19d**	499.99	7	2	2.28	0, 2, 1, 1, 1	149.50	0.11
**19e**	479.57	2	7	3.16	0, 1, 1, 1, 1	149.50	−0.29

^a^ Molecular weight, ^b^ Number of hydrogen bond donors, ^c^ Number of hydrogen bond acceptors, ^d^ Lipophilicity, ^e^ Topological polar surface area.

**Table 4:** Predicted ADMET properties of the tested compounds.

NO	GI Absorption ^a^	BBB Permeant ^b^	BA ^c^
**6a**	Low	No	0.55
**6b**	Low	No	0.17
**6c**	Low	No	0.55
**6d**	Low	No	0.55
**12a**	High	No	0.55
**12b**	High	No	0.55
**12c**	High	No	0.55
**14a**	Low	No	0.55
**14b**	Low	No	0.55
**14c**	Low	No	0.55
**16a**	High	No	0.55
**16b**	High	No	0.55
**16c**	High	No	0.55
**19a**	Low	No	0.55
**19b**	Low	No	0.55
**19c**	Low	No	0.55
**19d**	Low	No	0.55
**19e**	Low	No	0.55

^a^ GI gastrointestinal system, ^b^ Blood–brain barrier, ^c^ Bioavailability score.

**Table 5:** Inhibition % values of compounds **12a**, **14b, 14c, 16a, 16b, 16c** and sulfadiazine against DHPS enzyme.

Compd No.	100 (μg/mL)	10 (μg/mL)	1 (μg/mL)	0.1 (μg/mL)
**12a**	58	44	27	11
**14b**	65	47	24	12
**14c**	59	46	21	8.6
**16a**	70	49	26	12
**16b**	72	52	31	16
**16c**	68	48	31	13
**Sulfadiazine**	75	56	26	11

**Table 6:** Molecular docking free binding energy estimates to DHPS.

Compd	Binding E. (kcal/mol)	Residues Involved in Arene-H Interactions	Residues Involved in H-Bond Interactions
**16a**	−5.2350	Lys220, Arg234	Gly188 (2.22 Å)
**16b**	−5.4236	Lys220, Phe189, Arg234	Gly188 (2.21 Å)
**16c**	−5.4405	Lys220, Phe189, Arg234	Gly188 (2.20 Å)
**TXZ**	−6.9784	Lys220	Ser221 (1.75 & 2.5 Å), Lys 220 (1.88 Å), Asn120 (2.31 & 2.11 Å), Asp184 (1.74 & 1.95 Å)

## Data Availability

Not applicable.

## Supplementary Materials

The following are available online at https://www.mdpi.com/article/10.3390/antibiotics11121799/s1, Supplementary Materials: IR, ^1^H NMR, and ^13^C NMR spectra of new compounds, **6a**–**d**, **12a**–**c**, **14a**–**c**, **16a**–**c**, and **19a**–**e**.
