# Supplementary material for: Synthesis, Physicochemical Properties and Molecular Docking of New Benzothiazole Derivatives as Antimicrobial Agents Targeting DHPS Enzyme
Source: Antibiotics (Basel). 2022 Dec 11;11(12):1799. doi: 10.3390/antibiotics11121799 (PMC9774648; doi:10.3390/antibiotics11121799)
Supplement: Supplementary file 1 [file antibiotics-11-01799-s001.zip › antibiotics-1961280-SI.pdf]

antibiotics

**Supplementary Materials**

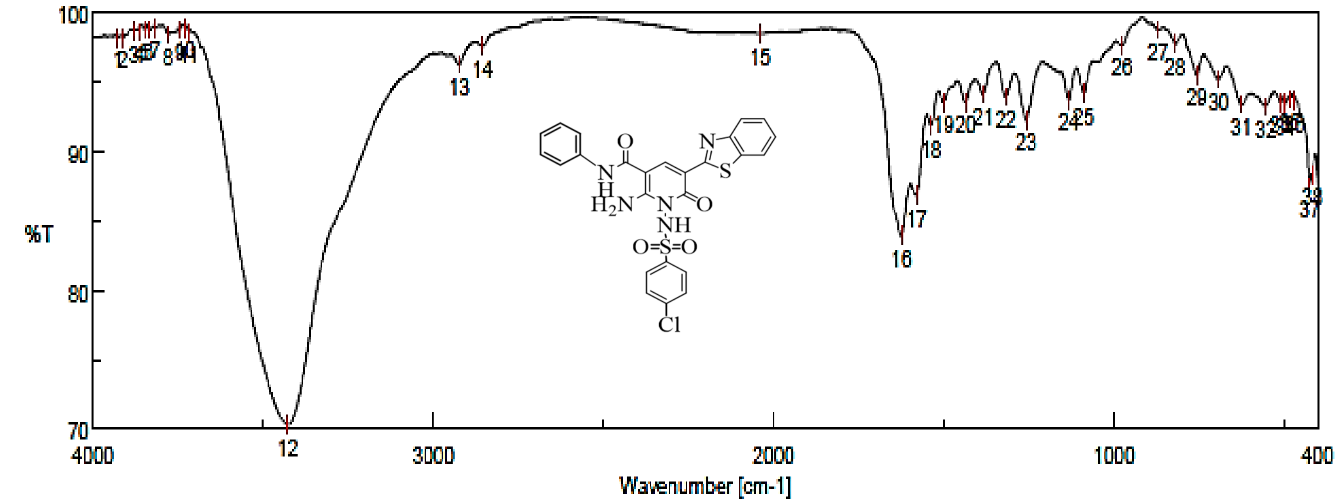


[ Result of Peak Picking ]

| No. | Position | Intensity | No. | Position | Intensity | No. | Position | Intensity | No. | Position | Intensity |
|---|---|---|---|---|---|---|---|---|---|---|---|
| 1 | 3927.32 | 98.2283 | 2 | 3912.86 | 98.2096 | 3 | 3880.08 | 98.6903 | 4 | 3863.68 | 98.6728 |
| 5 | 3848.26 | 98.7736 | 6 | 3836.68 | 98.7822 | 7 | 3816.44 | 98.9016 | 8 | 3778.83 | 98.4677 |
| 9 | 3745.08 | 98.7875 | 10 | 3729.66 | 98.9392 | 11 | 3720.01 | 98.6372 | 12 | 3428.81 | 70.2728 |
| 13 | 2925.48 | 96.2631 | 14 | 2856.06 | 97.6107 | 15 | 2040.32 | 98.5373 | 16 | 1624.73 | 83.9286 |
| 17 | 1579.41 | 86.8266 | 18 | 1536.99 | 91.8624 | 19 | 1500.35 | 93.5445 | 20 | 1435.74 | 93.5328 |
| 21 | 1384.64 | 94.1278 | 22 | 1318.11 | 93.9589 | 23 | 1257.36 | 92.2671 | 24 | 1132.01 | 93.7253 |
| 25 | 1088.62 | 94.2059 | 26 | 976.769 | 97.6394 | 27 | 869.739 | 98.7906 | 28 | 819.598 | 97.7627 |
| 29 | 755.959 | 95.5005 | 30 | 691.355 | 95.2183 | 31 | 624.823 | 93.3559 | 32 | 553.47 | 93.2384 |
| 33 | 510.08 | 93.485 | 34 | 496.58 | 93.4417 | 35 | 483.081 | 93.7615 | 36 | 469.582 | 93.6562 |
| 37 | 424.263 | 87.4938 | 38 | 415.585 | 88.3182 | | | | | | |

**Figure S1.** IR spectrum of compound **6a.**

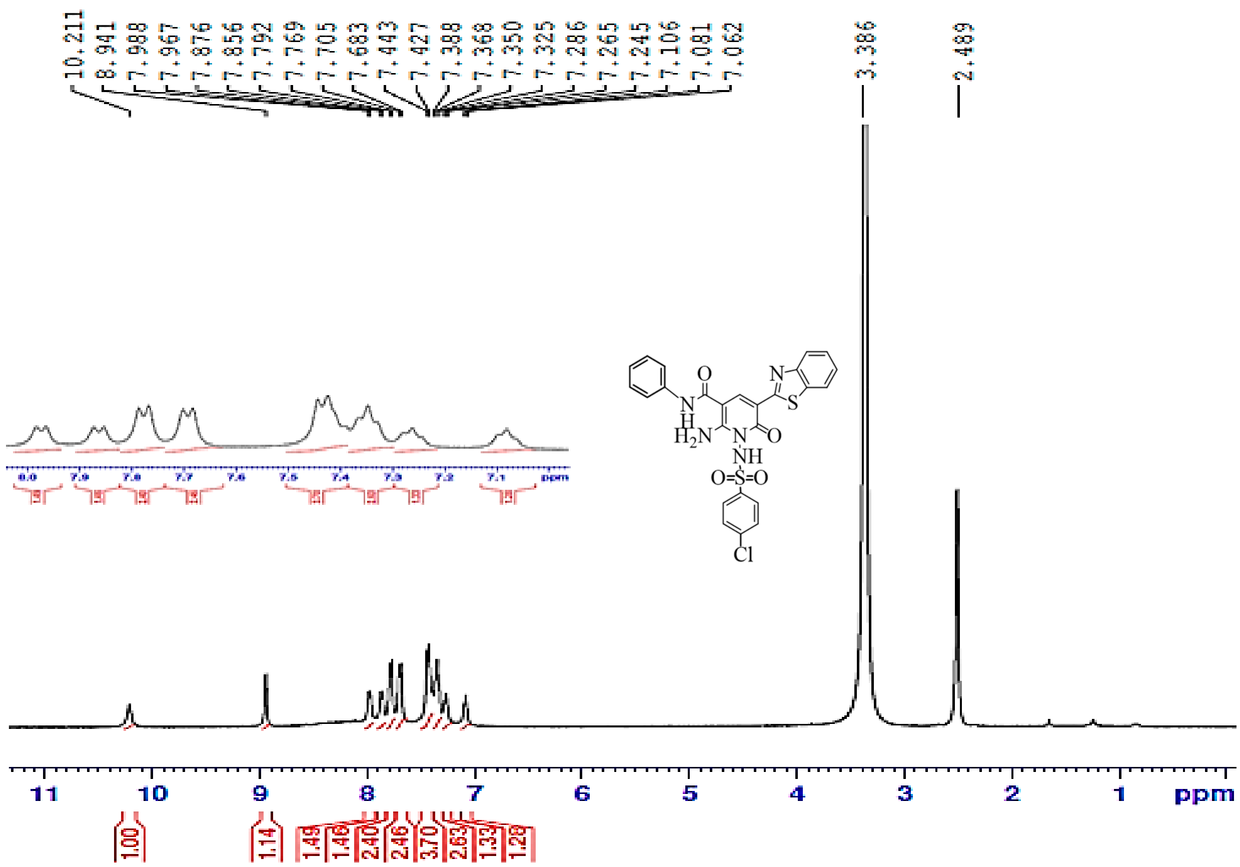

**Figure S2.** $^{1}$H NMR spectrum of compound **6a.**

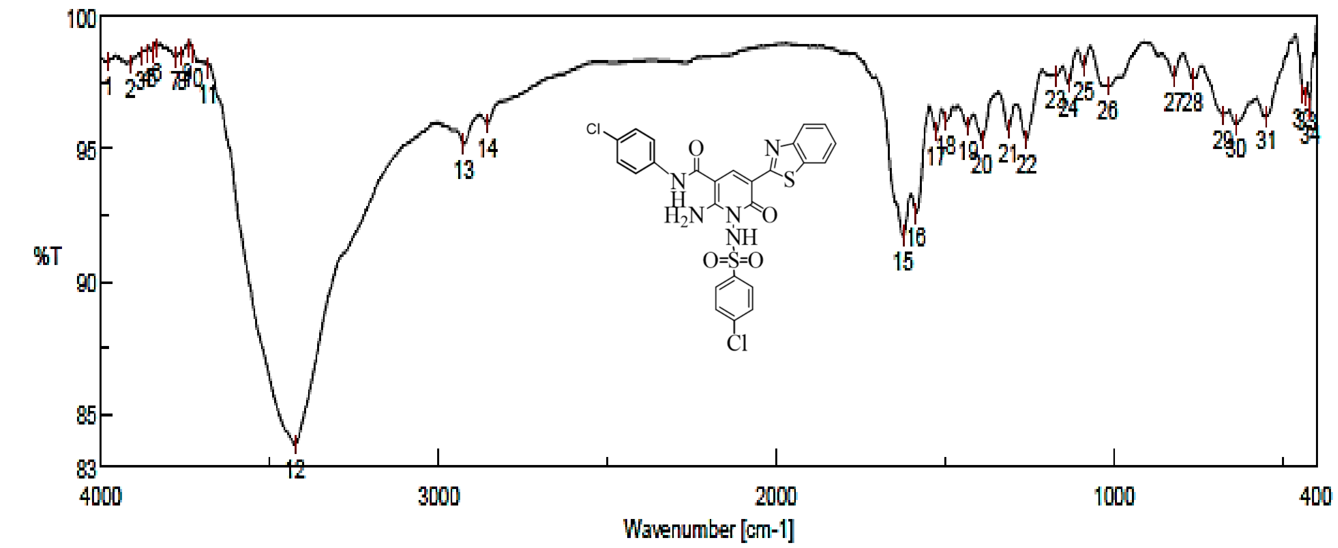


[ Result of Peak Picking ]

| No. | Position | Intensity | No. | Position | Intensity | No. | Position | Intensity | No. | Position | Intensity |
|---|---|---|---|---|---|---|---|---|---|---|---|
| 1 | 3979.39 | 98.2917 | 2 | 3912.86 | 98.1939 | 3 | 3882.97 | 98.5118 | 4 | 3863.68 | 98.5969 |
| 5 | 3848.26 | 98.6344 | 6 | 3835.72 | 98.8169 | 7 | 3778.83 | 98.4617 | 8 | 3761.47 | 98.4892 |
| 9 | 3744.12 | 98.8299 | 10 | 3728.69 | 98.6028 | 11 | 3684.34 | 98.0427 | 12 | 3426.89 | 83.8444 |
| 13 | 2926.45 | 95.1878 | 14 | 2857.02 | 95.964 | 15 | 1624.73 | 91.7229 | 16 | 1586.16 | 92.5652 |
| 17 | 1526.38 | 95.6468 | 18 | 1498.42 | 96.0431 | 19 | 1431.89 | 95.875 | 20 | 1390.42 | 95.3328 |
| 21 | 1313.29 | 95.7479 | 22 | 1258.32 | 95.3638 | 23 | 1171.54 | 97.7543 | 24 | 1133.94 | 97.4894 |
| 25 | 1089.58 | 98.128 | 26 | 1018.23 | 97.3752 | 27 | 821.527 | 97.7276 | 28 | 767.53 | 97.6521 |
| 29 | 676.892 | 96.2656 | 30 | 639.287 | 95.9322 | 31 | 548.649 | 96.2086 | 32 | 439.69 | 97.0501 |
| 33 | 430.048 | 96.9875 | 34 | 418.477 | 96.5285 | | | | | | |

**Figure S3.** IR spectrum of compound **6b.**

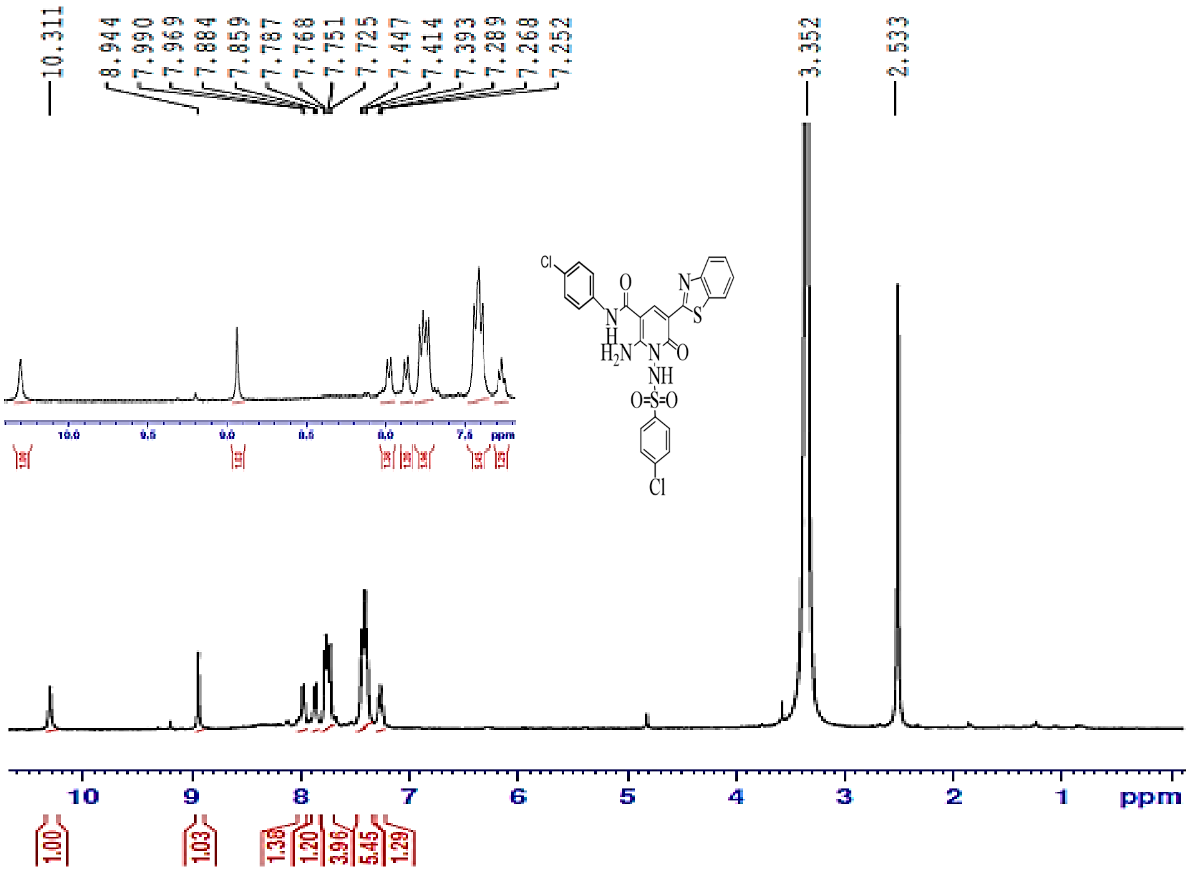


**Figure S4.** $^{1}$H NMR spectrum of compound **6b.**

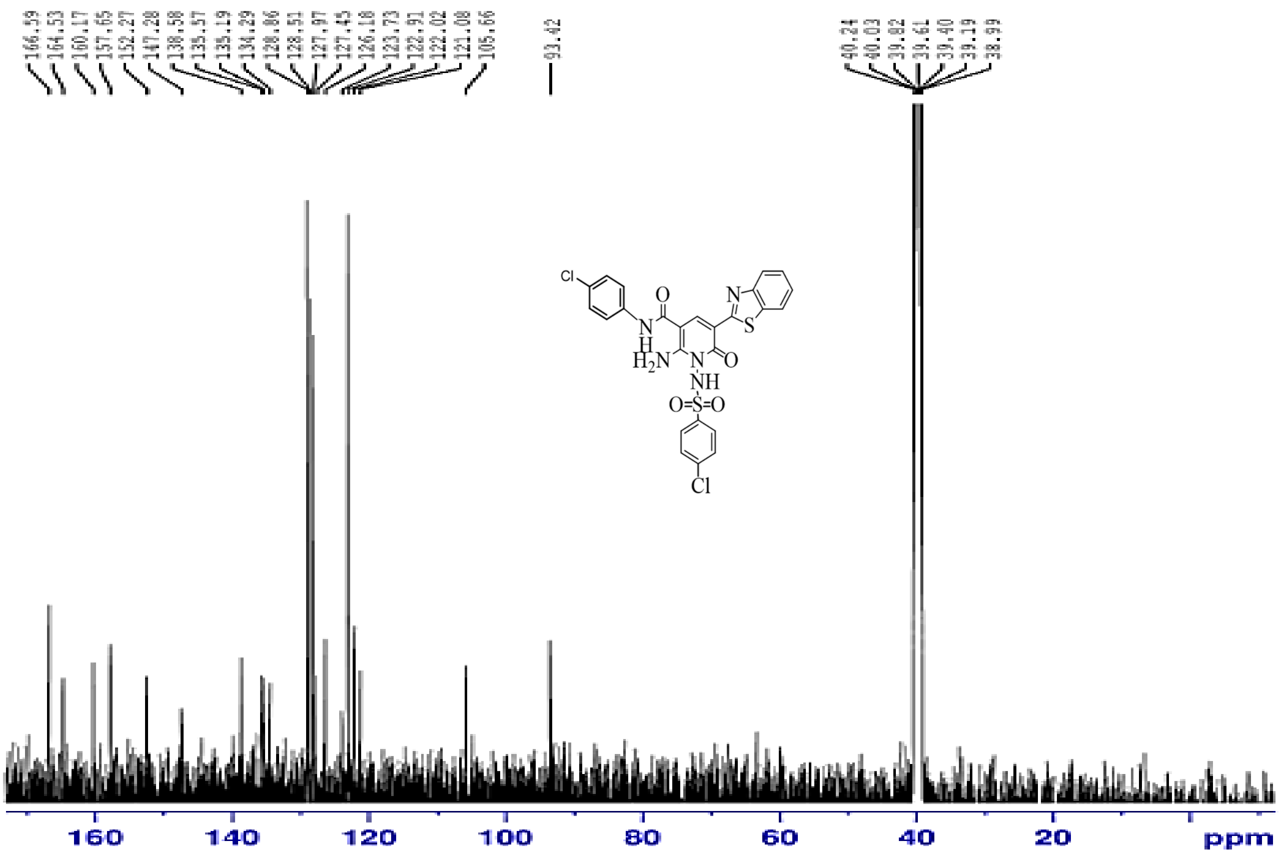


**Figure S5.** $^{13}$C NMR spectrum of compound **6b.**

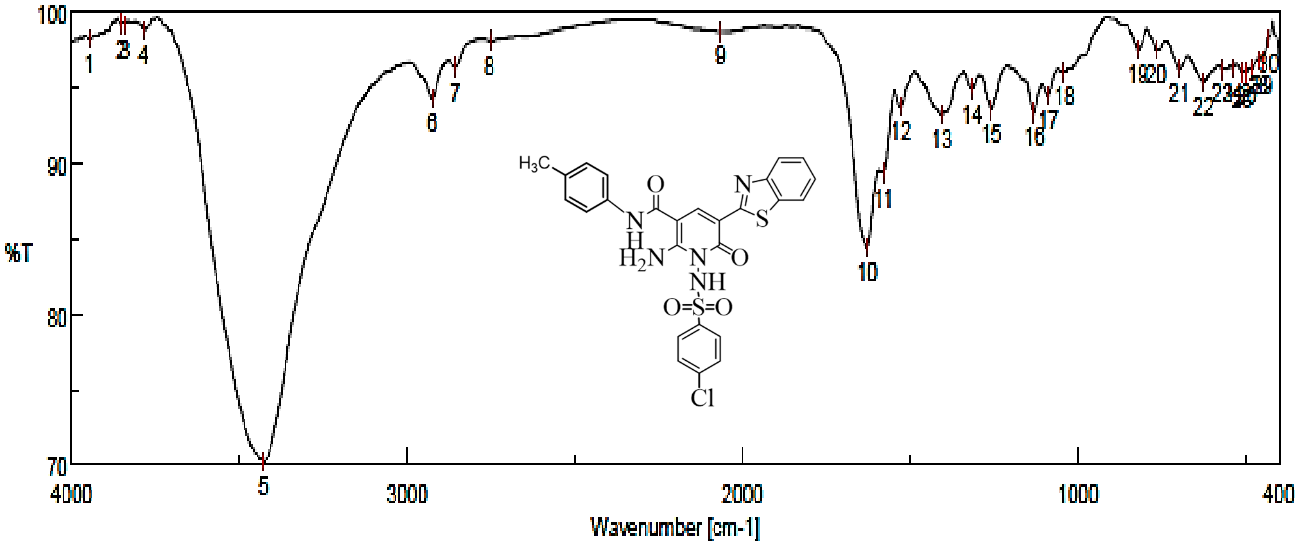


[ Result of Peak Picking ]

| No. | Position | Intensity | No. | Position | Intensity | No. | Position | Intensity | No. | Position | Intensity |
|---|---|---|---|---|---|---|---|---|---|---|---|
| 1 | 3949.5 | 98.2757 | 2 | 3854.04 | 99.2073 | 3 | 3841.51 | 99.1916 | 4 | 3784.62 | 98.8228 |
| 5 | 3427.85 | 70.2067 | 6 | 2925.48 | 94.2941 | 7 | 2856.06 | 96.3547 | 8 | 2753.85 | 98.1295 |
| 9 | 2065.39 | 98.7317 | 10 | 1628.59 | 84.4046 | 11 | 1579.41 | 89.3745 | 12 | 1528.31 | 93.7767 |
| 13 | 1405.85 | 93.2235 | 14 | 1315.21 | 94.9014 | 15 | 1258.32 | 93.5655 | 16 | 1130.08 | 93.3155 |
| 17 | 1088.62 | 94.4773 | 18 | 1041.37 | 96.0455 | 19 | 820.563 | 97.528 | 20 | 766.566 | 97.5141 |
| 21 | 698.105 | 96.2908 | 22 | 624.823 | 95.3856 | 23 | 569.862 | 96.2106 | 24 | 538.042 | 96.3025 |
| 25 | 510.08 | 96.011 | 26 | 499.473 | 96.0393 | 27 | 484.045 | 96.1977 | 28 | 459.939 | 96.8251 |
| 29 | 449.333 | 96.7578 | 30 | 431.012 | 98.2454 | | | | | | |

**Figure S6.** IR spectrum of compound **6c.**

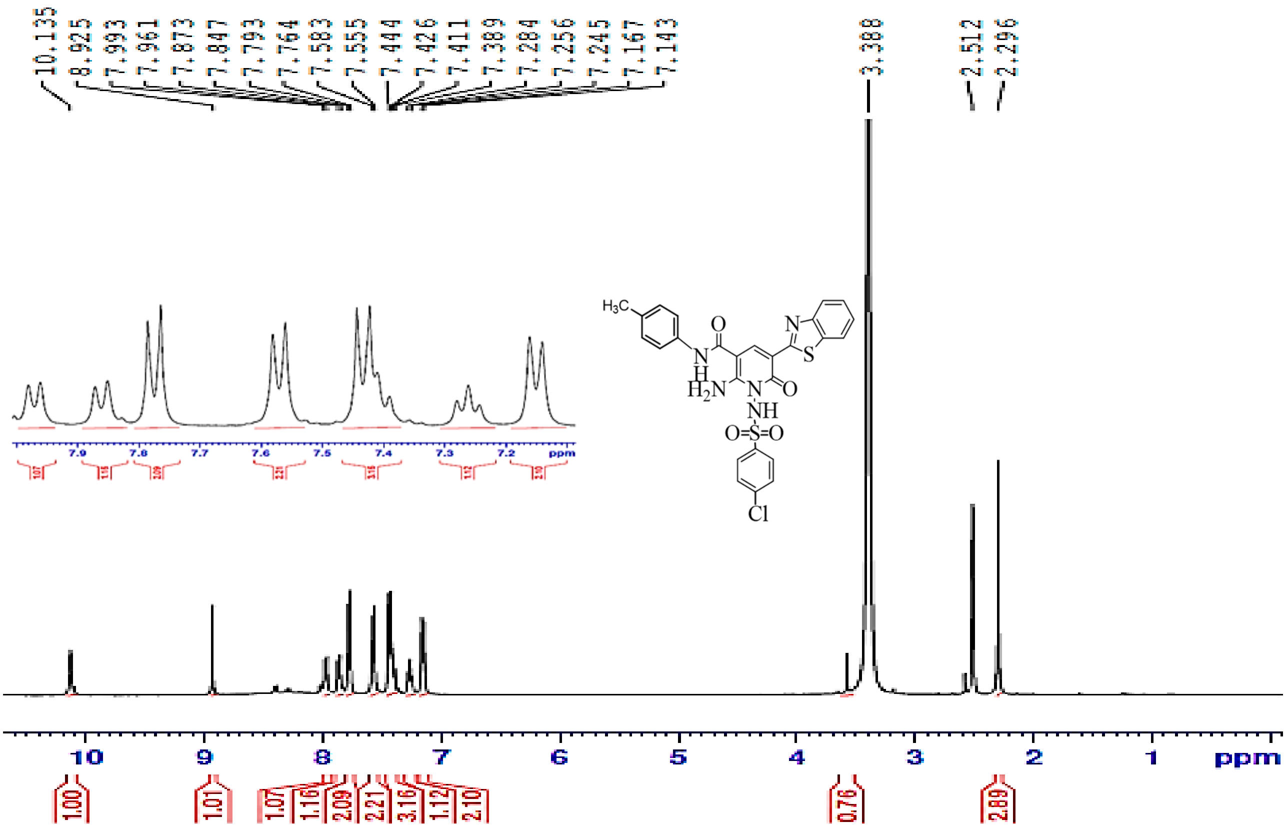

**Figure S7.** $^{1}$H NMR spectrum of compound **6c.**

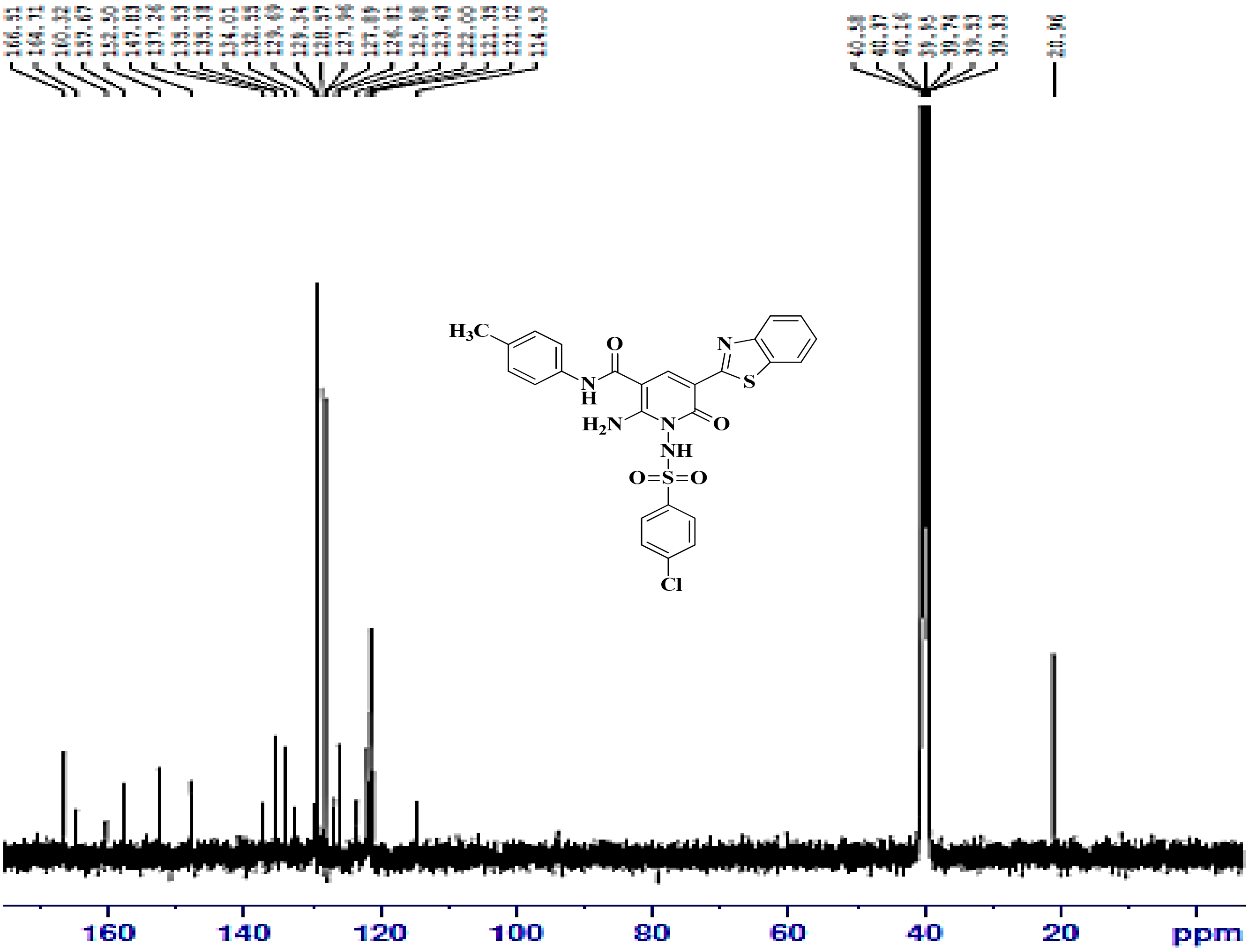


**Figure S8.** $^{13}$C NMR spectrum of compound **6c.**

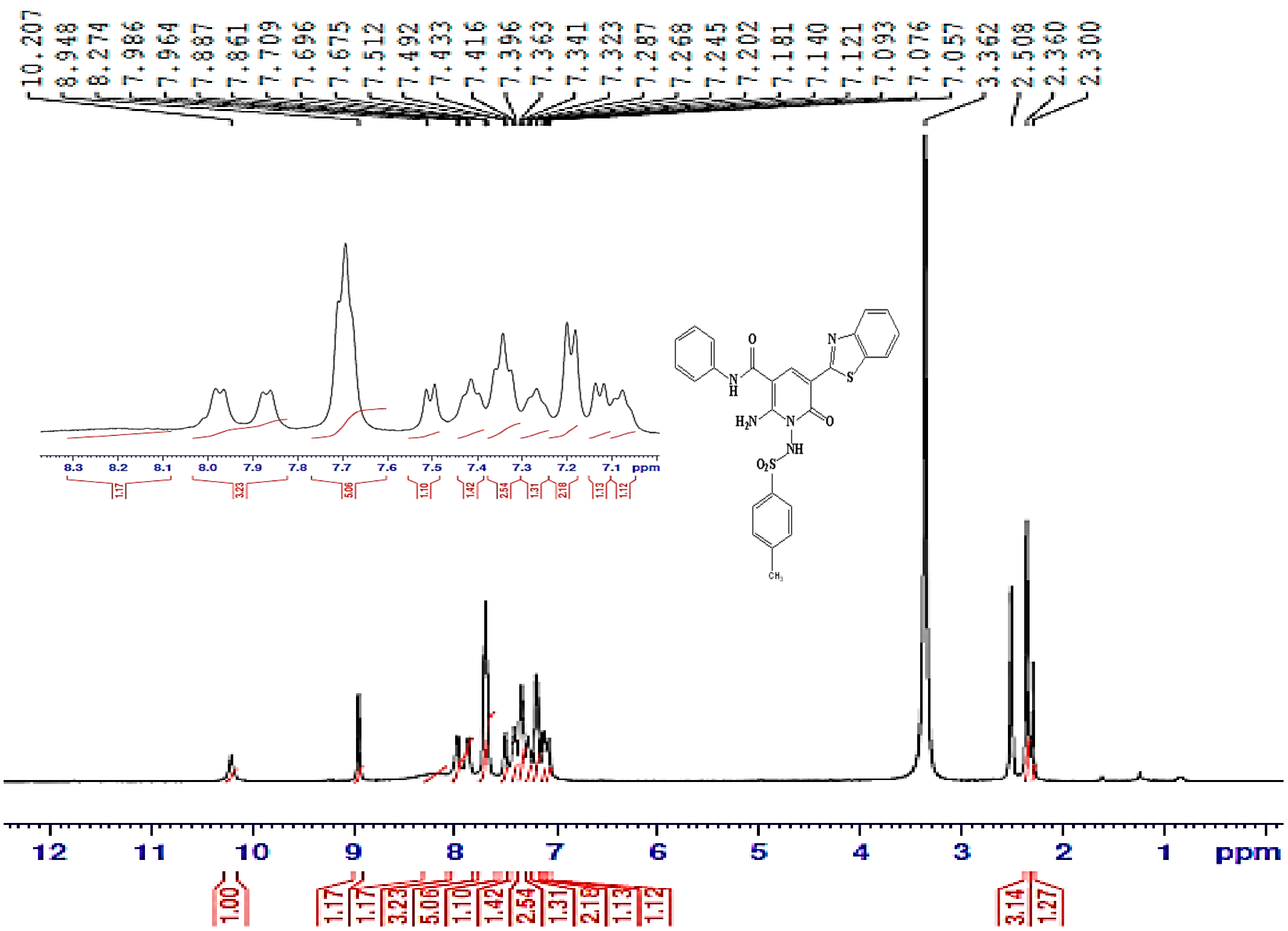

**Figure S9.** $^{1}$H NMR spectrum of compound **6d.**

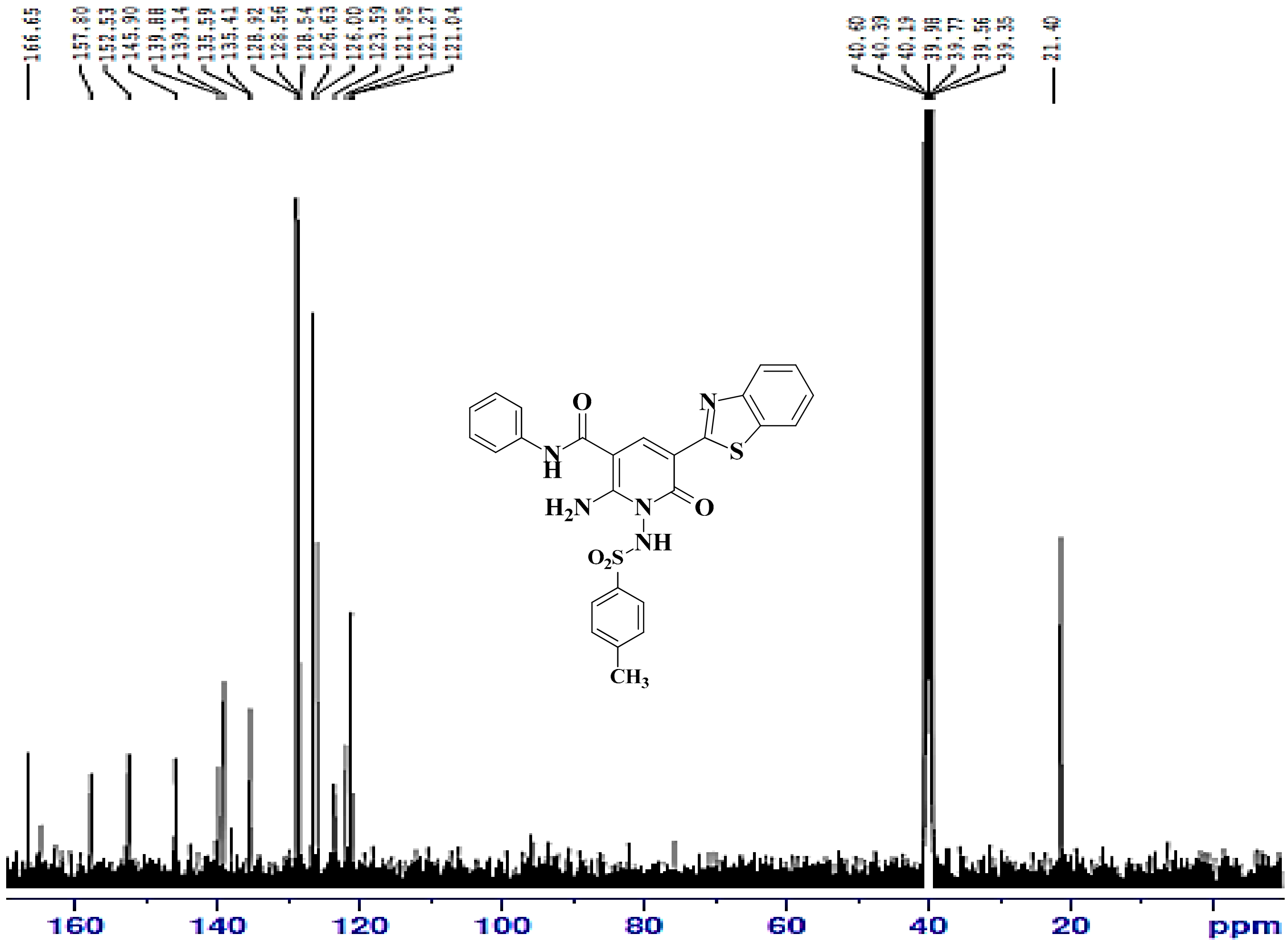

**Figure S10.** $^{13}C$ NMR spectrum of compound **6d.**

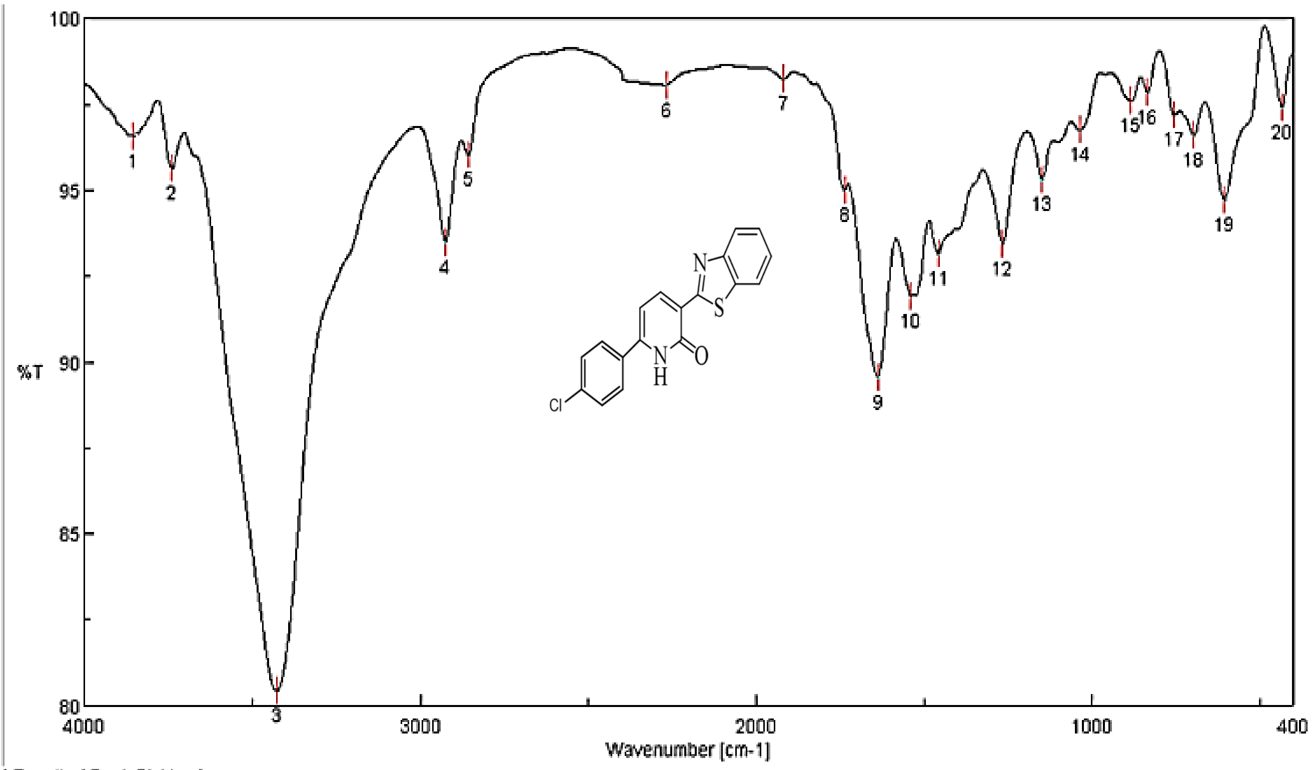


[ Result of Peak Picking ]

| No. | Position | Intensity | No. | Position | Intensity | No. | Position | Intensity | No. | Position | Intensity |
|---|---|---|---|---|---|---|---|---|---|---|---|
| 1 | 3854.04 | 96.56557 | 2 | 3739.3 | 95.62312 | 3 | 3427.85 | 80.41946 | 4 | 2924.52 | 93.47816 |
| 5 | 2857.02 | 96.01069 | 6 | 2267.88 | 98.04796 | 7 | 1920.75 | 98.22923 | 8 | 1736.58 | 95.00515 |
| 9 | 1637.27 | 89.53745 | 10 | 1539.88 | 91.89730 | 11 | 1456.96 | 93.12766 | 12 | 1267 | 93.42825 |
| 13 | 1149.37 | 95.28928 | 14 | 1035.59 | 96.74112 | 15 | 885.166 | 97.58877 | 16 | 835.026 | 97.82766 |
| 17 | 756.923 | 97.17838 | 18 | 697.141 | 96.60280 | 19 | 606.503 | 94.71815 | 20 | 433.905 | 97.40613 |

**Figure S11.** IR spectrum of compound **12a.**

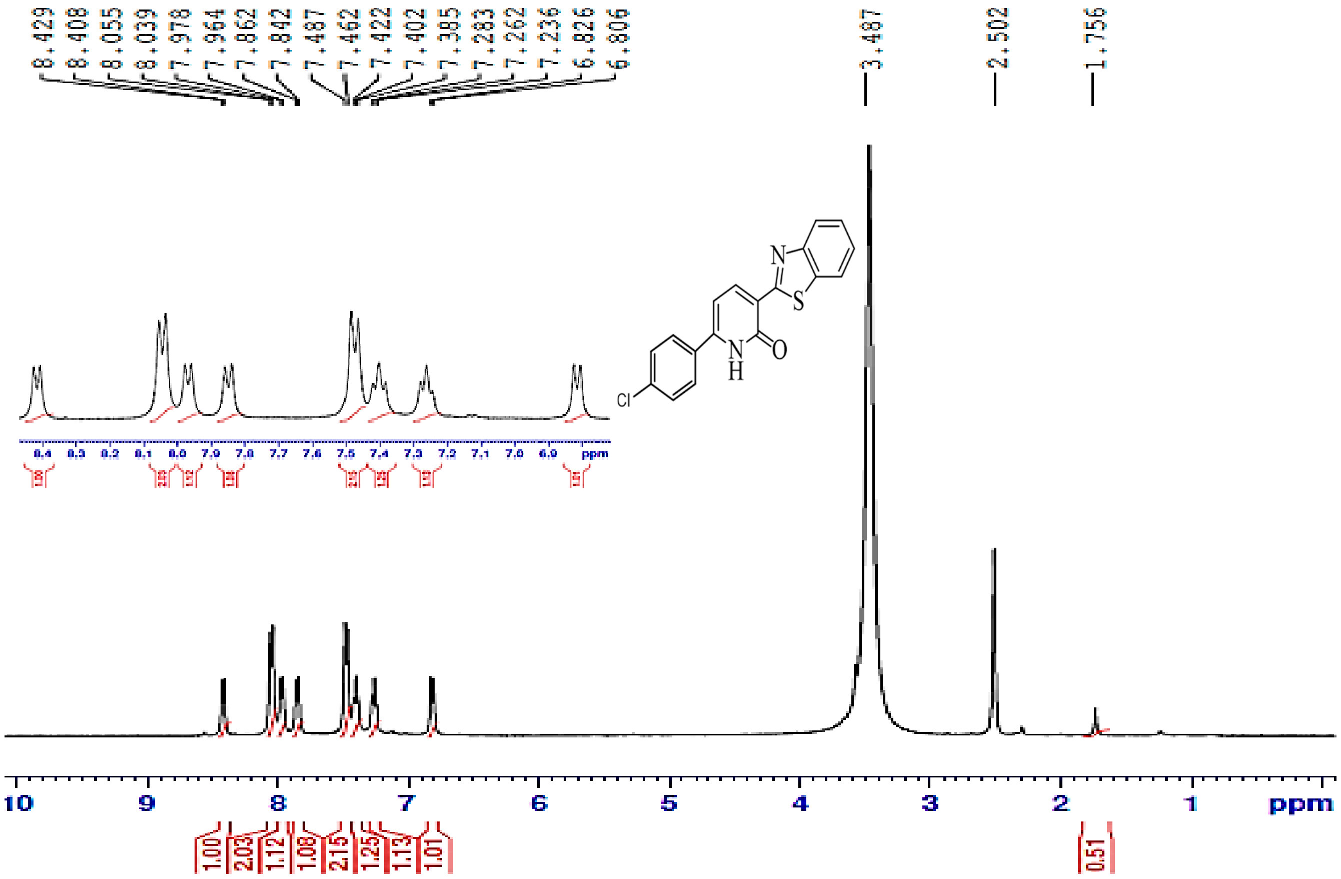


**Figure S12.** $^{1}$H NMR spectrum of compound **12a.**

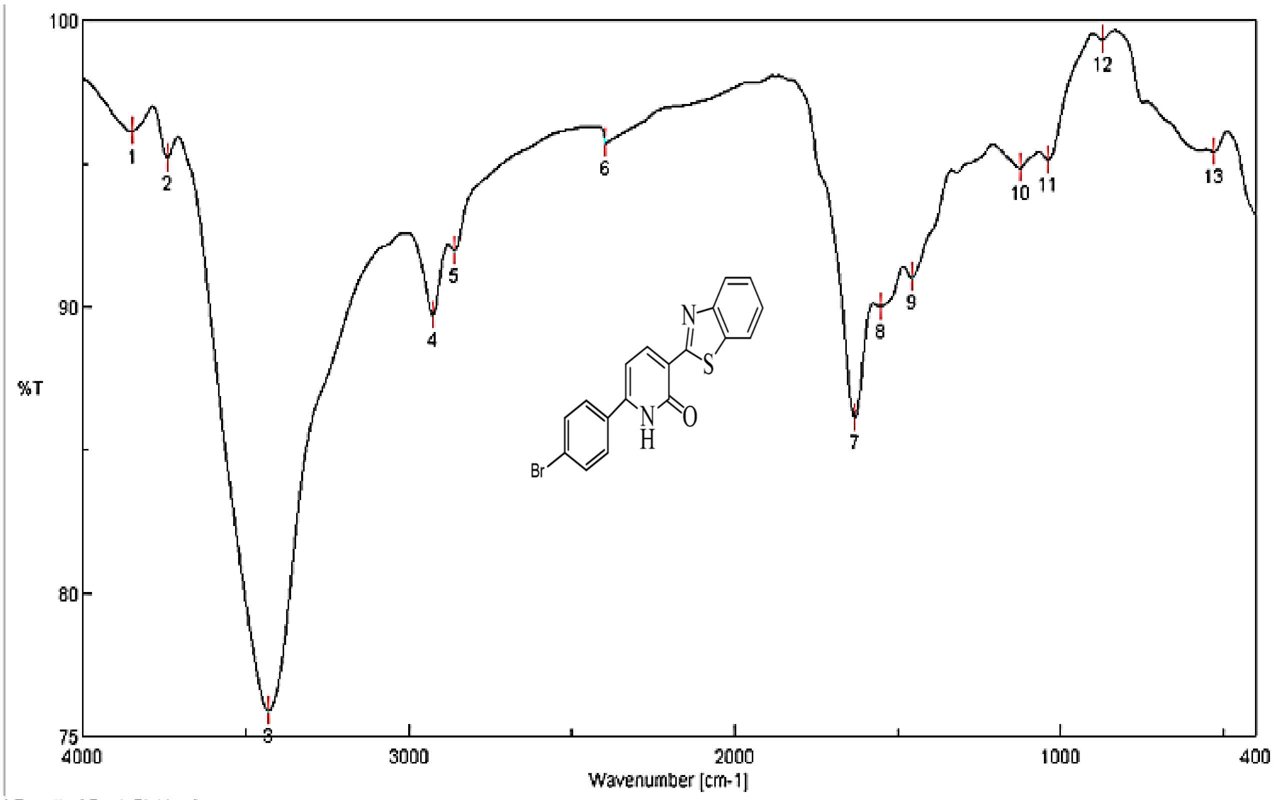


[ Result of Peak Picking ]

| No. | Position | Intensity | No. | Position | Intensity | No. | Position | Intensity | No. | Position | Intensity |
|---|---|---|---|---|---|---|---|---|---|---|---|
| 1 | 3848.26 | 96.12367 | 2 | 3741.23 | 95.19695 | 3 | 3429.78 | 75.88064 | 4 | 2926.45 | 89.69216 |
| 5 | 2859.92 | 91.94700 | 6 | 2396.12 | 95.71843 | 7 | 1631.48 | 86.11700 | 8 | 1551.45 | 90.00430 |
| 9 | 1457.92 | 91.02144 | 10 | 1125.26 | 94.83981 | 11 | 1038.48 | 95.12334 | 12 | 872.631 | 99.33387 |
| 13 | 530.328 | 95.41121 | | | | | | | | | |

**Figure S13.** IR spectrum of compound **12b.**

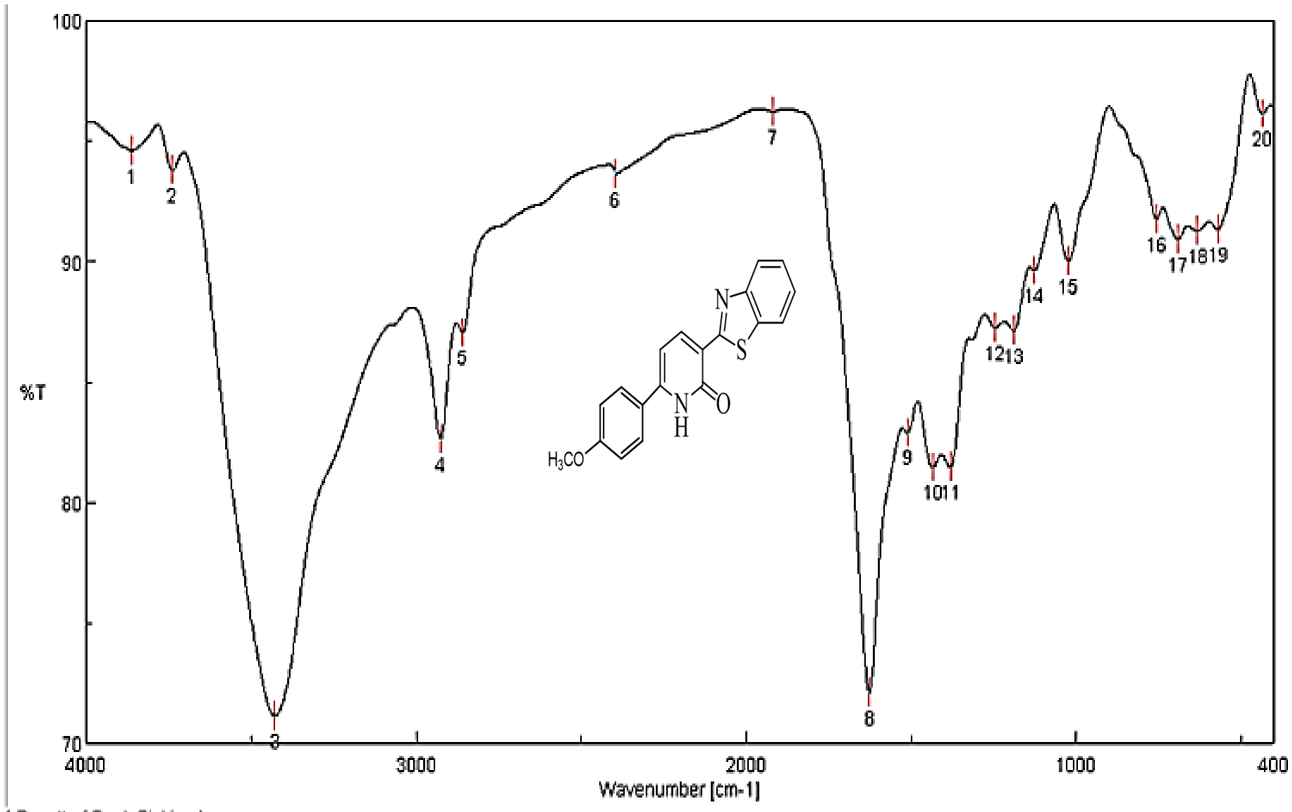


[ Result of Peak Picking ]

| No. | Position | Intensity | No. | Position | Intensity | No. | Position | Intensity | No. | Position | Intensity |
|---|---|---|---|---|---|---|---|---|---|---|---|
| 1 | 3861.76 | 94.60095 | 2 | 3739.3 | 93.78472 | 3 | 3428.81 | 71.13947 | 4 | 2926.45 | 82.61330 |
| 5 | 2859.92 | 87.04115 | 6 | 2396.12 | 93.60735 | 7 | 1918.82 | 96.23874 | 8 | 1626.66 | 72.08687 |
| 9 | 1510.95 | 82.88138 | 10 | 1434.78 | 81.45067 | 11 | 1380.78 | 81.45471 | 12 | 1244.83 | 87.24163 |
| 13 | 1189.86 | 87.12103 | 14 | 1127.19 | 89.62278 | 15 | 1023.05 | 90.03873 | 16 | 755.959 | 91.75848 |
| 17 | 691.355 | 90.92812 | 18 | 631.573 | 91.27695 | 19 | 570.826 | 91.33160 | 20 | 434.869 | 96.12226 |

**Figure S14.** IR spectrum of compound **12c.**

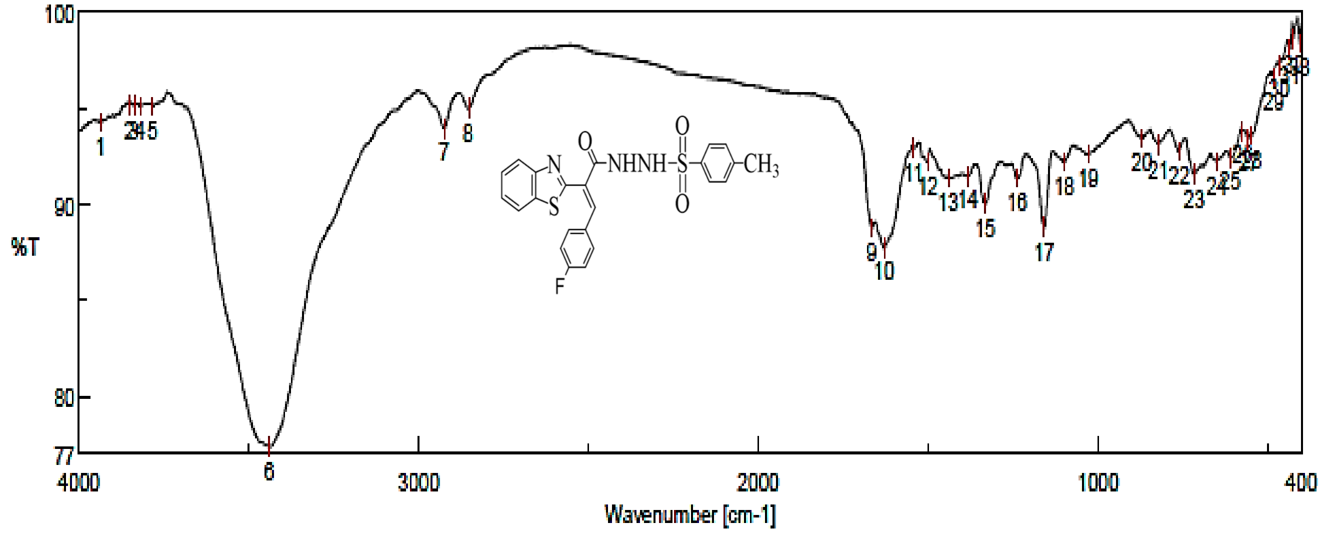


[ Result of Peak Picking ]

| No. | Position | Intensity | No. | Position | Intensity | No. | Position | Intensity | No. | Position | Intensity |
|---|---|---|---|---|---|---|---|---|---|---|---|
| 1 | 3936.97 | 94.2354 | 2 | 3851.15 | 95.1289 | 3 | 3837.65 | 95.1316 | 4 | 3821.26 | 95.1003 |
| 5 | 3782.69 | 95.0827 | 6 | 3440.39 | 77.3338 | 7 | 2925.48 | 93.9391 | 8 | 2854.13 | 94.9799 |
| 9 | 1665.23 | 88.7538 | 10 | 1629.55 | 87.7054 | 11 | 1542.77 | 92.9358 | 12 | 1502.28 | 92.1589 |
| 13 | 1440.56 | 91.3436 | 14 | 1383.68 | 91.4521 | 15 | 1332.57 | 90.0381 | 16 | 1238.08 | 91.3208 |
| 17 | 1159.01 | 88.7778 | 18 | 1099.23 | 92.1586 | 19 | 1028.84 | 92.5648 | 20 | 869.739 | 93.4382 |
| 21 | 822.491 | 93.0817 | 22 | 761.744 | 92.7445 | 23 | 716.425 | 91.5578 | 24 | 648.929 | 92.2198 |
| 25 | 610.36 | 92.412 | 26 | 573.719 | 93.7649 | 27 | 558.291 | 93.3928 | 28 | 547.685 | 93.5558 |
| 29 | 484.045 | 96.6387 | 30 | 465.725 | 97.2193 | 31 | 434.869 | 98.0364 | 32 | 423.298 | 98.6045 |
| 33 | 405.942 | 98.3875 | | | | | | | | | |

**Figure S15.** IR spectrum of compound **14a.**

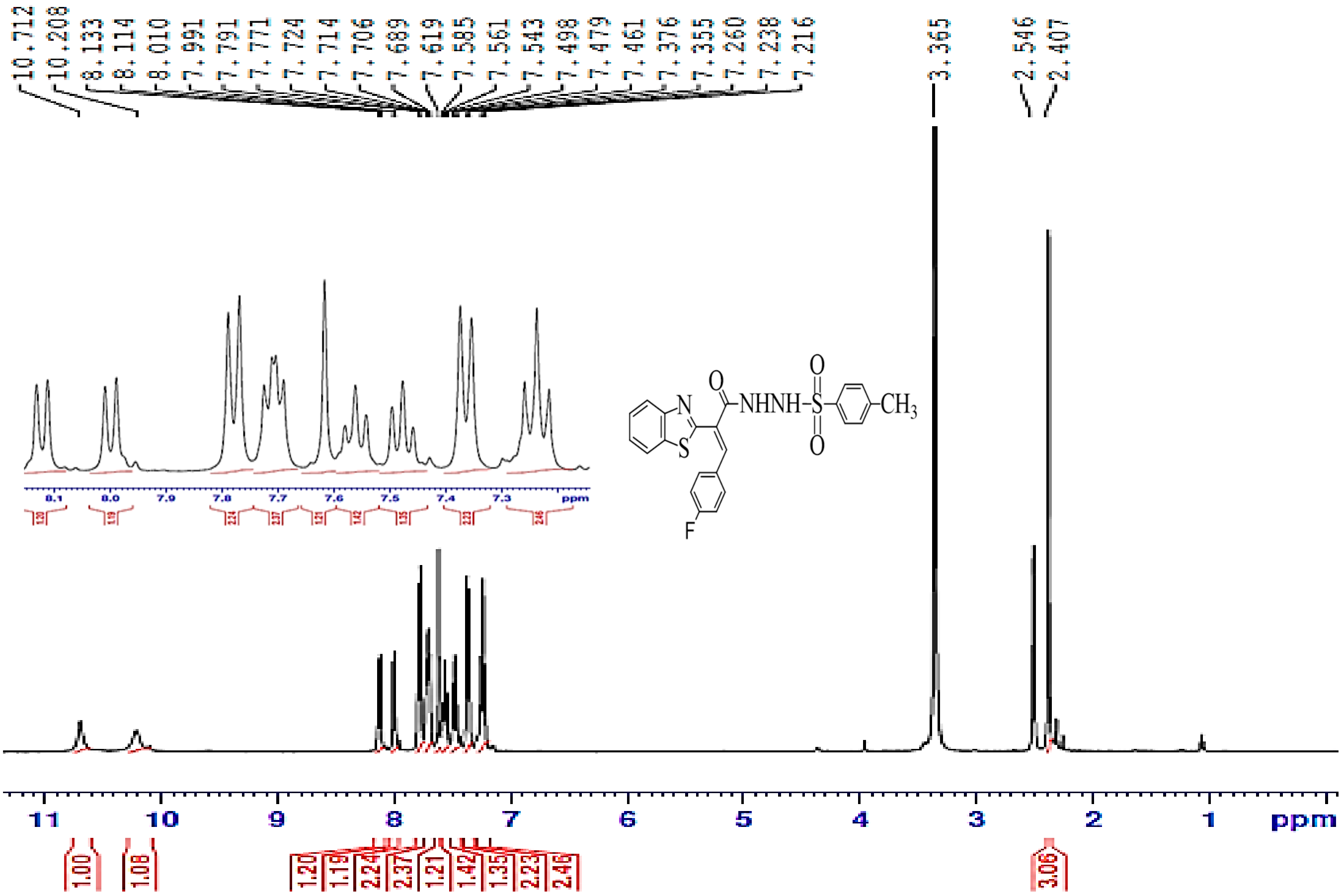

**Figure 16.** $^{1}$H NMR spectrum of compound **14a.**

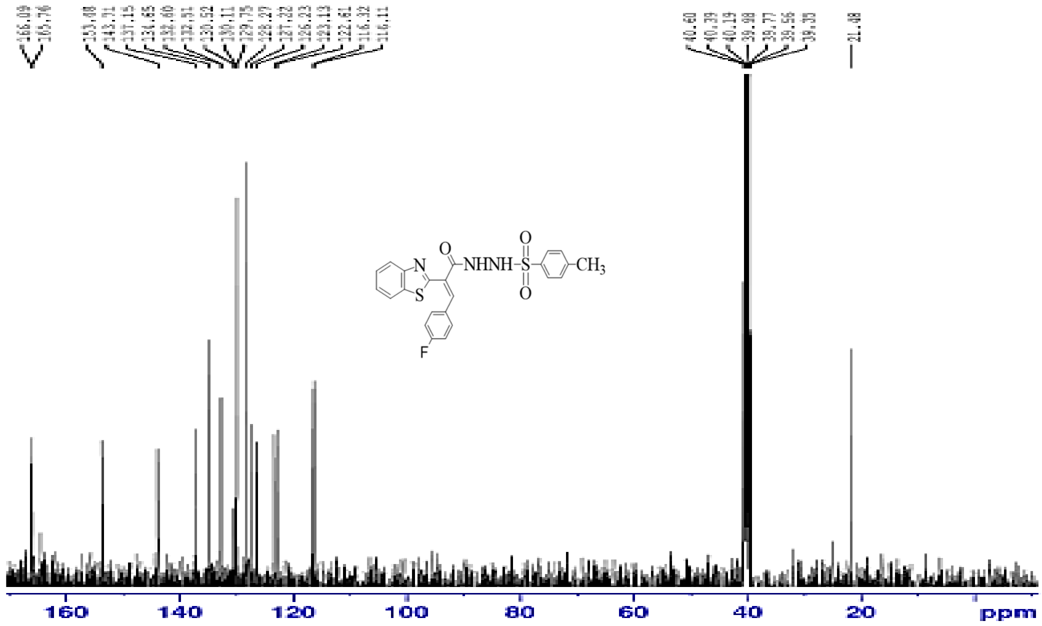

**Figure S17.** $^{13}$C NMR spectrum of compound 14a.

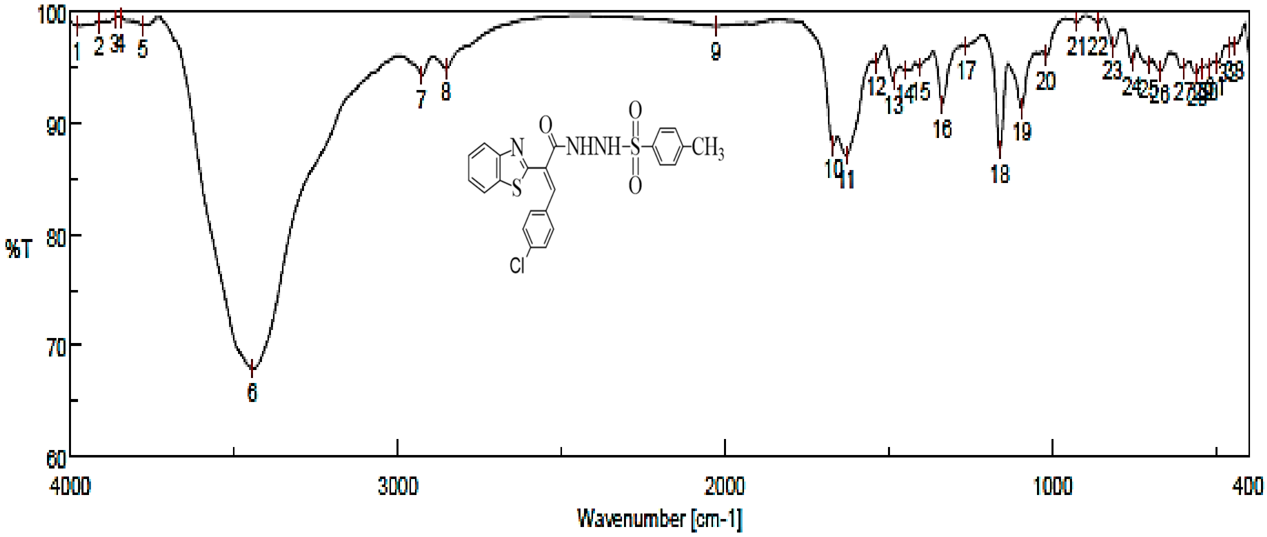


[ Result of Peak Picking ]

| No. | Position | Intensity | No. | Position | Intensity | No. | Position | Intensity | No. | Position | Intensity |
|---|---|---|---|---|---|---|---|---|---|---|---|
| 1 | 3981.32 | 98.7561 | 2 | 3912.86 | 99.0324 | 3 | 3864.65 | 99.3495 | 4 | 3848.26 | 99.4584 |
| 5 | 3778.83 | 98.8036 | 6 | 3444.24 | 67.936 | 7 | 2926.45 | 94.3296 | 8 | 2852.2 | 94.9272 |
| 9 | 2027.78 | 98.7526 | 10 | 1669.09 | 87.9933 | 11 | 1630.52 | 87.0754 | 12 | 1538.92 | 95.4769 |
| 13 | 1485.88 | 93.8559 | 14 | 1451.17 | 94.7763 | 15 | 1404.89 | 95.0532 | 16 | 1336.43 | 91.7402 |
| 17 | 1264.11 | 96.9563 | 18 | 1160.94 | 87.5901 | 19 | 1094.4 | 91.328 | 20 | 1019.19 | 96.094 |
| 21 | 925.664 | 99.1424 | 22 | 881.06 | 99.0959 | 23 | 815.742 | 96.8537 | 24 | 755.959 | 95.5077 |
| 25 | 705.819 | 95.2998 | 26 | 673.035 | 94.7105 | 27 | 598.789 | 94.9346 | 28 | 557.327 | 94.5036 |
| 29 | 539.971 | 94.9752 | 30 | 522.615 | 95.0049 | 31 | 501.401 | 95.4607 | 32 | 461.868 | 96.8266 |
| 33 | 444.512 | 96.9759 | | | | | | | | | |

**Figure S18.** IR spectrum of compound **14b.**

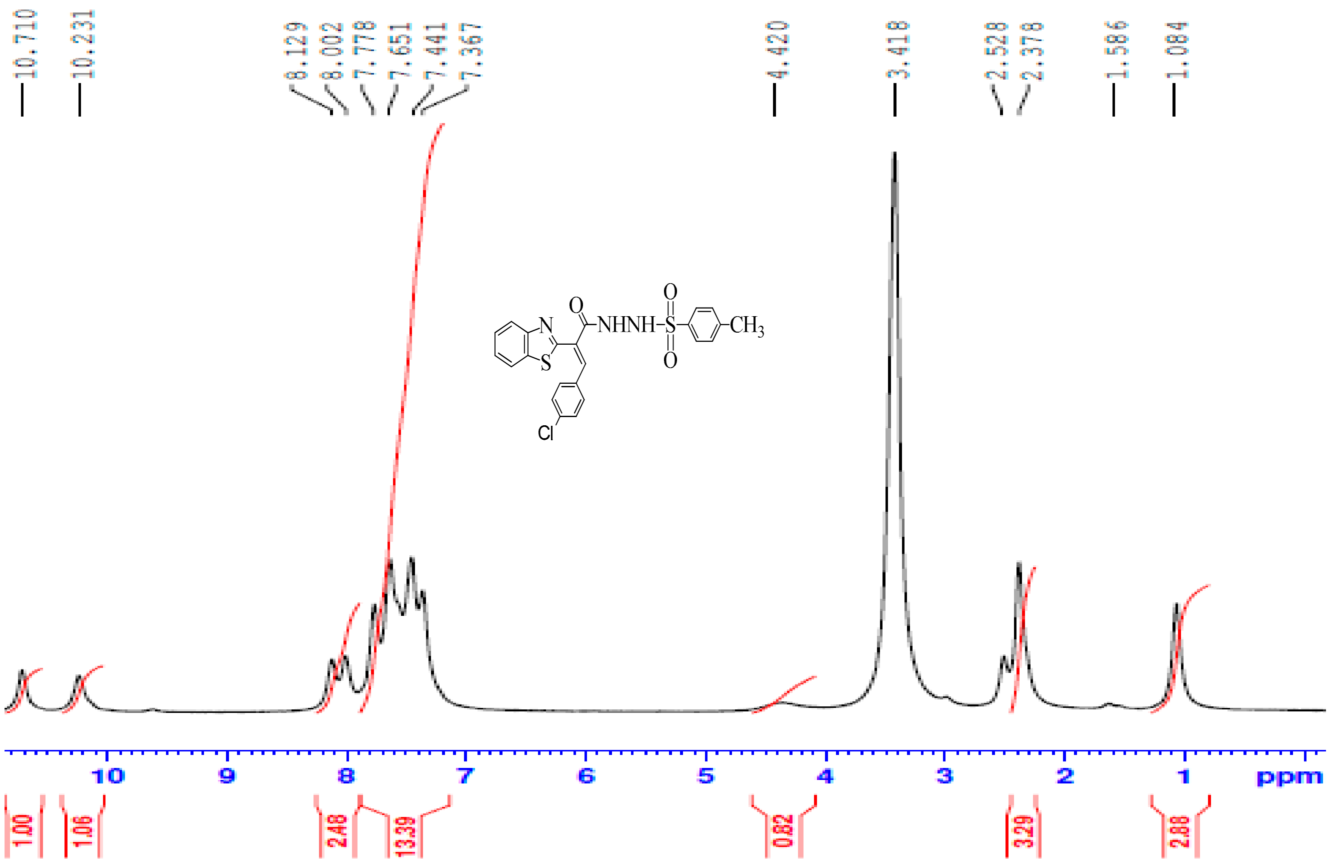


**Figure S19.** ¹H NMR spectrum of compound **14b.**

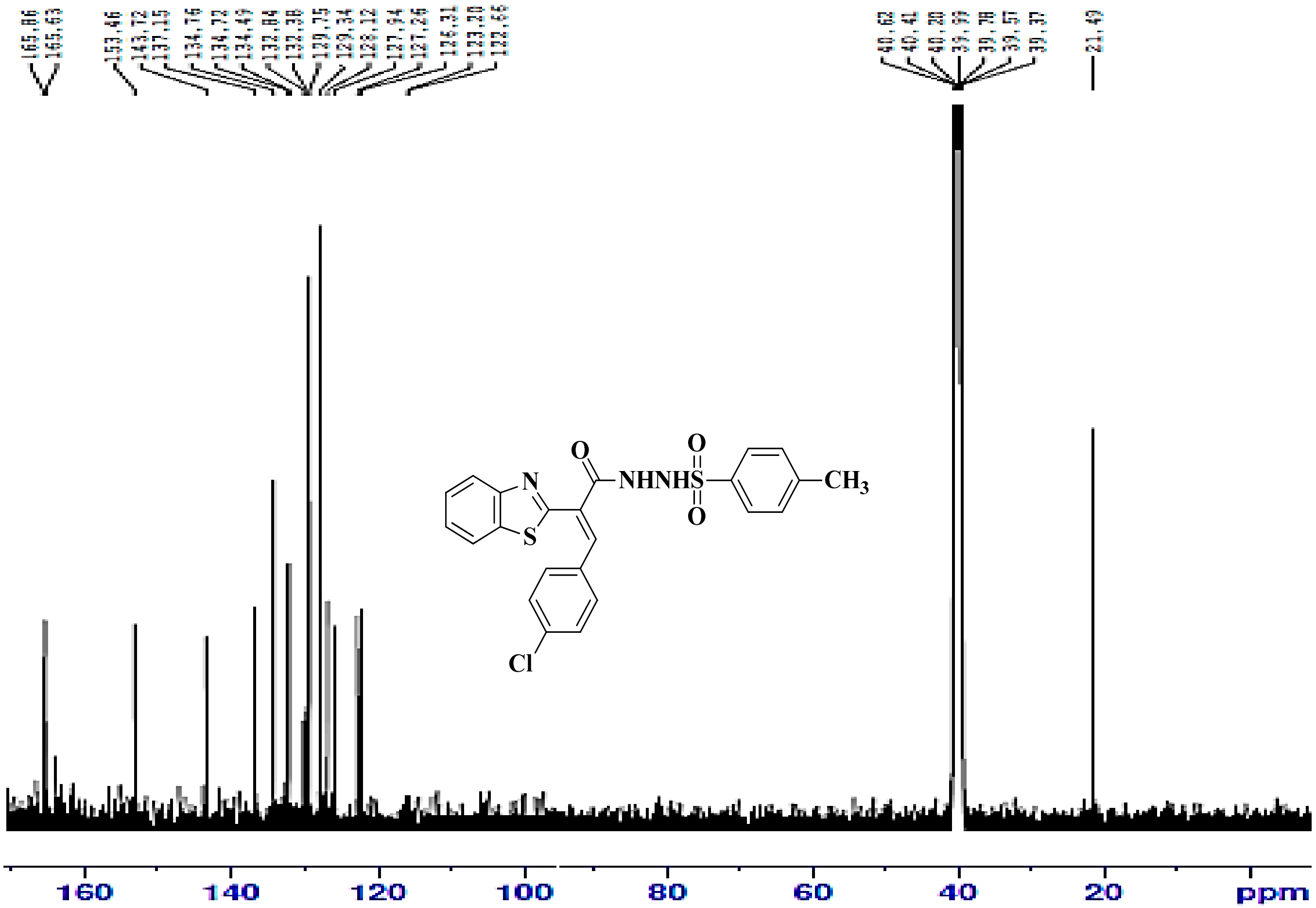

**Figure S20.** 13C NMR spectrum of compound 14b.

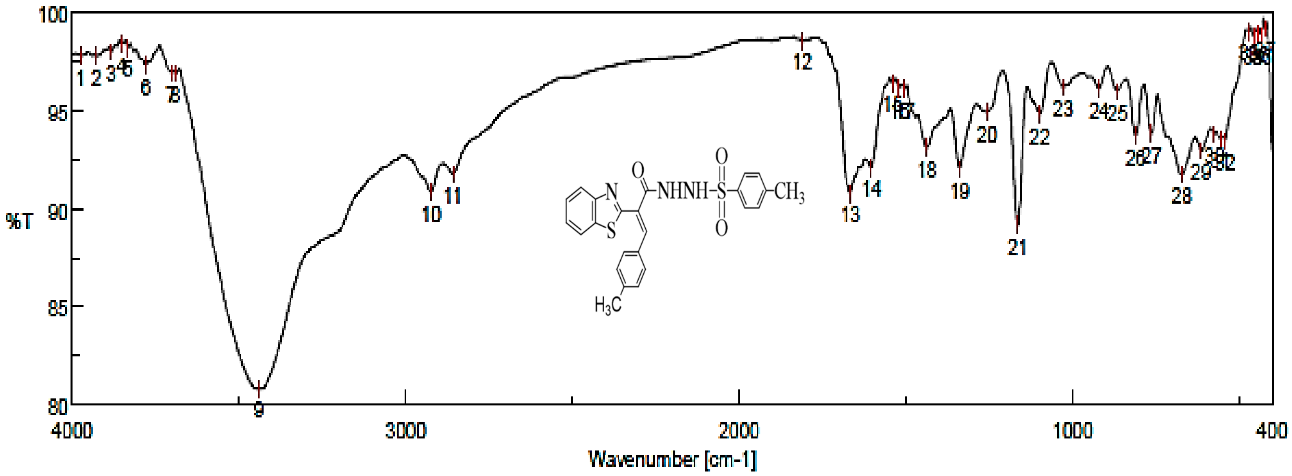


[ Result of Peak Picking ]

| No. | Position | Intensity | No. | Position | Intensity | No. | Position | Intensity | No. | Position | Intensity |
|---|---|---|---|---|---|---|---|---|---|---|---|
| 1 | 3976.5 | 97.8136 | 2 | 3929.25 | 97.7964 | 3 | 3883.93 | 98.0179 | 4 | 3851.15 | 98.3982 |
| 5 | 3833.79 | 98.1948 | 6 | 3780.76 | 97.3311 | 7 | 3702.66 | 96.9306 | 8 | 3691.09 | 96.9473 |
| 9 | 3439.42 | 80.8102 | 10 | 2925.48 | 90.8636 | 11 | 2858.95 | 91.7801 | 12 | 1810.83 | 98.5498 |
| 13 | 1666.2 | 90.7771 | 14 | 1603.52 | 92.0932 | 15 | 1538.92 | 96.4053 | 16 | 1519.63 | 96.1365 |
| 17 | 1503.24 | 96.1216 | 18 | 1436.71 | 93.1451 | 19 | 1338.36 | 92.0413 | 20 | 1256.4 | 94.9475 |
| 21 | 1163.83 | 89.1658 | 22 | 1097.3 | 94.8264 | 23 | 1027.87 | 96.2301 | 24 | 919.879 | 96.1412 |
| 25 | 864.917 | 96.0026 | 26 | 809.956 | 93.7349 | 27 | 762.709 | 93.8664 | 28 | 672.071 | 91.763 |
| 29 | 614.217 | 92.9742 | 30 | 573.719 | 93.8462 | 31 | 556.363 | 93.584 | 32 | 542.863 | 93.4212 |
| 33 | 469.582 | 99.0582 | 34 | 457.047 | 98.8314 | 35 | 441.619 | 98.9282 | 36 | 432.941 | 98.8293 |
| 37 | 421.37 | 99.2153 | | | | | | | | | |

**Figure S21.** IR spectrum of compound **14c.**

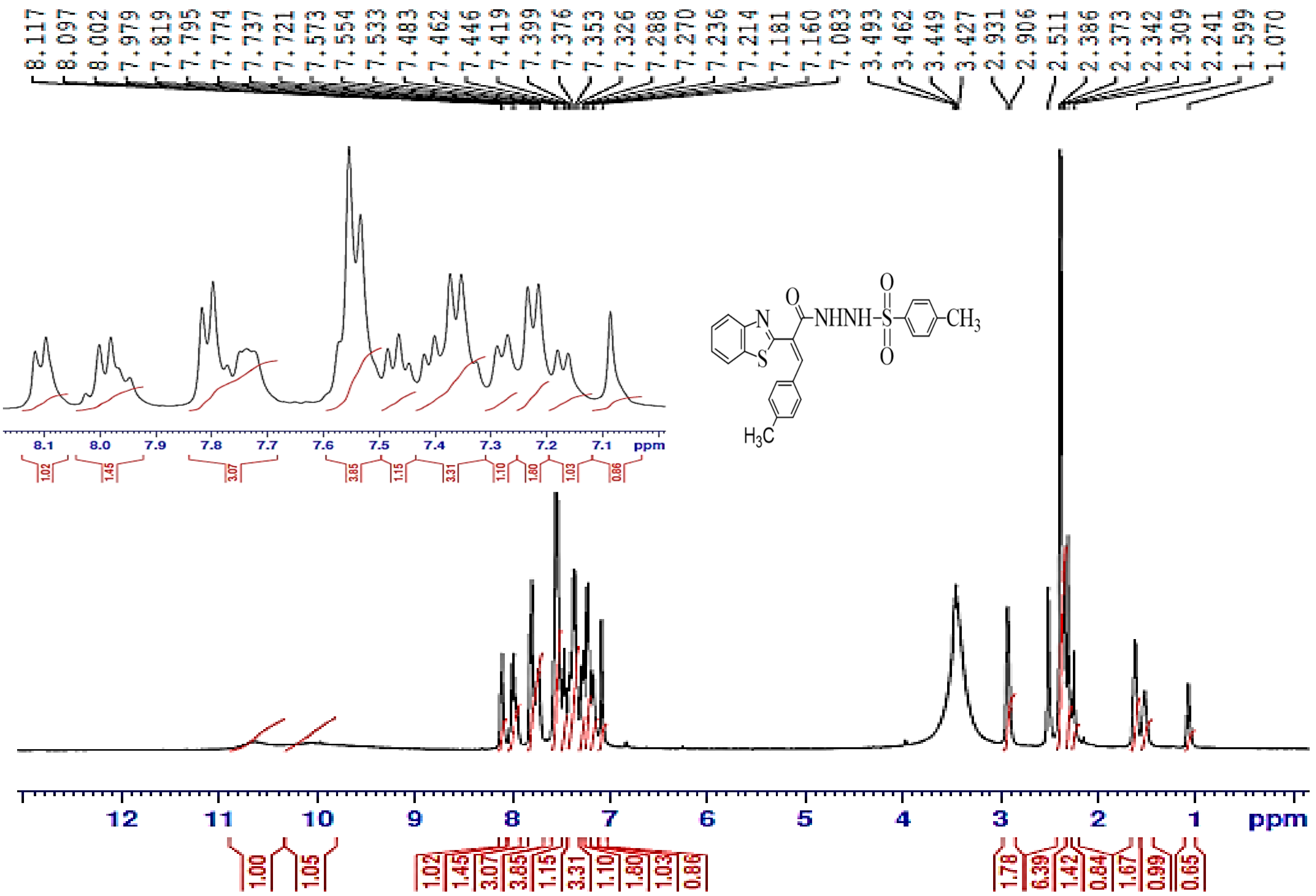

**Figure S22.** $^{1}$H NMR spectrum of compound **14c.**

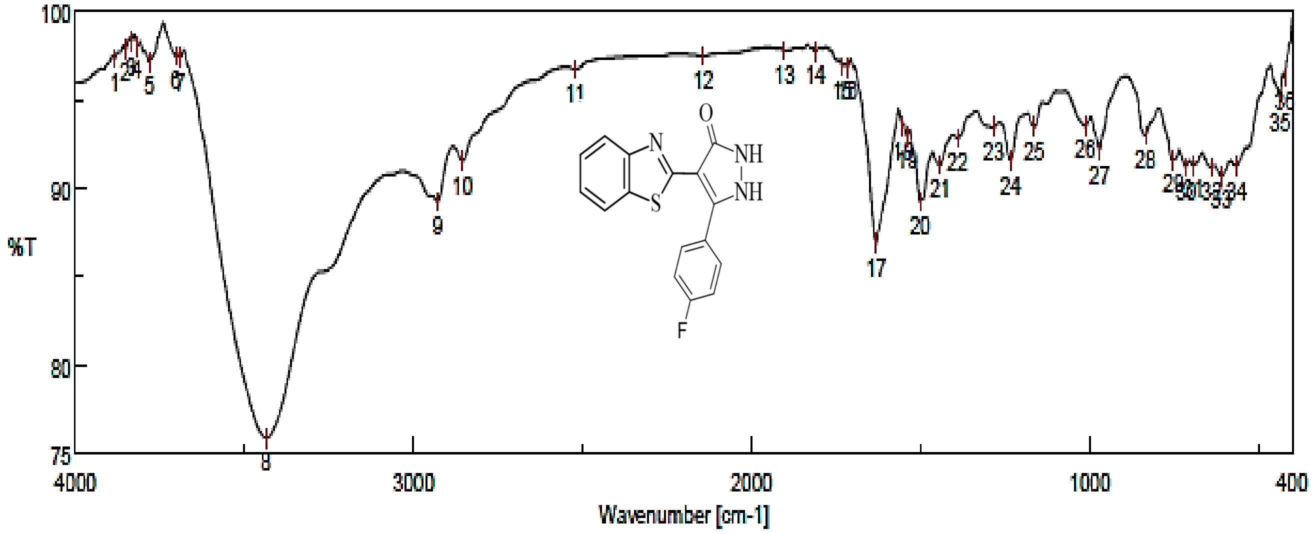


[ Result of Peak Picking ]

| No. | Position | Intensity | No. | Position | Intensity | No. | Position | Intensity | No. | Position | Intensity |
|---|---|---|---|---|---|---|---|---|---|---|---|
| 1 | 3883.93 | 97.3764 | 2 | 3853.08 | 97.9031 | 3 | 3835.72 | 98.3066 | 4 | 3817.4 | 98.0292 |
| 5 | 3781.72 | 97.1592 | 6 | 3703.62 | 97.4956 | 7 | 3690.12 | 97.4157 | 8 | 3437.49 | 75.8883 |
| 9 | 2929.34 | 89.2849 | 10 | 2856.06 | 91.5754 | 11 | 2521.47 | 96.7621 | 12 | 2143.49 | 97.524 |
| 13 | 1902.43 | 97.8137 | 14 | 1810.83 | 97.759 | 15 | 1730.8 | 96.9034 | 16 | 1717.3 | 96.8508 |
| 17 | 1631.48 | 86.8953 | 18 | 1553.38 | 93.5343 | 19 | 1538.92 | 92.9386 | 20 | 1501.31 | 89.2281 |
| 21 | 1442.49 | 91.3342 | 22 | 1390.42 | 92.8255 | 23 | 1284.36 | 93.4797 | 24 | 1234.22 | 91.5721 |
| 25 | 1163.83 | 93.4971 | 26 | 1013.41 | 93.5627 | 27 | 970.983 | 92.1122 | 28 | 835.026 | 93.0137 |
| 29 | 752.102 | 91.6026 | 30 | 714.497 | 91.2341 | 31 | 692.32 | 91.3207 | 32 | 639.287 | 91.2101 |
| 33 | 610.36 | 90.6767 | 34 | 564.077 | 91.2854 | 35 | 435.834 | 95.073 | 36 | 422.334 | 96.2819 |

**Figure S23.** IR spectrum of compound **16a.**

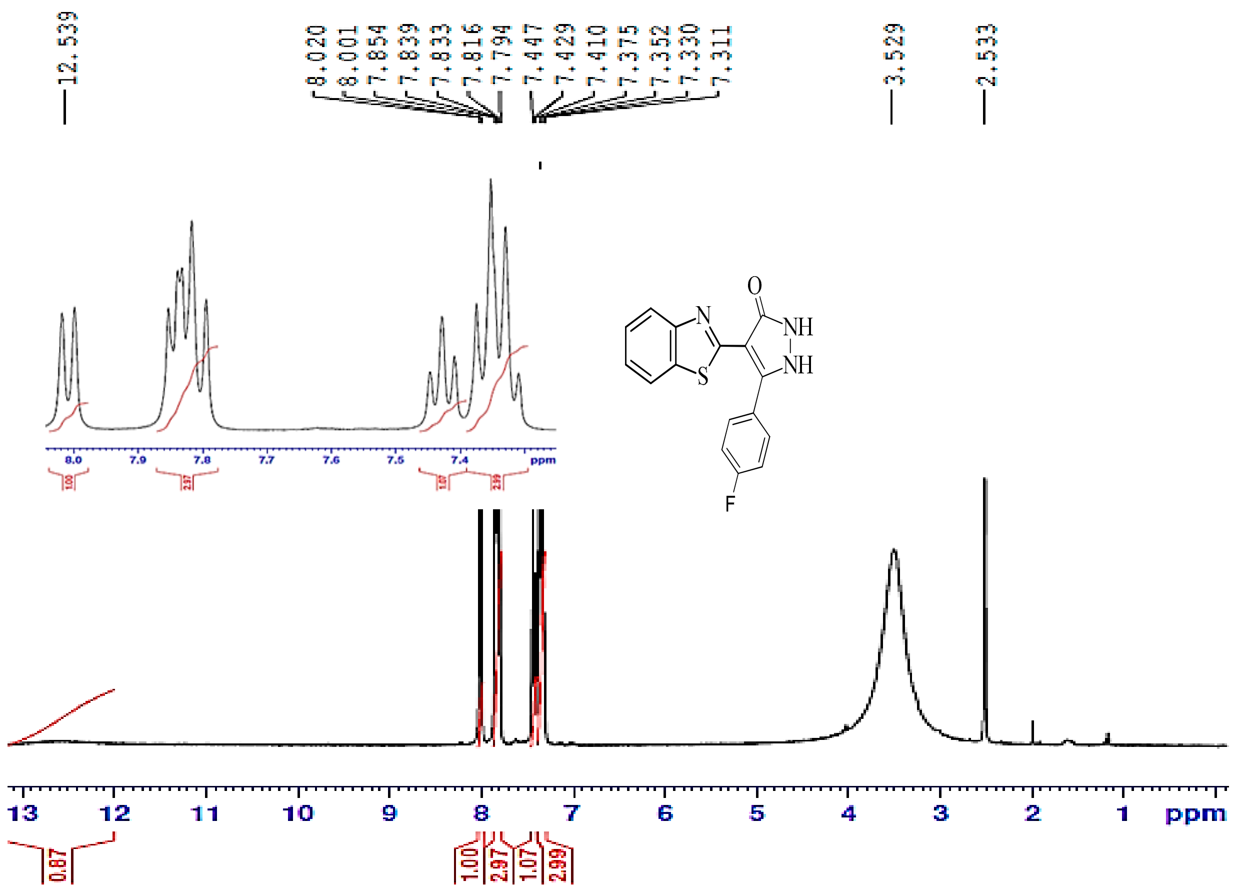


**Figure S24.** $^1$H NMR spectrum of compound **16a.**

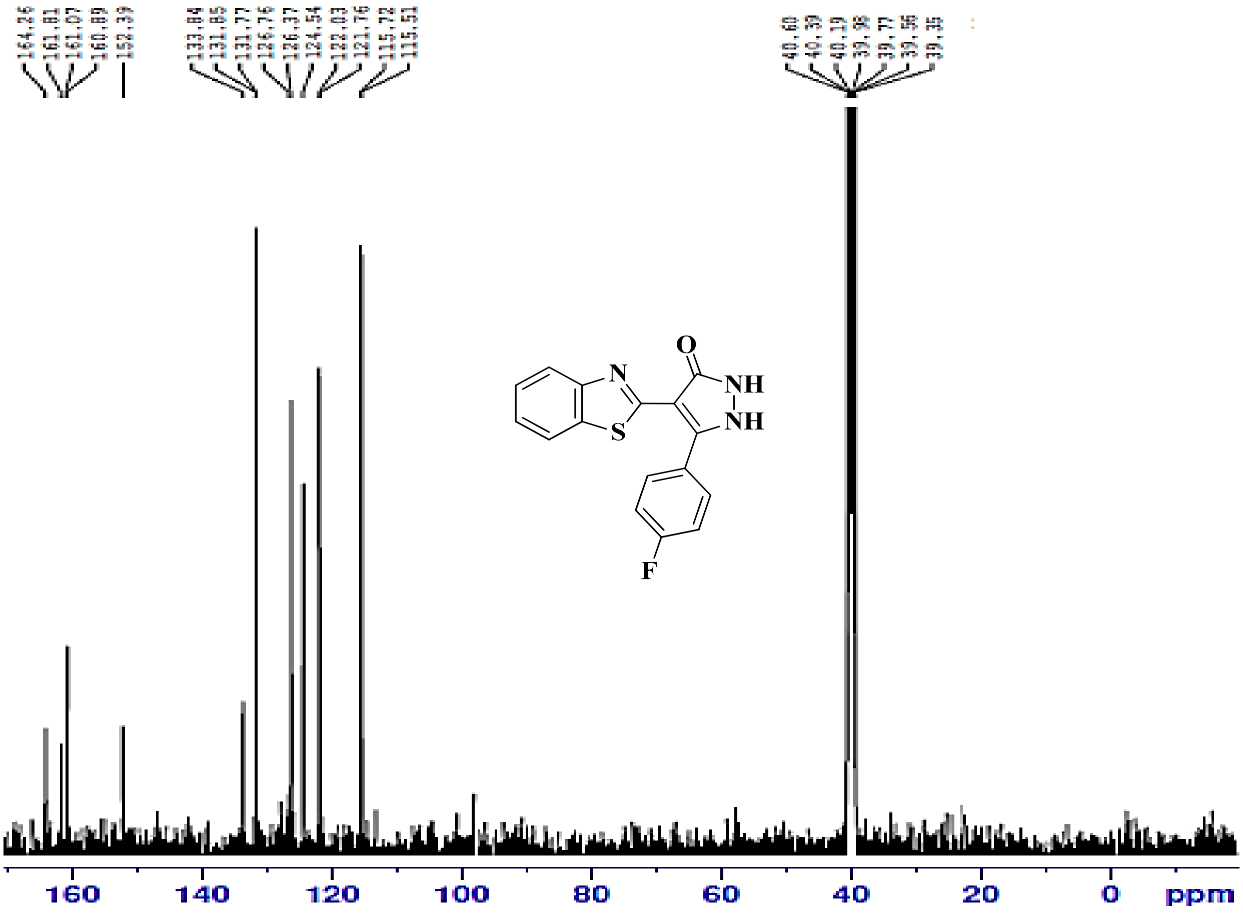


**Figure S25.** $^{13}$C NMR spectrum of compound **16a.**

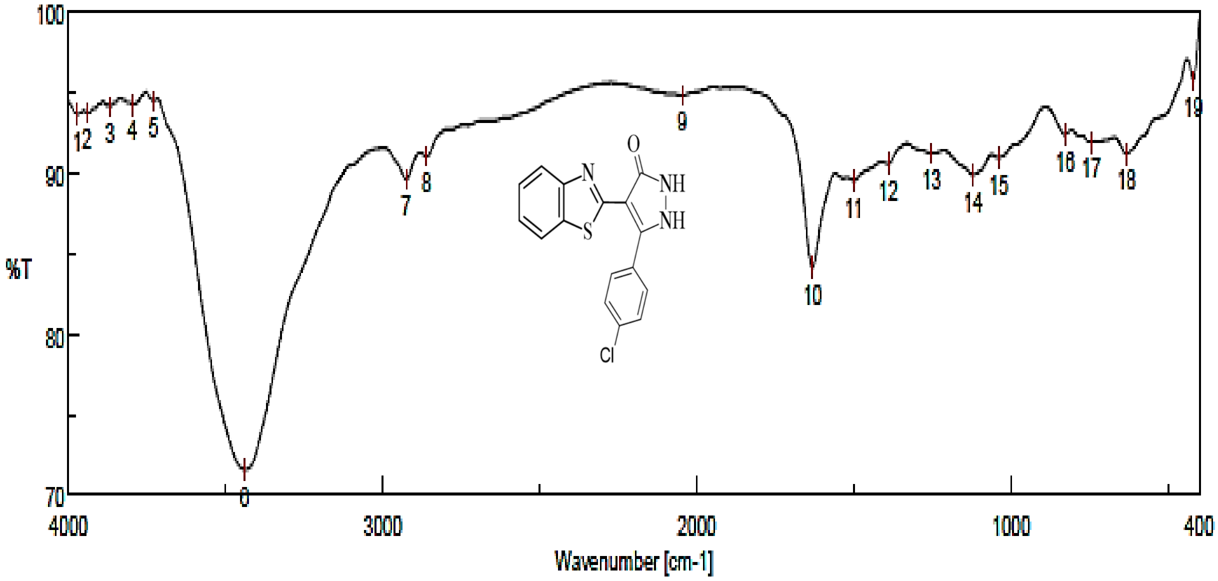


[ Result of Peak Picking ]

| No. | Position | Intensity | No. | Position | Intensity | No. | Position | Intensity | No. | Position | Intensity |
|---|---|---|---|---|---|---|---|---|---|---|---|
| 1 | 3972.64 | 93.6656 | 2 | 3941.79 | 93.8 | 3 | 3868.5 | 94.1651 | 4 | 3796.19 | 94.2498 |
| 5 | 3730.62 | 94.4921 | 6 | 3439.42 | 71.5424 | 7 | 2924.52 | 89.6097 | 8 | 2860.88 | 91.0454 |
| 9 | 2046.1 | 94.8677 | 10 | 1631.48 | 84.1206 | 11 | 1500.35 | 89.5484 | 12 | 1391.39 | 90.6189 |
| 13 | 1253.5 | 91.2511 | 14 | 1121.4 | 89.8957 | 15 | 1038.48 | 90.8876 | 16 | 828.277 | 92.3982 |
| 17 | 744.388 | 91.9156 | 18 | 631.573 | 91.2104 | 19 | 419.442 | 95.7847 | | | |

**Figure S26.** IR spectrum of compound **16b.**

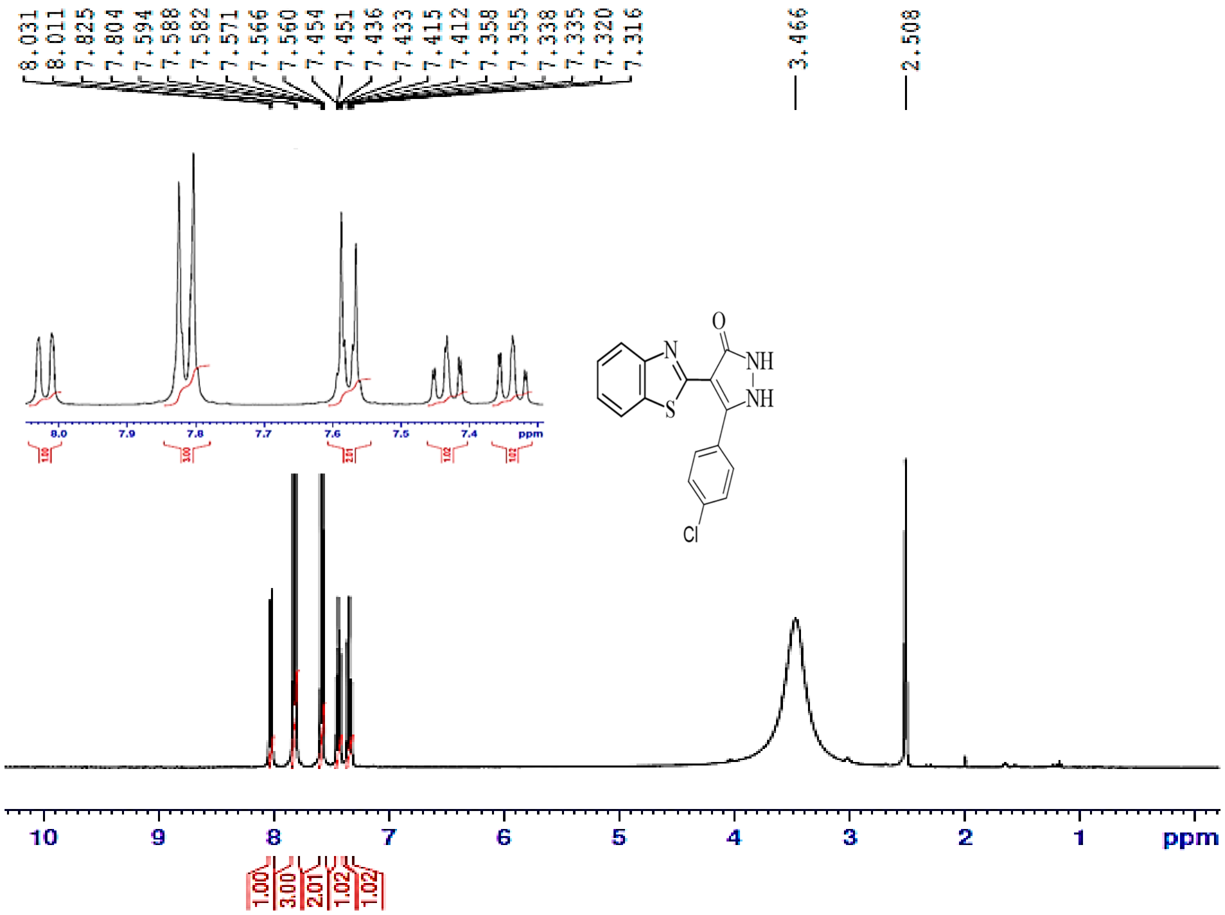


**Figure S27.** $^{1}$H NMR spectrum of compound **16b.**

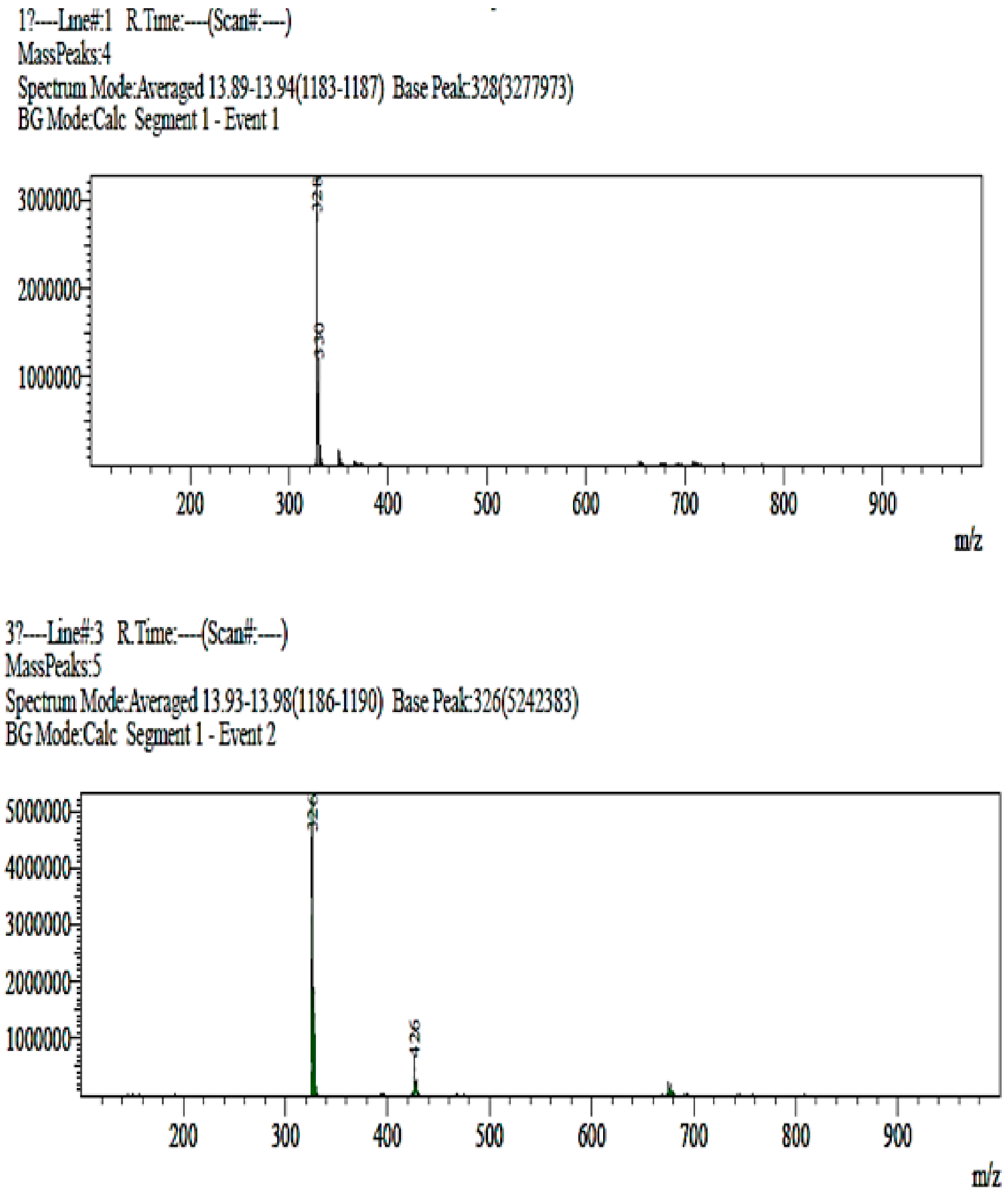


**Figure S28.** LC-Mass spectrum of compound **16b.**

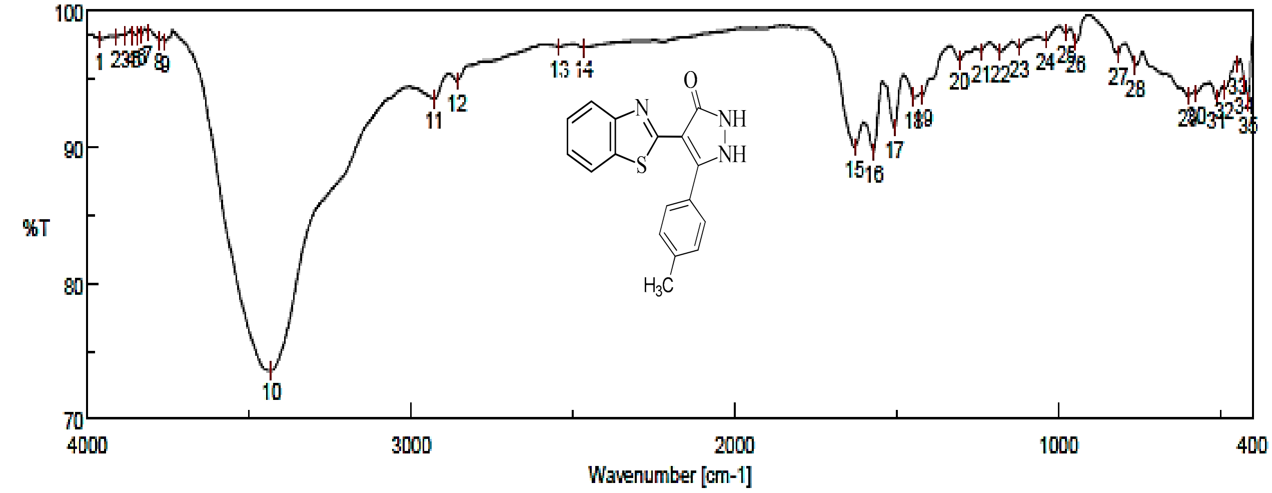


[ Result of Peak Picking ]

| No. | Position | Intensity | No. | Position | Intensity | No. | Position | Intensity | No. | Position | Intensity |
|---|---|---|---|---|---|---|---|---|---|---|---|
| 1 | 3963 | 97.9114 | 2 | 3913.82 | 98.0911 | 3 | 3883.93 | 98.1891 | 4 | 3863.68 | 98.1693 |
| 5 | 3848.26 | 98.1615 | 6 | 3833.79 | 98.2652 | 7 | 3815.47 | 98.4996 | 8 | 3778.83 | 97.8218 |
| 9 | 3761.47 | 97.719 | 10 | 3432.67 | 73.5324 | 11 | 2929.34 | 93.5087 | 12 | 2858.95 | 94.8699 |
| 13 | 2543.65 | 97.3935 | 14 | 2467.47 | 97.32 | 15 | 1629.55 | 89.9541 | 16 | 1570.74 | 89.6621 |
| 17 | 1507.1 | 91.4053 | 18 | 1447.31 | 93.6354 | 19 | 1419.35 | 93.8443 | 20 | 1305.57 | 96.3902 |
| 21 | 1238.08 | 97.0335 | 22 | 1180.22 | 97.0558 | 23 | 1121.4 | 97.3496 | 24 | 1040.41 | 97.8532 |
| 25 | 974.84 | 98.4627 | 26 | 946.877 | 97.7333 | 27 | 814.777 | 96.8383 | 28 | 762.709 | 96.0031 |
| 29 | 599.753 | 93.7655 | 30 | 573.719 | 93.9612 | 31 | 511.044 | 93.5699 | 32 | 486.938 | 94.258 |
| 33 | 445.476 | 96.1199 | 34 | 425.227 | 94.6436 | 35 | 412.692 | 93.2952 | | | |

**Figure S29.** IR spectrum of compound **16c.**

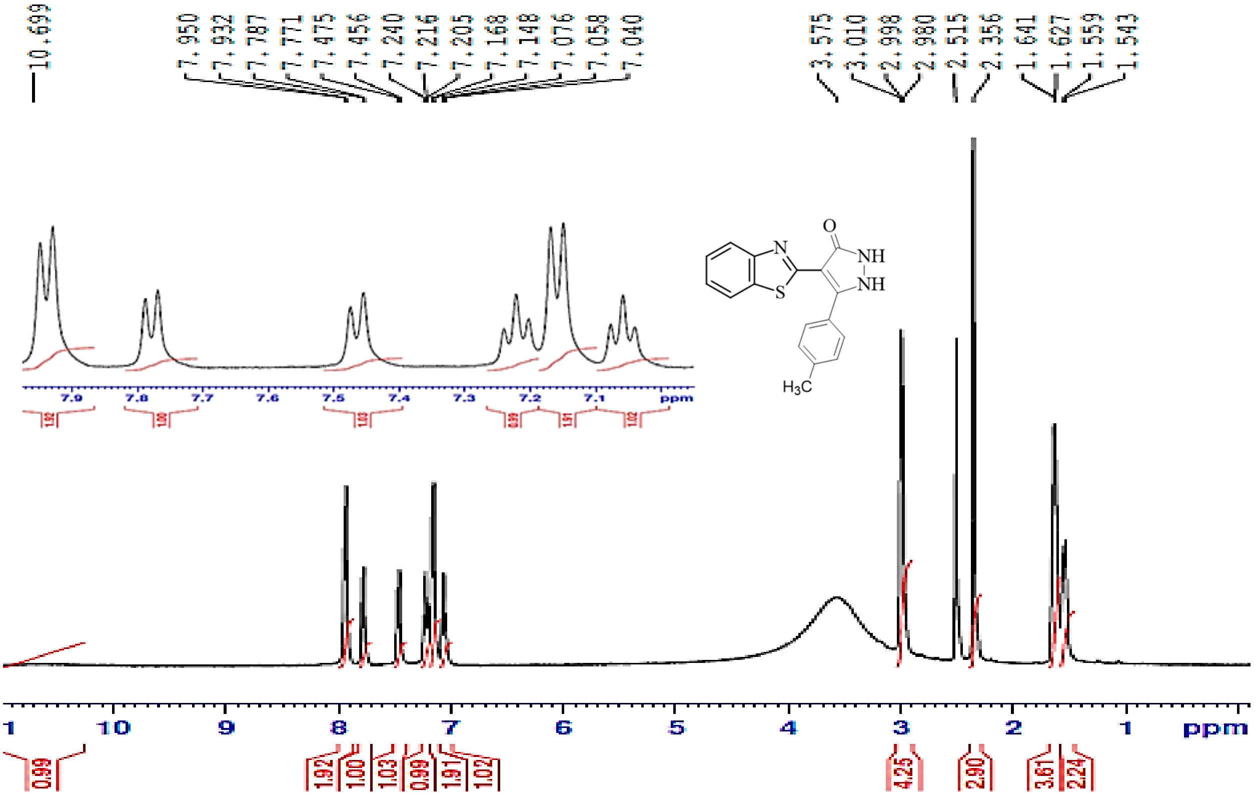


**Figure S30.** $^1$H NMR spectrum of compound **16c.**

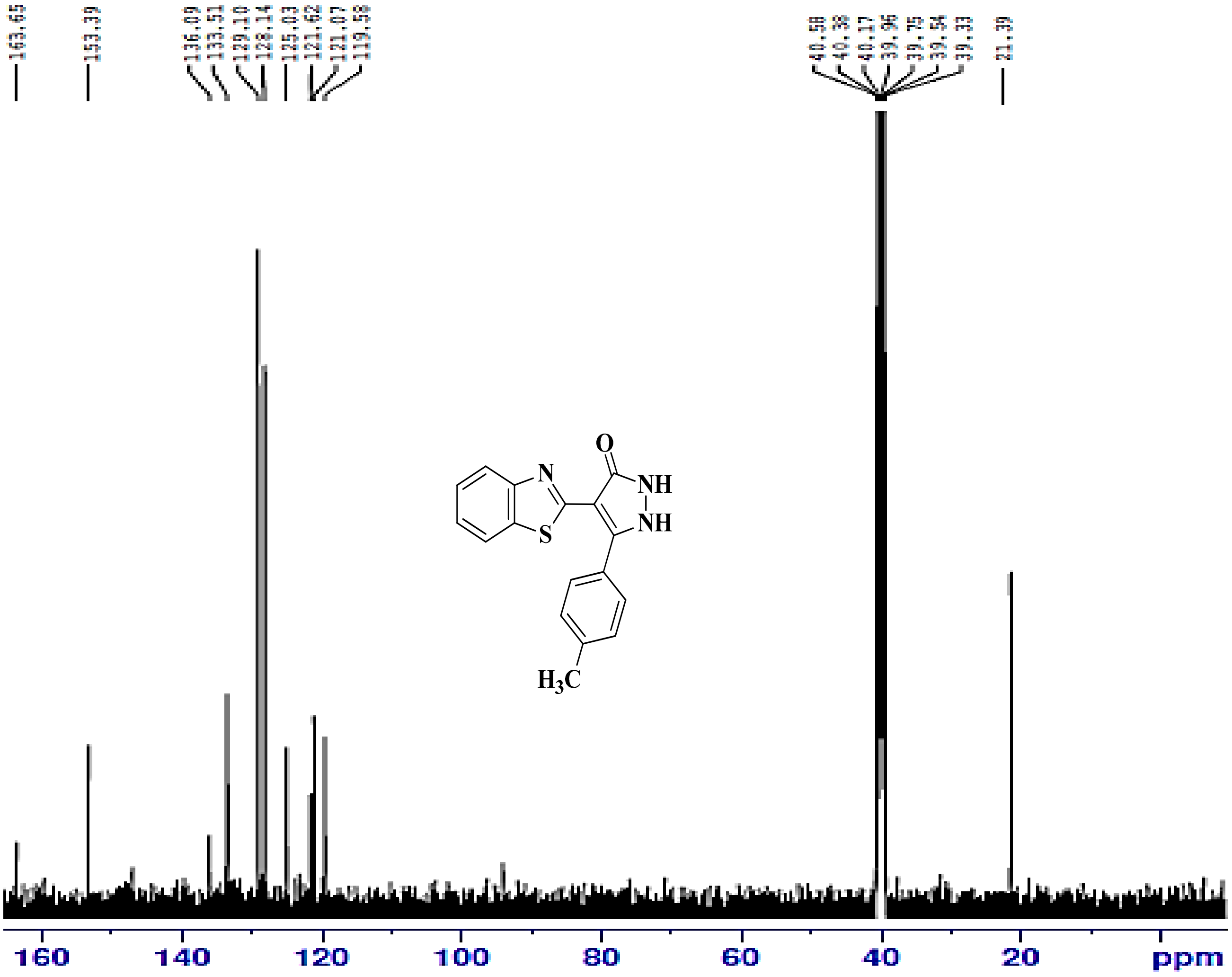


**Figure S31.** $^{13}$C NMR spectrum of compound **16c.**

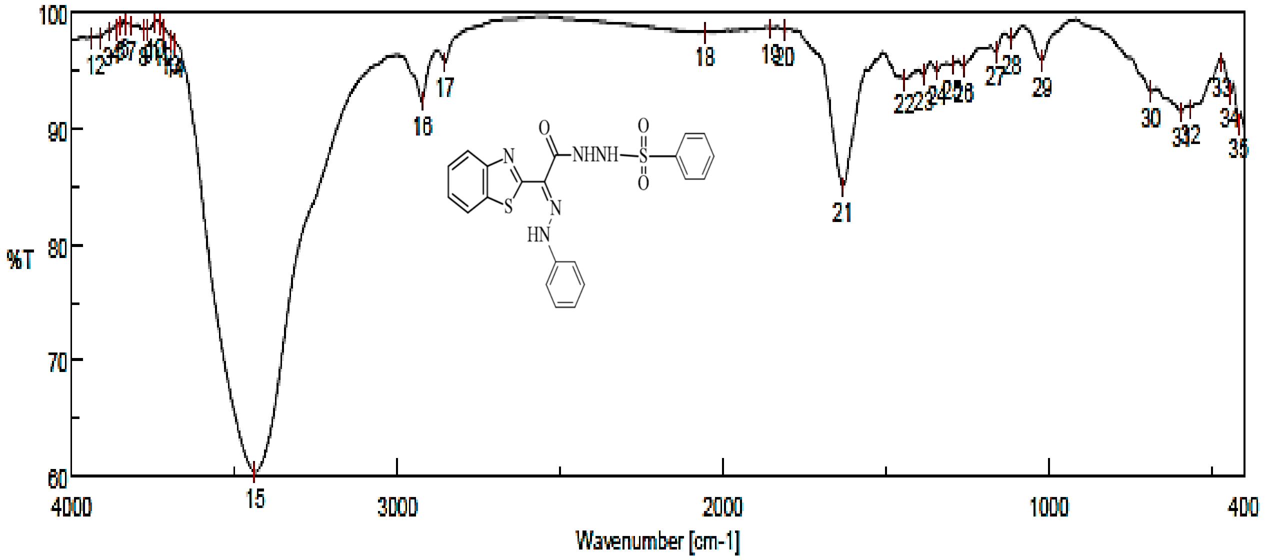


[ Result of Peak Picking ]

| No. | Position | Intensity | No. | Position | Intensity | No. | Position | Intensity | No. | Position | Intensity |
|---|---|---|---|---|---|---|---|---|---|---|---|
| 1 | 3941.79 | 97.8157 | 2 | 3911.9 | 97.7921 | 3 | 3887.79 | 98.4334 | 4 | 3863.68 | 98.5785 |
| 5 | 3850.18 | 98.8287 | 6 | 3837.65 | 99.0388 | 7 | 3816.44 | 98.8768 | 8 | 3779.8 | 98.4759 |
| 9 | 3766.3 | 98.5577 | 10 | 3745.08 | 99.1563 | 11 | 3730.62 | 98.9861 | 12 | 3719.05 | 98.4747 |
| 13 | 3697.84 | 97.7726 | 14 | 3685.3 | 97.3559 | 15 | 3438.46 | 60.3578 | 16 | 2925.48 | 92.4228 |
| 17 | 2855.1 | 95.777 | 18 | 2056.71 | 98.337 | 19 | 1854.22 | 98.6218 | 20 | 1810.83 | 98.5089 |
| 21 | 1633.41 | 85.0151 | 22 | 1443.46 | 94.2644 | 23 | 1384.64 | 94.6727 | 24 | 1342.21 | 95.0617 |
| 25 | 1296.89 | 95.5472 | 26 | 1261.22 | 95.3719 | 27 | 1162.87 | 96.6175 | 28 | 1114.65 | 97.7731 |
| 29 | 1020.16 | 95.9097 | 30 | 686.534 | 93.1837 | 31 | 591.075 | 91.4825 | 32 | 565.041 | 91.7717 |
| 33 | 468.617 | 95.7479 | 34 | 442.583 | 93.018 | 35 | 415.585 | 90.3887 | | | |

**Figure S32.** IR spectrum of compound **19a.**

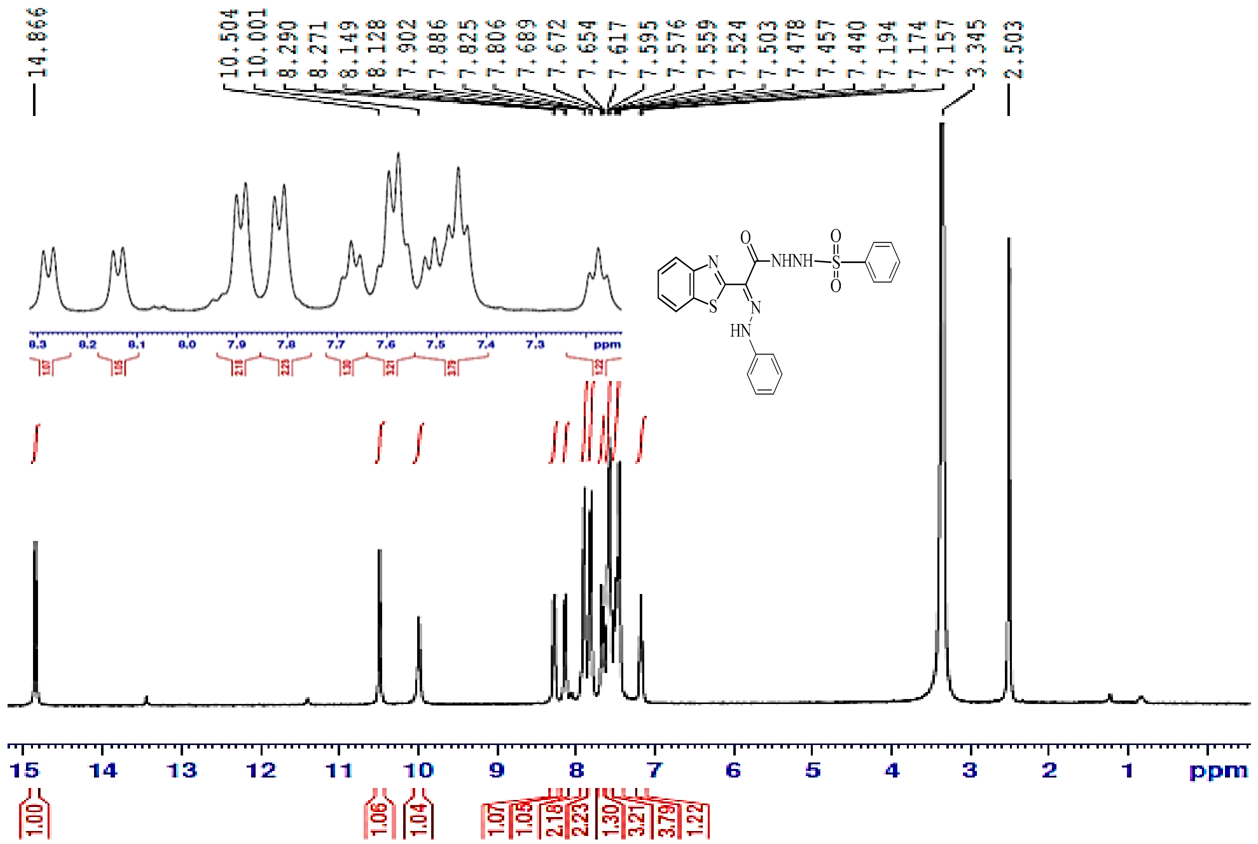

**Figure S33.** $^{1}$H NMR spectrum of compound **19a.**

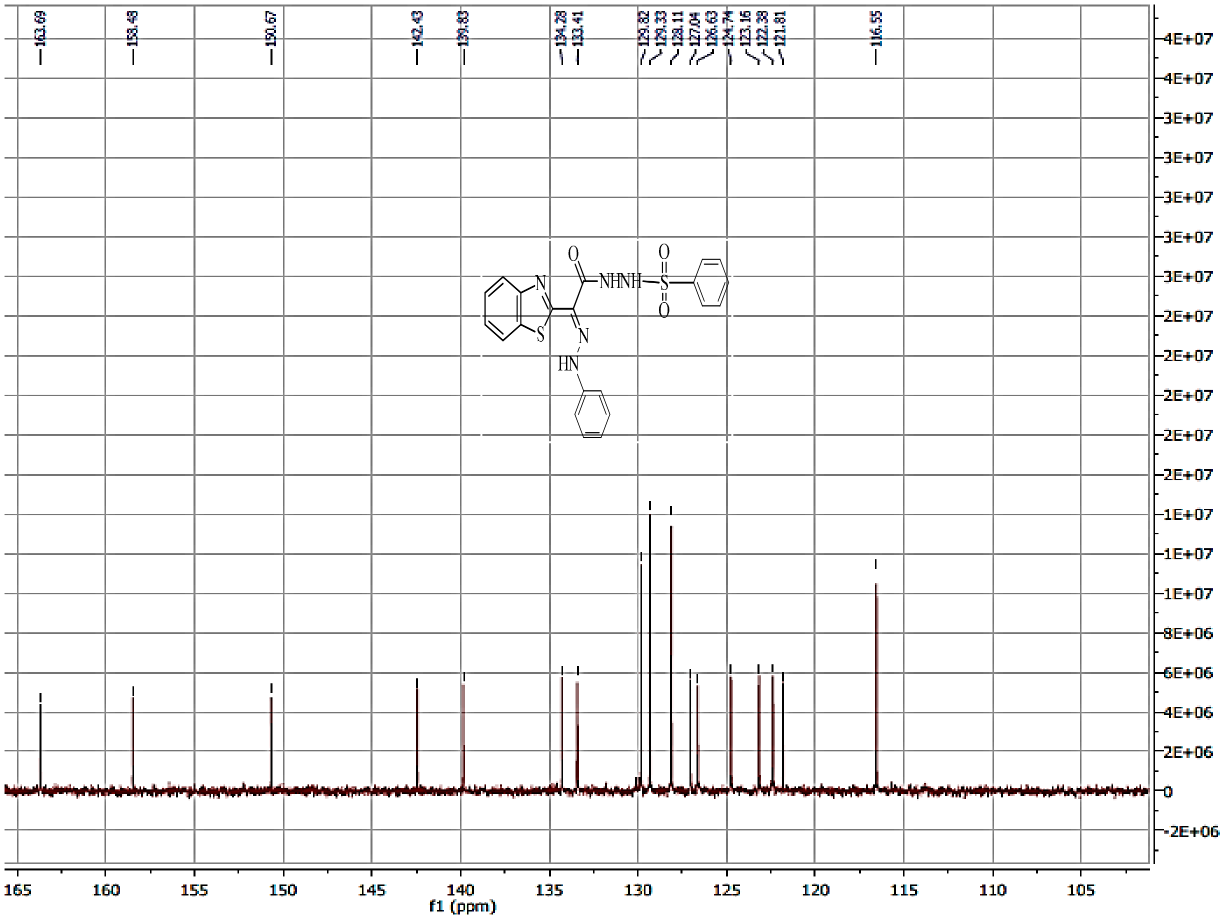

**Figure S34.** $^{13}C$ NMR spectrum of compound **19a.**

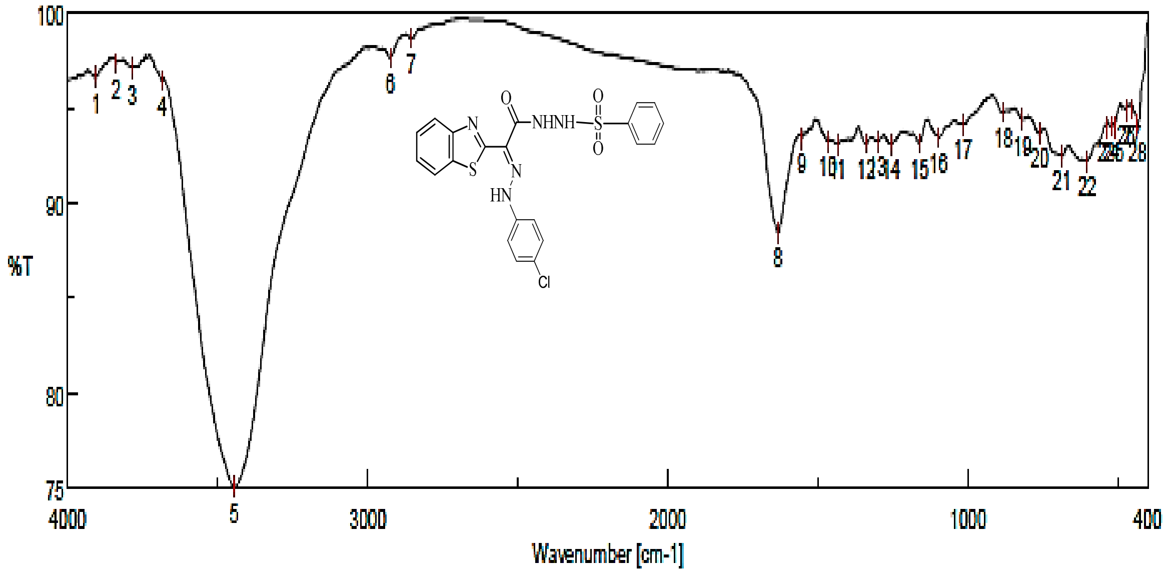


[Result of Peak Picking]

| No. | Position | Intensity | No. | Position | Intensity | No. | Position | Intensity | No. | Position | Intensity |
|---|---|---|---|---|---|---|---|---|---|---|---|
| 1 | 3905.15 | 96.6666 | 2 | 3839.58 | 97.3857 | 3 | 3783.65 | 97.1319 | 4 | 3686.26 | 96.4571 |
| 5 | 3445.21 | 75.0679 | 6 | 2925.48 | 97.6951 | 7 | 2855.1 | 98.6909 | 8 | 1632.45 | 88.461 |
| 9 | 1553.38 | 93.4337 | 10 | 1468.53 | 93.2721 | 11 | 1433.82 | 93.0954 | 12 | 1340.28 | 93.1188 |
| 13 | 1301.72 | 93.2908 | 14 | 1258.4 | 93.1109 | 15 | 1161.9 | 93.174 | 16 | 1097.3 | 93.4694 |
| 17 | 1016.3 | 94.1457 | 18 | 881.309 | 94.8192 | 19 | 818.634 | 94.4087 | 20 | 761.744 | 93.7013 |
| 21 | 687.498 | 92.5071 | 22 | 602.646 | 92.217 | 23 | 538.042 | 93.9944 | 24 | 522.615 | 94.0664 |
| 25 | 511.044 | 94.0206 | 26 | 472.474 | 94.9046 | 27 | 454.154 | 94.9934 | 28 | 434.869 | 94.1194 |

**Figure S35.** IR spectrum of compound **19b.**

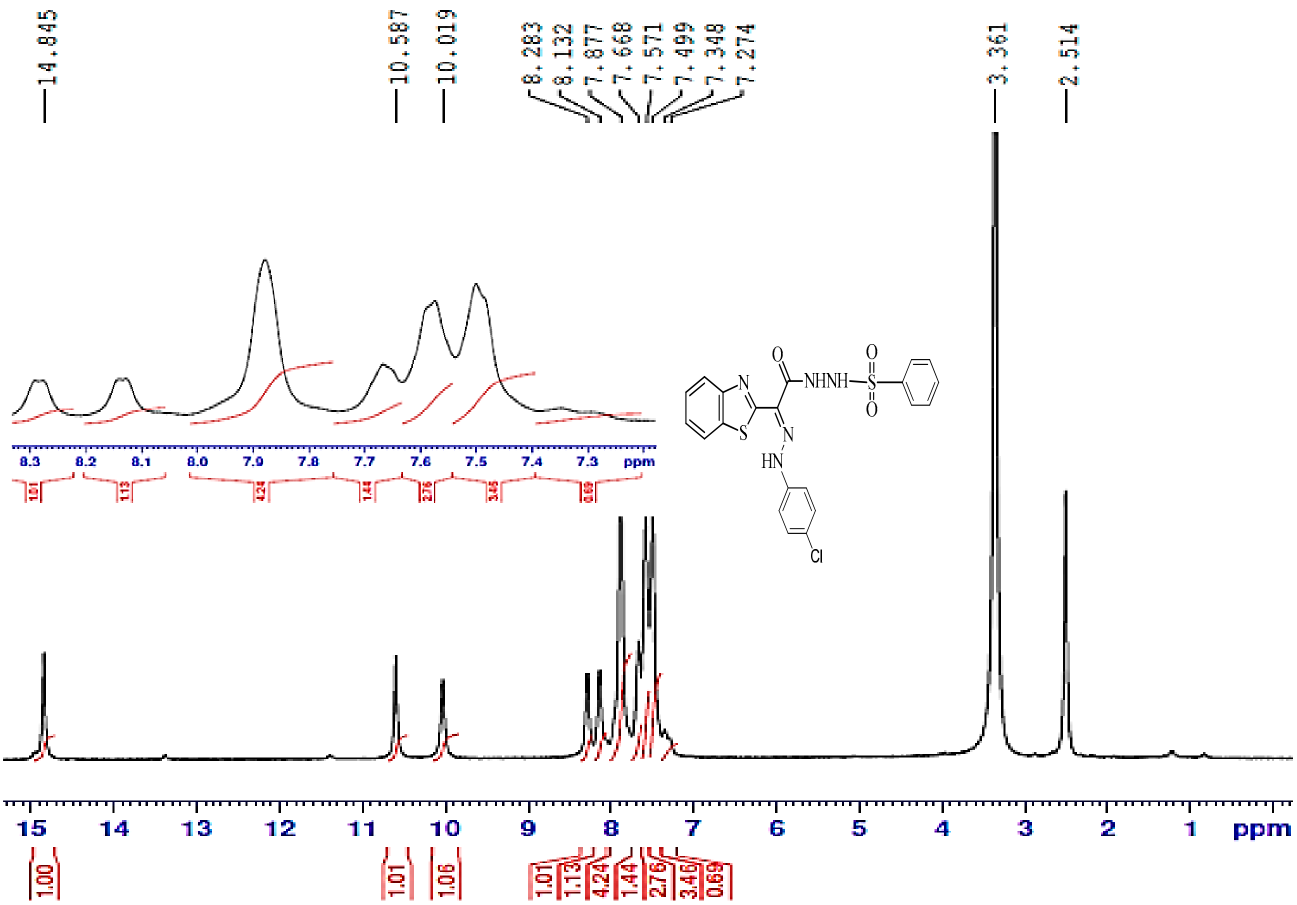


**Figure S36.** $^{1}$H NMR spectrum of compound **19b.**

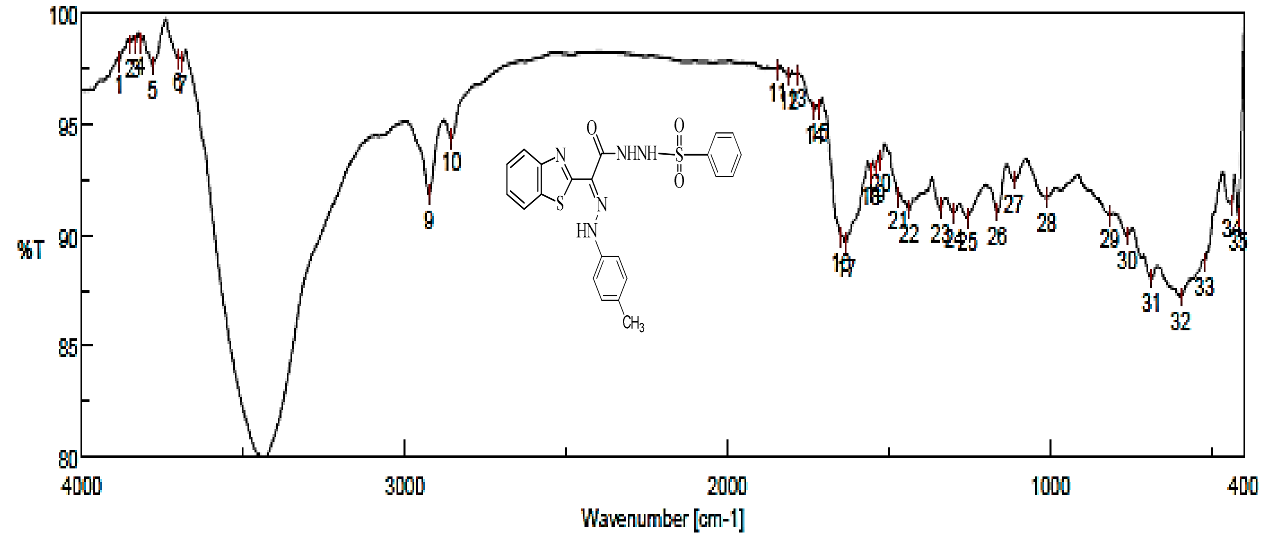


[ Result of Peak Picking ]

| No. | Position | Intensity | No. | Position | Intensity | No. | Position | Intensity | No. | Position | Intensity |
|---|---|---|---|---|---|---|---|---|---|---|---|
| 1 | 3884.9 | 97.8472 | 2 | 3852.11 | 98.5938 | 3 | 3834.76 | 98.5989 | 4 | 3818.36 | 98.6766 |
| 5 | 3781.72 | 97.6635 | 6 | 3703.62 | 97.966 | 7 | 3688.19 | 97.8593 | 8 | 3440.39 | 80.002 |
| 9 | 2925.48 | 91.8306 | 10 | 2856.06 | 94.364 | 11 | 1844.58 | 97.4712 | 12 | 1810.83 | 97.1678 |
| 13 | 1785.76 | 97.2437 | 14 | 1731.76 | 95.5973 | 15 | 1717.3 | 95.6697 | 16 | 1648.84 | 89.9072 |
| 17 | 1632.45 | 89.65 | 18 | 1555.31 | 92.8175 | 19 | 1540.85 | 92.9601 | 20 | 1524.45 | 93.4013 |
| 21 | 1470.46 | 91.7294 | 22 | 1438.64 | 91.1612 | 23 | 1340.28 | 91.2014 | 24 | 1297.86 | 90.9938 |
| 25 | 1256.4 | 90.7799 | 26 | 1163.83 | 91.0619 | 27 | 1112.73 | 92.4744 | 28 | 1010.52 | 91.702 |
| 29 | 815.742 | 90.8837 | 30 | 761.744 | 89.9665 | 31 | 686.534 | 88.0765 | 32 | 594.932 | 87.1965 |
| 33 | 520.686 | 88.7993 | 34 | 436.798 | 91.3569 | 35 | 416.549 | 90.848 | | | |

**Figure S37.** IR spectrum of compound **19c.**

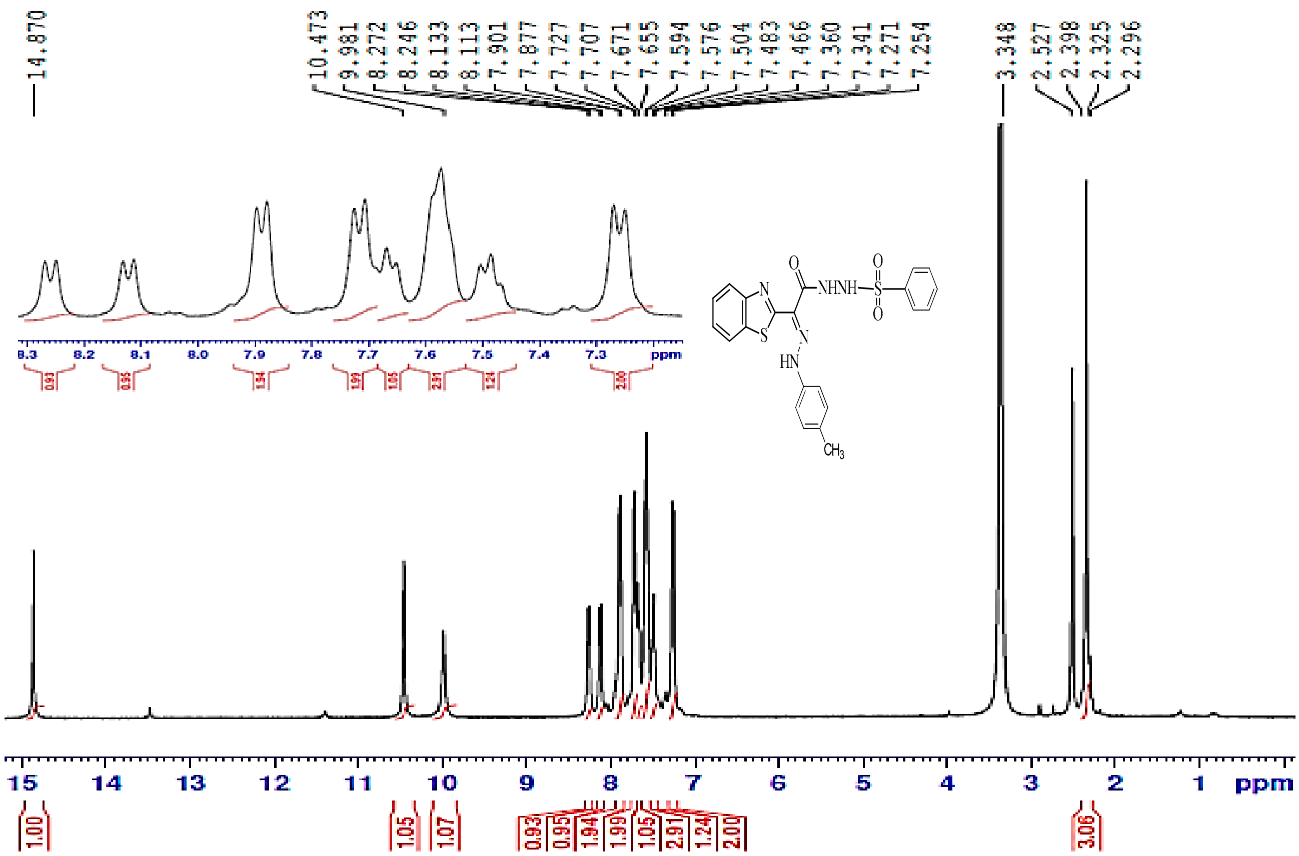

**Figure S38.** $^{1}$H NMR spectrum of compound **19c.**

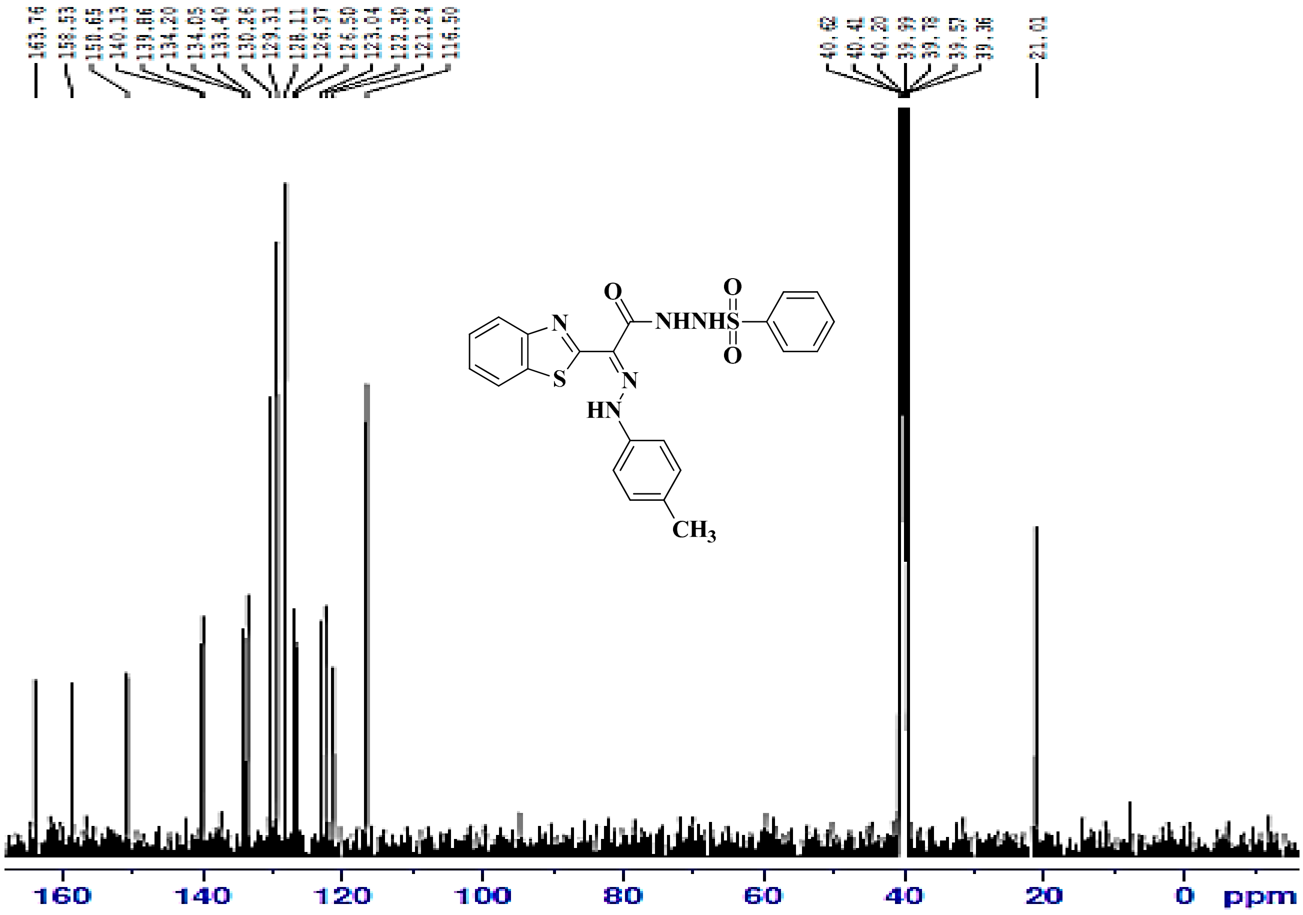

**Figure S39.** C NMR spectrum of compound **19c.**

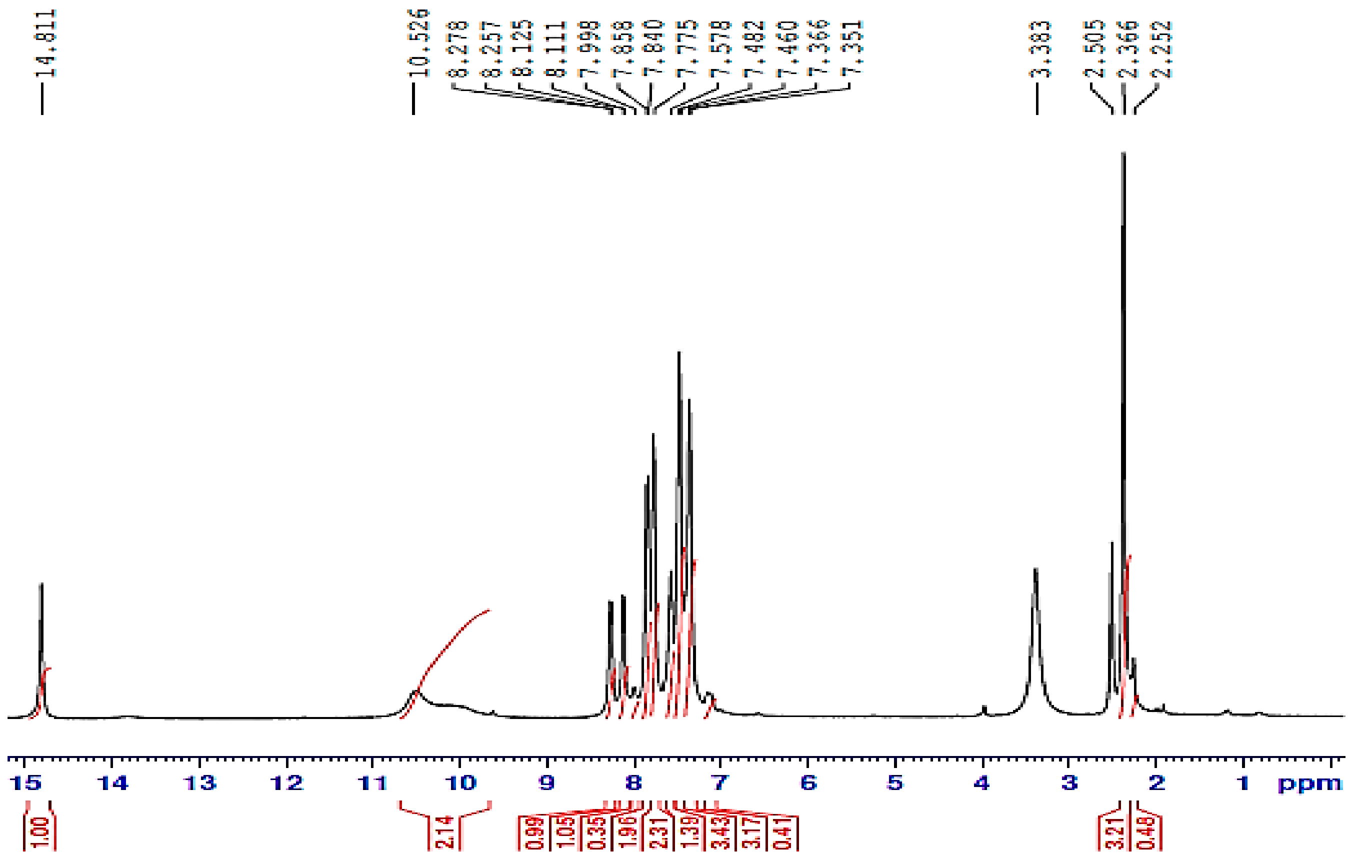


**Figure S40.** $^{1}$H NMR spectrum of compound **19d.**

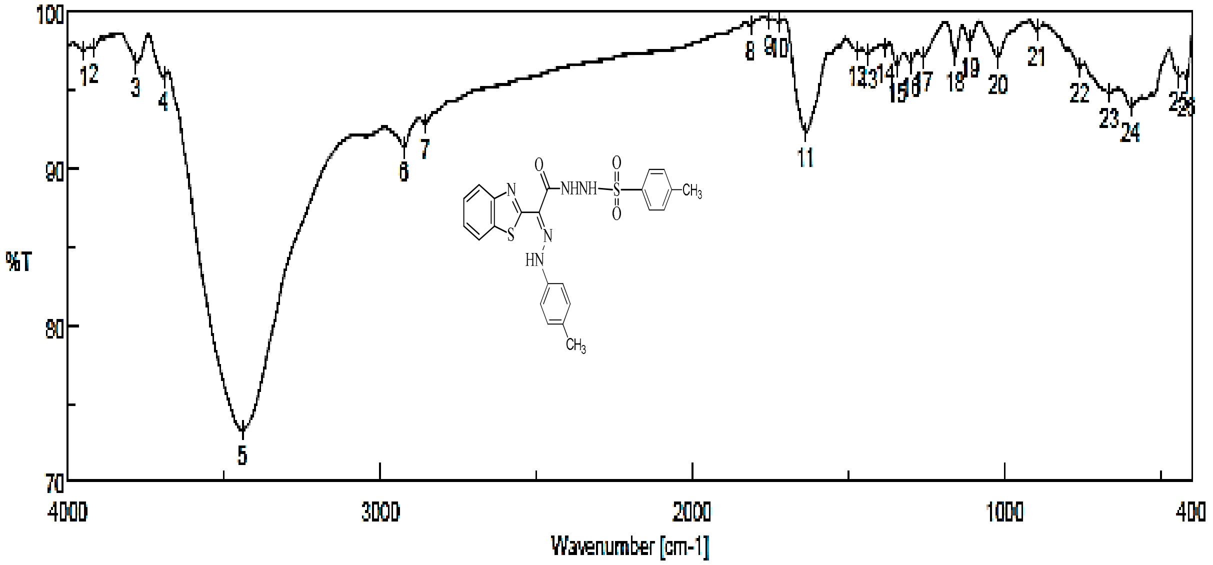


[ Result of Peak Picking ]

| No. | Position | Intensity | No. | Position | Intensity | No. | Position | Intensity | No. | Position | Intensity |
|---|---|---|---|---|---|---|---|---|---|---|---|
| 1 | 3950.46 | 97.5827 | 2 | 3917.68 | 97.7104 | 3 | 3782.69 | 96.7587 | 4 | 3693.98 | 95.8465 |
| 5 | 3442.31 | 73.2688 | 6 | 2924.52 | 91.3447 | 7 | 2856.06 | 92.8915 | 8 | 1813.72 | 99.1886 |
| 9 | 1753.94 | 99.5306 | 10 | 1723.09 | 99.3882 | 11 | 1636.3 | 92.3175 | 12 | 1472.38 | 97.539 |
| 13 | 1438.64 | 97.3152 | 14 | 1381.75 | 97.736 | 15 | 1345.11 | 96.5719 | 16 | 1300.75 | 96.784 |
| 17 | 1261.22 | 97.1375 | 18 | 1159.01 | 97.1938 | 19 | 1111.76 | 98.1311 | 20 | 1021.12 | 97.1048 |
| 21 | 894.809 | 98.8631 | 22 | 758.852 | 96.4637 | 23 | 664.357 | 94.8005 | 24 | 594.932 | 93.9454 |
| 25 | 441.619 | 95.8784 | 26 | 416.549 | 95.6441 | | | | | | |

**Figure S41.** IR spectrum of compound **19e.**

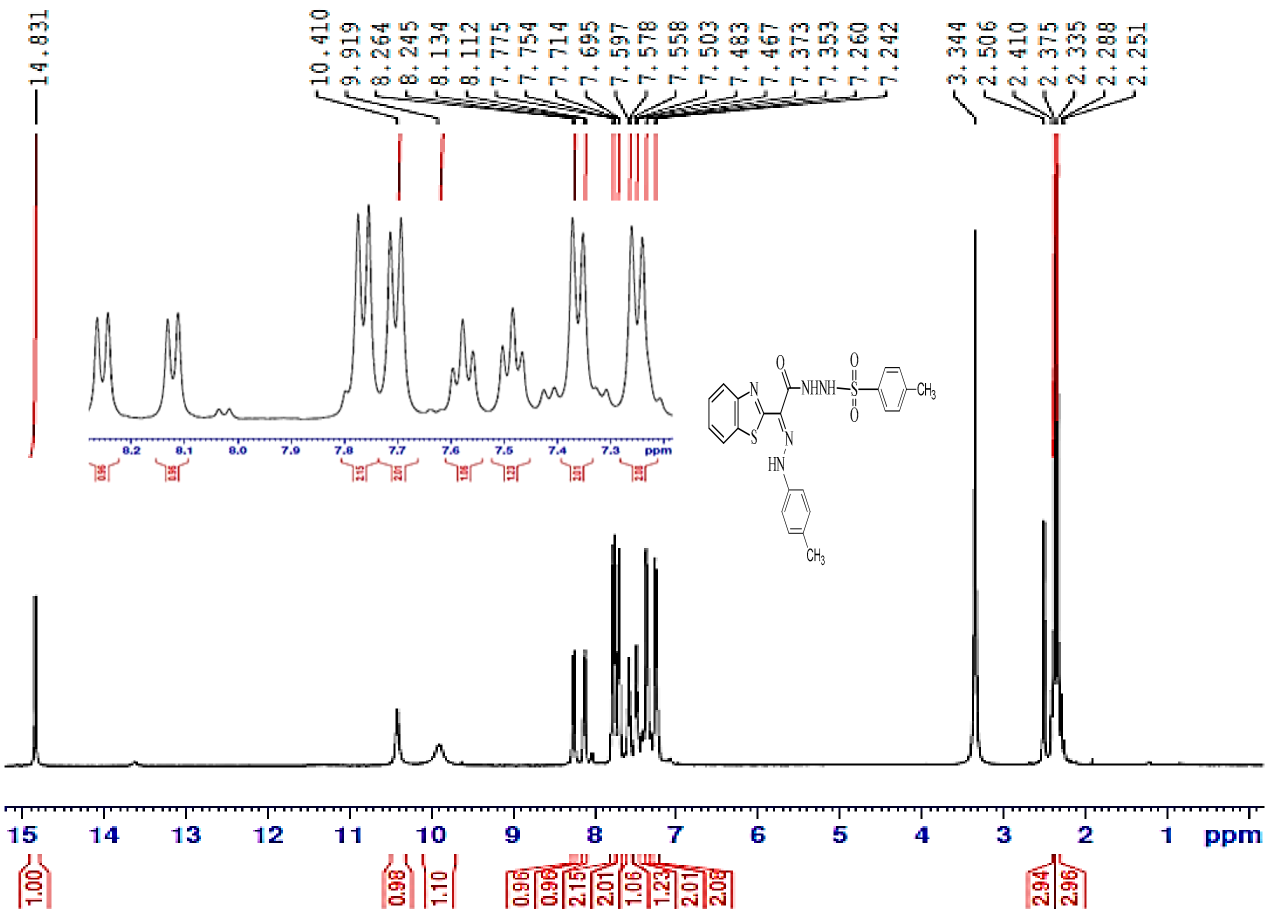


**Figure S42.** $^{1}$H NMR spectrum of compound **19e.**
